# Nuclear Localization of the Autism Candidate Gene Neurobeachin and Functional Interaction with the NOTCH1 Intracellular Domain Indicate a Role in Regulating Transcription

**DOI:** 10.1371/journal.pone.0151954

**Published:** 2016-03-21

**Authors:** Krizia Tuand, Pieter Stijnen, Karolien Volders, Jeroen Declercq, Kim Nuytens, Sandra Meulemans, John Creemers

**Affiliations:** 1 Department of Human Genetics, KU Leuven, Leuven, Belgium; 2 Leuven Autism Research consortium (LAuRes), KU Leuven, Leuven, Belgium; University of Toronto, CANADA

## Abstract

**Background:**

Neurobeachin (*NBEA*) is an autism spectrum disorders (ASD) candidate gene. NBEA deficiency affects regulated secretion, receptor trafficking, synaptic architecture and protein kinase A (PKA)-mediated phosphorylation. NBEA is a large multidomain scaffolding protein. From N- to C-terminus, NBEA has a concanavalin A-like lectin domain flanked by armadillo repeats (ACA), an A-kinase anchoring protein domain that can bind to PKA, a domain of unknown function (DUF1088) and a BEACH domain, preceded by a pleckstrin homology-like domain and followed by WD40 repeats (PBW). Although most of these domains mediate protein-protein interactions, no interaction screen has yet been performed.

**Methods:**

Yeast two-hybrid screens with the ACA and PBW domain modules of NBEA gave a list of interaction partners, which were analyzed for Gene Ontology (GO) enrichment. Neuro-2a cells were used for confocal microscopy and nuclear extraction analysis. NOTCH-mediated transcription was studied with luciferase reporter assays and qRT-PCR, combined with NBEA knockdown or overexpression.

**Results:**

Both domain modules showed a GO enrichment for the nucleus. PBW almost exclusively interacted with transcription regulators, while ACA interacted with a number of PKA substrates. NBEA was partially localized in the nucleus of Neuro-2a cells, albeit much less than in the cytoplasm. A nuclear localization signal was found in the DUF1088 domain, which was shown to contribute to the nuclear localization of an EGFP-DPBW fusion protein. Yeast two-hybrid identified the Notch1 intracellular domain as a physical interactor of the PBW domain and a role for NBEA as a negative regulator in Notch-mediated transcription was demonstrated.

**Conclusion:**

Defining novel interaction partners of conserved NBEA domain modules identified a role for NBEA as transcriptional regulator in the nucleus. The physical interaction of NBEA with NOTCH1 is most relevant for ASD pathogenesis because NOTCH signaling is essential for neural development.

## Introduction

Neurobeachin is a candidate gene for autism spectrum disorders (ASD), based on haploinsufficiency in several patients with ASD and its chromosomal localization in an ASD candidate region [1–8]. NBEA is a large multidomain protein, which is highly expressed in mouse brain starting from midgestation (E10.5) [9,10]. It has been suggested to affect the synaptic architecture through remodeling the actin cytoskeleton [11,12]. In zebrafish, NBEA is essential for the maintenance of extensive dendritic branching in mature neurons [13]. Furthermore NBEA affects postsynaptic neurotransmitter receptor trafficking to the cell surface and is a negative regulator of regulated secretion [12,14]. The homolog of *Nbea* in *D*. *melanogaster*, *Rugose (Rg)*, is important for associative learning and also affects synaptic architecture [15]. Based on a rough eye phenotype in *Rg* mutants, genetic interactions were observed with components of the Delta-Notch pathway [16–18]. Studying the homolog of *Nbea*, *SEL-2*, in vulval precursor cells of *C*. *elegans* also revealed a genetic interaction with the Notch pathway as well as the EGFR pathway [19].

**Fig 1:**
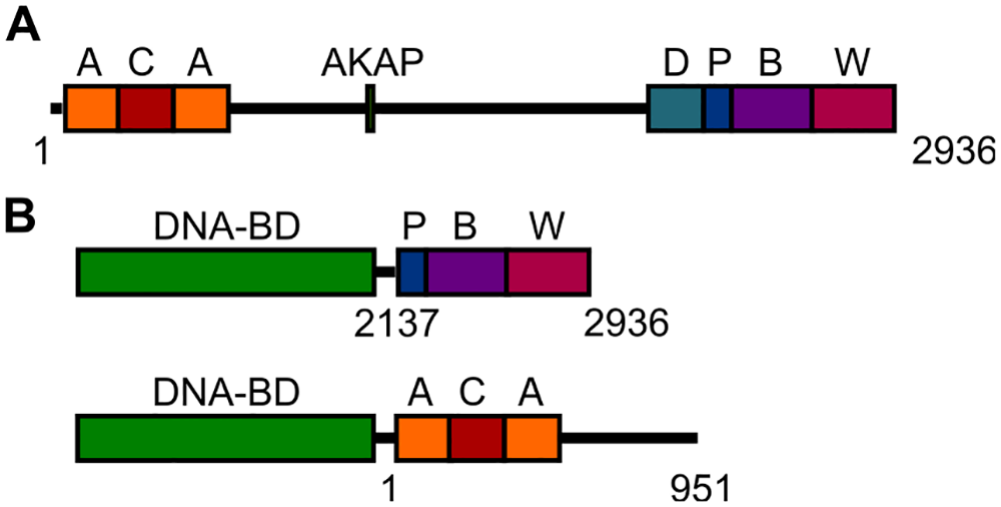
NBEA domain structure and constructs used. **A)** The NBEA protein [GenBank: 158854037] contains several conserved domains. From N- to C-terminus it contains a concanavalin A-like lectin domain (C) flanked by armadillo repeats (A), an A-kinase anchoring protein domain (AKAP), a domain of unknown function DUF1088 (D), the pleckstrin homology-like domain (P), followed by the BEACH domain (B) and WD40 repeats (W). The cDNA coding for this protein was used in a pcDNA3.1-FLAG plasmid for overexpression of full length NBEA. **B)** For the Y2H screens, two different well-conserved NBEA domain modules, the ACA or the PBW modules, were fused C-terminally to a DNA-binding domain (DNA-BD). The numbering indicates the range of amino acids of NBEA that are included.

NBEA contains the following domains: a concanavalin A-like lectin (CALL) domain flanked by armadillo repeats (ACA), an A-kinase anchoring protein domain (AKAP), a conserved domain of unknown function (DUF1088), a pleckstrin homology-like domain (PH-like) and the beige and Chediak-Higashi domain (BEACH) followed by WD40 repeats (Fig 1A) [10,20,21]. The N-terminal ACA domain module of NBEA may fulfill a scaffolding function, since Armadillo-repeats are known to mediate protein-protein interactions. The role of the concanavalin A-like lectin domain is unknown, but based on its high similarity to the N-terminal heavy chain of clostridial neurotoxins it might play a role in intracellular sorting [20].

NBEA is a member of the BEACH family of proteins, containing the conserved BEACH domain. Based on the limited knowledge about the protein functions in this family, it is thought that BEACH proteins are important for membrane dynamics and/or vesicle trafficking and NBEA has been found to negatively regulate secretion of dense-core granules [14,22]. It is still not known exactly how BEACH proteins contribute to these functions. Recently another BEACH protein, WD repeat and FYVE domain containing protein 3 (WDFY3), has been associated with ASD. WDFY3 was categorized into the transcription regulation network that seems to be implicated in ASD [23]. Specifically in NBEA and its mammalian homolog LPS-responsive beige-like anchor protein (LRBA) this BEACH domain is N-terminally preceded by the DUF1088 domain and the PH-like domain. An interaction between the PH-like and the BEACH domain has been reported and suggested to form a groove for binding of proteins [21]. The BEACH domain is followed by WD40 repeats, which are thought to be important for scaffolding. Together these domains will be addressed as a conserved DUF-PH-like-BEACH-WD40 (DPBW) domain module or without DUF1088 the PH-like-BEACH-WD40 (PBW) domain module.

The AKAP domain of NBEA can bind the regulatory subunit of protein kinase A (PKA) [10,24]. Furthermore, NBEA regulates phosphorylation of a number of PKA substrates, including CREB and Calpain-2 [25,26]. AKAPs are known to regulate PKA subcellular localization through its binding. Some AKAPs have been reported to bind to specific PKA substrates, bringing them in closer proximity to PKA for phosphorylation [27].

The cellular function of NBEA, how it affects regulated secretion and contributes to ASD pathogenesis remains elusive. Screening for protein interactors of the N- and C-terminal conserved domain modules of NBEA, may help to unravel the function of NBEA and to highlight networks that can be important in ASD. In this study, we performed a Y2H screen for the ACA and PBW domain modules of NBEA. The advantage of Y2H analysis is that weak and transient protein interactions can be discovered in addition to strong interactions. Pathway analysis of these interactors provided novel insights into the function of NBEA. Although one interaction was further validated by functional assays, most of these interactions should be interpreted as “not yet validated with other techniques”.

## Methods

### Identification of PBW or ACA domain module interactors by yeast two-hybrid screening

A partial region of mouse *Nbea* coding for the PBW domain module [GenBank: 158854037] (Asn2137-Tyr2936) was cloned into the bait yeast expression vector pB27 by Hybrigenics Services (France) (Fig 1B). pB27-PBW was transformed into the L40ΔGal4 yeast strain and Hybrigenics Services performed a Y2H screening of a mouse embryonic (E10.5 and E12.5) brain cDNA library (Hybrigenics) [28].

The Y2H screen for the ACA domain module of mouse *Nbea* was performed using the MATCHMAKER Two-Hybrid system (Clontech Laboratories Inc, CA, USA). The ACA domain module [GenBank: 158854037] (Met1-Met951) was cloned into the pGBKT7 plasmid (Fig 1B). This plasmid was co-transformed into the AH109 yeast strain together with a whole mouse embryonic (E12.5) cDNA pACT2 library [29], using the lithium acetate method [30]. After high stringency selection on synthetic dropout agar plates lacking leucine, tryptophane, and histidine (SD^---^), positive clones were restreaked on new SD^---^plates and grown for another 1 to 2 days. Only the prey plasmids of clones that survived the second high stringency selection round were extracted using the Prepease yeast plasmid isolation kit (Usb Corporations, OH, USA). The corresponding prey fragments were amplified by PCR and sequenced at their 5’ junctions. Sequences were then compared with the GenBank database using BLAST.

### Construction of expression plasmids

A pcDNA3.1-FLAG-Nbea plasmid, containing full length mouse *Nbea* cDNA [GenBank: 158854037] and an N-terminal fused FLAG-tag, was used as a template for a PCR with the following primers: 5’-GCTCTCGAATTCCTTGCATTTATTGAAC-3’ and 5’-CTGAGCGGATCCTCAGTACCTGTTCTG-3’. This PCR fragment contained the cDNA coding for the NBEA DPBW domain module [GenBank: 158854037] (Leu1948- Tyr2936) flanked by an EcoRI and a BamHI restriction site and was cloned in frame into the pEGFP-C2 plasmid, resulting in the pEGFP-DPBW plasmid.

The sequence coding for the five arginines forming a classical nuclear localization signal (NLS) in the DPBW domain module were mutated into alanines using two site-directed mutagenesis PCRs on the pEGFP-DPBW template with the Quikchange Site-directed Mutagenesis Kit (Stratagene, CA, USA) (S1 Table). Mutations were sequence verified using Sanger sequencing.

### Interaction network analysis

All interactors were uploaded into Cytoscape 3.2.0 to create a network of direct protein–protein interactions for the two separate domain modules [31].

The BiNGO plug-in was used to assess significant enrichment of gene ontology identifications (GO-ID) in the interactor list compared to the whole mouse genome (S2 and S4 Tables) [32]. The threshold for significance was set at 0.01. Non-redundant and brain tissue-related significant terms were selected to be of interest within the context of ASD.

On the 9^th^ of April 2015, a Pubmed literature search was performed to find publications linking any of the interacting proteins with ASD by combining the gene name with the term “autism” as a query. Full articles were then reviewed for a relevant link between the gene and ASD pathogenesis. All interacting proteins were used as a query in the Online Mendelian Inheritance in Man (OMIM) database.

The interacting proteins were identified as being PKA substrates if they were listed as such in the Phosphosite database [33].

### Bio-informatics analysis

The mouse NBEA full length FASTA protein sequence [GenBank: 158854037] was submitted into several bio-informatics tools (NucPred, PSORT II, PredictNLS and NetNES 1.1) to assess its nuclear localization and look for consensus nuclear localization signals [34,35].

### Cell culture and transfections

The mouse neuroblastoma cell line Neuro-2a (N2a) (CCL-131, obtained directly from American Type Culture Collection (ATCC), UK) or the human embryonic kidney 293 T cell-line (HEK293T) (CRL-3216, obtained directly form ATCC) were cultured in Dulbecco's modified Eagles medium supplemented with 10% fetal bovine serum at 37°C, 5% CO_2_. For overexpression, N2a cells were transfected using TurboFect Transfection Reagent (Life technologies, CA, USA), while HEK293T cells were transfected using Fugene 6 transfection reagent (Promega, WI, USA), both according to the manufacturer’s protocol. For NBEA knockdown, N2a cells were transfected with the SMARTpool siGENOME Nbea (26422) siRNA (S1 Table, GE Healthcare, UK) using Lipofectamine RNAiMax (Life technologies) according to the manufacturer’s protocol. The Stealth RNAi siRNA Negative Control, Med GC (Life technologies) was transfected as a control. Cells were harvested 24h after transfection, except for experiments with NBEA knockdown, where cells were harvested after 72h [14]. NBEA knockdown or overexpression was verified by means of a quantitative RT-PCR on mRNA of transfected N2a cells.

When co-transfecting with other plasmids, knockdown was obtained by transfection with the previously described mU6Pro vector containing shRNA targeting the part of *Nbea*, coding for the C-terminal region, from amino acid 1863 until 2566 (mU6Pro-shRNANbea) [14]. The construct used for NBEA overexpression was pcDNA3.1-FLAG-Nbea, expressing full length mouse NBEA.

### Luciferase reporter assay

The CBF1-dependent 4xwtCBF1 firefly luciferase reporter (4xwtCBF1Luc) has previously been used to asses NOTCH mediated transcription [36]. N2a cells were cotransfected with 390 ng of mU6Pro-shRNA-Nbea or pcDNA3.1FLAG-Nbea respectively, and with 320 ng of pCS2-NICD, 250 ng 4xwtCBF1Luc reporter and 40 ng pRL-TK plasmid. Control conditions were used for NBEA overexpression or knockdown, NICD overexpression and for 4xwtCBF1Luc overexpression by performing parallel transfections with the empty control plasmids or 4xmtCBF1Luc.

The Dual-Luciferase Reporter Assay System (Promega) was used to assess the effect of NBEA knockdown or overexpression on Notch-mediated transcription. To account for differences in transfection efficiencies, firefly luciferase activity was normalized the Renilla luciferase activity.

### RNA extraction and quantitative reverse transcription PCR (qRT-PCR)

RNA of N2a cell cultures was extracted with the Nucleospin RNA Midi kit (Macherey-Nagel, Germany) according to the manufacturer’s protocol. Per experiment, an equal amount of RNA was reverse transcribed to cDNA using the iScript cDNA synthesis kit (Bio-Rad, CA, USA). Real-time qPCR was performed using iQ SYBR Green Supermix (Bio-Rad). 40 cycles of annealing/extension for 1min at 60°C were carried out with the MyiQ single color real-time PCR detection system (Bio-Rad). The relative amount of mRNA expression was calculated using the generalized qBase method [37]. In this method normalization is performed to the geometric mean of multiple reference genes. In this case two reference genes were used, *peptidylprolyl isomerase A (Ppia)* and *growth arrest and DNA-damage-inducible alpha (Gadd45a)*. The primers used are given as S2 Table.

### Nuclear extraction

The Nuclear Complex Co-IP kit (Active Motif, Belgium) was used according to the manufacturer’s protocol to obtain cytoplasmic and nuclear fractions of N2a cells.

### Co-immunoprecipitation

Cells were harvested in lysis buffer (0.1% Triton-X100; 150 mM NaCl; 50mM Tris-HCl pH 7.4 and cOmplete, Mini, EDTA-free Protease Inhibitor cocktail (Roche, Germany)) and briefly sonicated on ice. After sonication cell debris was removed by centrifugation. Per sample, 20μl of a 50% bead slurry of protein G agarose beads (GE Healthcare) were used and washed twice with PBS before use. All incubations were performed at 4°C on a mechanical rotator. Protein G agarose beads were incubated with anti-GFP antibody (Roche) for 1h, after which they were incubated with PBS with 3% bovine serum albumine (BSA) (Sigma-Aldrich, MO, USA) for 1h. Beads were washed with lysis buffer after which cell lysate was added for 2h. Beads were washed five times with lysis buffer and proteins were harvested by resuspending the beads in sample buffer (50mM Tris-HCl pH 7; 10% glycerol; 2% SDS; bromophenol blue) compatible with immunoblot analysis.

### Immunoblotting

A bicinchoninic acid protein assay (Life technologies) was used to determine the protein concentration of the samples. Samples were dissolved in SDS sample buffer. Depending on the molecular weight of the proteins of interest a 10% Bis-Tris gel (Bio-Rad) or 3–8% Tris-Acetate gel (Life technologies) was loaded with 25μg of each sample. Proteins were transferred to Protran Nitrocellulose membrane (Schleicher&Schuell, Germany) and incubated with antibodies against endogenous NBEA (1/3,000; rabbit polyclonal, homemade against the synthetic peptide TKVSDDILGNSDRPGS-Cys-KLHMBS (Eurogentec) as validated in [14] and in S1 Fig), GAPDH 14C10 (1/5,000; rabbit mAb, #2118S, Cell signaling technology), HDAC2 (1/2,000; rabbit polyclonal, sc-7899, Santa-Cruz Biotechnology Inc, CA USA), Myc (1/5000; mouse 9E10 mAb, home-made) and GFP (1/1,000; mouse mAb, 11814460001, Roche, Germany). Afterwards, membranes were incubated with HRP-conjugated secondary antibody (1/2,000; Dako, Denmark) and proteins were visualized with western blotting ECL detection reagent (Life technologies). Densitometry was performed using Image J analysis software (NIH) [38].

### Confocal microscopy and image analysis

After washing with PBS, N2a cells were fixed for 30 min with 4% formaldehyde (Polysciences, PA, USA) in PBS at room temperature. Cells were washed three times with PBS, followed by permeabilization with 0.1% Triton-X100 (Acros) in PBS for 10 min. After blocking for 20 min with 5% goat serum (Dako) and 1% BSA (Sigma-Aldrich) in PBS (blocking buffer), cells were incubated with the primary homemade antibody for endogenous NBEA (1/1,000), diluted in blocking buffer, for 1h. After three washes, cells were incubated with a goat secondary Alexa594- or Alexa488-conjugated antibody (Life technologies) and DAPI (1/10,000) for 1h. Finally the cells were washed and mounted on microscope slides using Vectashield mounting medium (Vector laboratories, Canada). Cells were analyzed using the Olympus Fluoview FV1000 confocal laser scanning microscope and a 63x immersion oil objective.

The tool ‘Z project’ in ImageJ software was used to make a 2D Z-projection of a confocal Z-stack. All confocal images shown are single confocal planes, unless otherwise specified.

Quantification of nuclear EGFP fluorescence intensity was performed using Cell Profiler cell analysis software [39]. At least 300 transfected cells were measured per condition.

### Statistical analysis

Data are represented as mean ± standard error of the mean (SEM). A student’s *t*-test was used, unless stated otherwise. All statistical tests were performed with 0.05 as the α-level of significance unless stated otherwise. With * *P* < 0.05, **P < 0.01 and ***P < 0.001.

## Results

### The NBEA PBW domain module interacts with nuclear proteins

**Fig 2:**
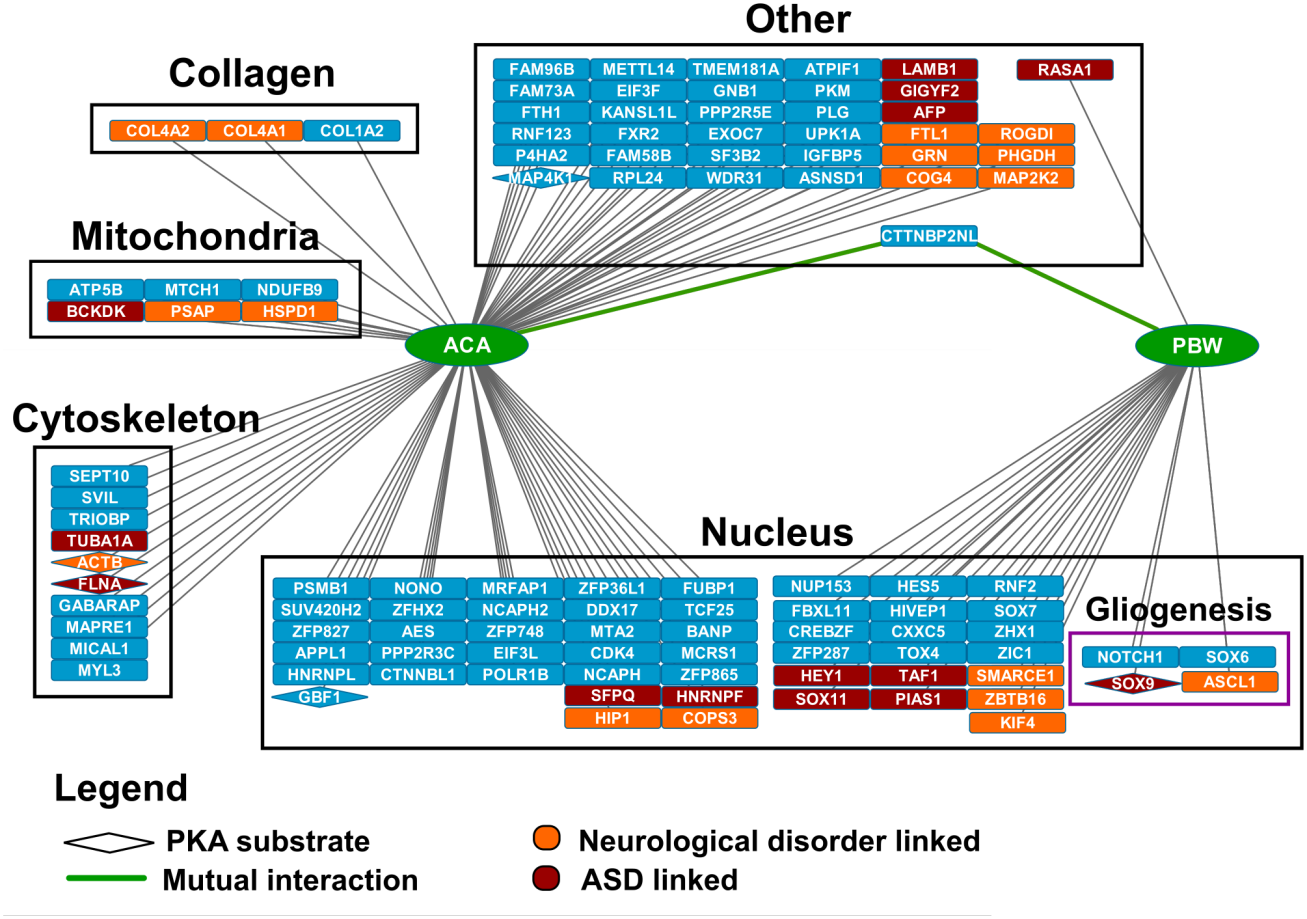
Y2H screening results for the ACA and PBW domain module. The ACA and PBW domain modules of NBEA were used to screen for possible interaction partners. The results were visualized with Cytoscape software. Color coding was used to show genes related to ASD (red), to other neurologic disorders (orange) or no known association (blue). BiNGO enrichment analysis picked up an enrichment of ACA-interacting proteins for the cellular components: Cytoskeleton, Nucleus, Mitochondria, Collagen and Cytoplasm. The enrichment for cytoplasm is not shown in the figure. BiNGO analysis of PBW-interacting proteins only picked up the nucleus as an enriched cellular component. Some genes in this compartment also showed enrichment for the biological process of glial cell differentiation or Gliogenesis (purple framework). Proteins that are grouped as ‘Other’ were cytoplasmic or not picked up in the mentioned enrichments for cellular component. The rhomboid shape marks PKA substrates. The mutual interaction partner is connected with a green line.

A Y2H screen for the two conserved domain modules ACA and PBW resulted in a list of 83 and 25 possible interactions, respectively (Fig 2, S3 and S4 Tables). Fourteen of these proteins (13%) have previously been reported within the context of ASD. In total, 31 genes (29%) are associated with neurological disorders. Five known PKA substrates (FLNA, ACTB, SOX9, MAP4K1 and GBF1) interacted with the domain modules of NBEA and three of these are linked with neurological disorders (FLNA, ACTB, SOX9). Both the PBW and the ACA domain interacted with Cortactin binding protein 2 N-terminal like (CTTNBP2NL). A lot of detected interactions were single hits, which highlights the need for further validation of these interactions in future experiments.

A search for GO-ID enrichment in the ACA or PBW interactor group compared to the whole mouse genome was performed with BiNGO. For the ACA interactor group a significant enrichment was found for the following cellular components: cytoplasm (53%; *P* = 2.3461E-7), nucleus (39%; *P* = 2.2586E-6), cytoskeleton (13%; *P* = 4.3418E-3), mitochondrial part (8%; *P* = 7.1712E-3), and collagen (4%; *P* = 1.5926E-4) (Fig 2, S5 Table).

For the PBW interactors there only seemed to be a significant enrichment for proteins present in the nucleus (92%; *P* = 2.8728E-15) (Fig 2, S6 Table). These interactors mostly have a molecular function in DNA binding (84%; *P* = 7.5905E-21) and/or transcription regulator activity (60%; *P* = 2.7411E-14). The PBW domain module also interacted with NUP153, which is a nuclear pore protein (Fig 2). Within the context of ASD it is of interest that there was a significant enrichment for biological processes in glial cell differentiation (17%; *P* = 9.8389E-7) and the NOTCH signaling pathway (13%; *P* = 5.7435E-5) (S6 Table).

### Nuclear presence of NBEA

Neurobeachin is known to be expressed in the cytoplasm and localized near the *trans*-Golgi network in a punctate pattern. Since the NBEA PBW domain module and even the ACA domain module interacted with a large number of nuclear proteins, we first used bio-informatics to assess whether full length NBEA could be present in the nucleus. PSORT II and NucPred detected classical nuclear localization signals (NLS), although PredictNLS failed to detect any nuclear localization (Table 1, S1 Appendix). NucPred gave a nuclear localization score of 0.56, meaning there is a 70% chance of nuclear localization. No leucine-rich nuclear export signals (NES) were identified (S2 Appendix).

**Table 1:** PSORT II Computer prediction of putative NLSs in mouse NBEA.

PSORT II Prediction	Location^a^	NBEA domain	Pattern^b^	Consensus	NLS type
HKRK	768	-	4	(H/P)(K/R)_n_	SV40-like
RRRR	2066	DUF1088	4	(K/R)_4_	SV40-like
RRRR	2067	DUF1088	4	(K/R)_4_	SV40-like

^a^ Amino acid position in NBEA (NP_085098.1) of the first residue in the predicted motif.

^b^ Pattern names employed by PSORT II, which were correlated to a consensus NLS sequence as described in the consensus column, were used for comparison with the primary sequence of NBEA.

**Fig 3:**
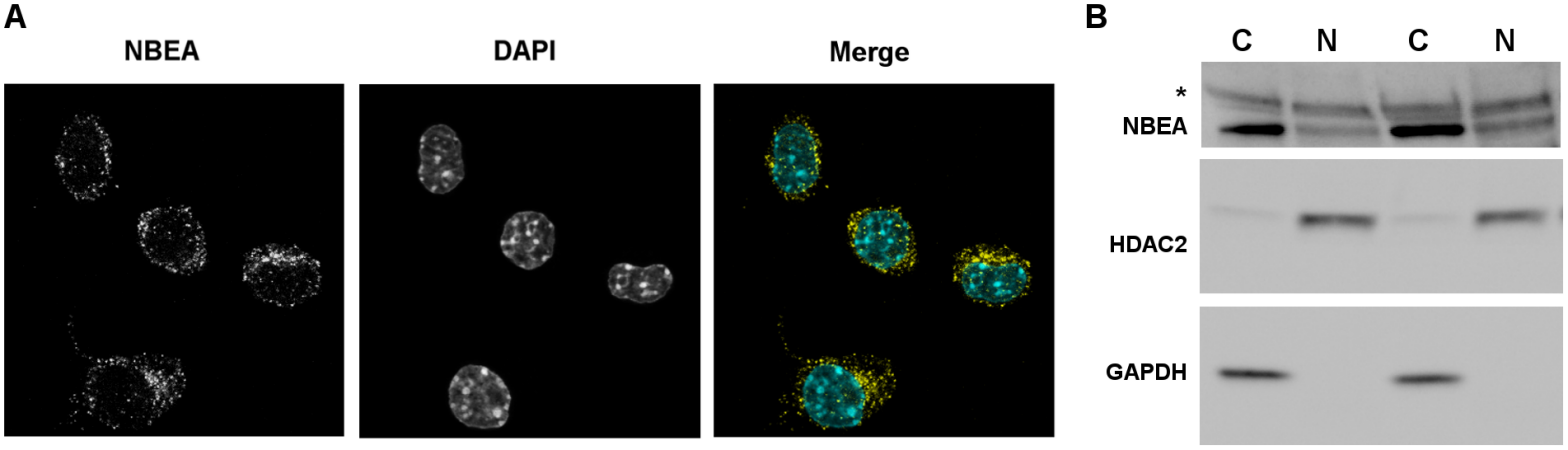
Nuclear localization of NBEA. **A)** Confocal image of endogenous NBEA (yellow) in N2a cells. DAPI (cyan) was used to stain the nucleus. The separate images are shown in greyscale. **B)** Western blot showing endogenous NBEA at 327 kDa in cytoplasmic (C) and nuclear (N) fractions of N2a cells. GAPDH and HDAC2 were used as cytoplasmic and nuclear markers, respectively. * Aspecific band at 460 kDa.

In N2a cells, low levels of NBEA (nuclear fraction of 19.1% ± 0.8) were detected in the nucleus, in addition to the previously described cytoplasmic localization using confocal microscopy (Fig 3A, S2 and S3 Figs, S1 Mov) [10]. This nuclear presence was confirmed by western blot of nuclear fractions (Fig 3B). Furthermore, the online database “Human protein atlas” supports these data, since immunostaining with a different NBEA antibody also shows NBEA expression in the nuclei of SH-SY5Y, human neuroblast, cells (S4 Fig) [40].

Stimulating the cAMP/PKA pathway in N2a cells with Forskolin and IBMX or inhibiting nuclear export with Leptomycin B did not increase the nuclear presence of NBEA (data not shown). The latter suggests that NBEA is not actively exported out of the nucleus by the exportin CRM1, consistent with the fact that no consensus NES was detected.

### The DUF1088 domain in NBEA is important for nuclear localization

**Fig 4:**
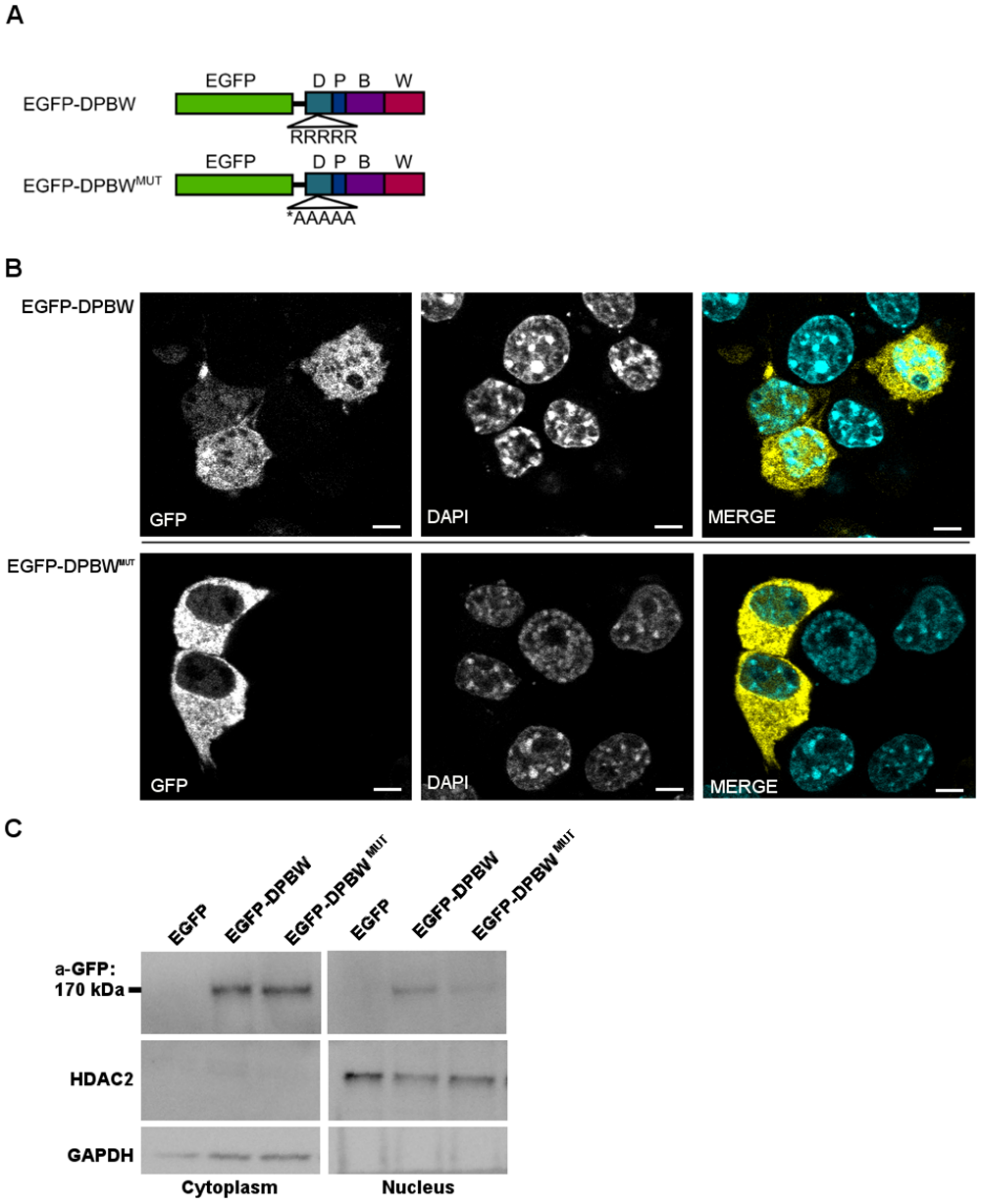
The DUF1088 domain of NBEA contains a NLS. **A)** EGFP-fused constructs were made containing the DPBW domain module. The NLS determined using bio-informatics is shown in an inset. For the EGFP-DPBW^MUT^ construct the arginines (R) of the EGFP-DPBW construct were mutated to alanines (A). **B)** Representative confocal image of N2a cells with overexpression of the EGFP-DPBW or the EGFP-DPBW^MUT^ fusion protein. DAPI was used to stain the nucleus. Quantification of nuclear intensity was based on at least 300 transfected cells per condition. Scale represents 5μm. **C)** Representative western blot of four experiments showing nuclear and cytoplasmic fractions of N2a cells transfected with pEGFP, pEGFP-DPBW or EGFP-DPBWMUT. For detection of the EGFP-fusion protein (170 kDa) an anti-GFP antibody was used (a-GFP). As a control for specificity for the fusion protein, no signal is seen for the unfused EGFP protein at this height, since it has a lower molecular weight of 27–30 kDa. HDAC2 and GAPDH were used as markers for nuclear and cytoplasmic fractions, respectively.

A penta-arginine sequence located in the DUF1088 domain was identified as a potential nuclear localization signal by NucPred and PSORT II (Fig 4A, Table 1, S1 Appendix). An EGFP-DPBW fusion protein was designed to determine its localization. The EGFP-DPBW fusion protein was detected in the cytoplasm and the nucleus as observed with fluorescence confocal imaging and western blot of nuclear and cytoplasmic fractions (Fig 4, S5 Fig, S2 Mov). A diffuse localization was observed, rather than a punctate pattern like full length NBEA. To assess if the predicted NLS in the DUF1088 domain is important for nuclear localization, the five present arginines (R) were mutated into alanines (A) (Fig 4A). Mutating this sequence resulted in a significant decrease of the confocal nuclear intensity (*P* = 0.047) (Fig 4B, S5 Fig, S3 Mov), demonstrating the importance of the NLS sequence for the nuclear localization of NBEA. A decrease of the nuclear intensity in nuclear and cytoplasmic fractions on western blot could be confirmed, but was not significant due to high variability of the overexpression levels between experiments (*P* = 0.063, Wilcoxon one-tailed signed rank test) (Fig 4C).

### Neurobeachin affects Notch-mediated transcription in N2a cells

**Fig 5:**
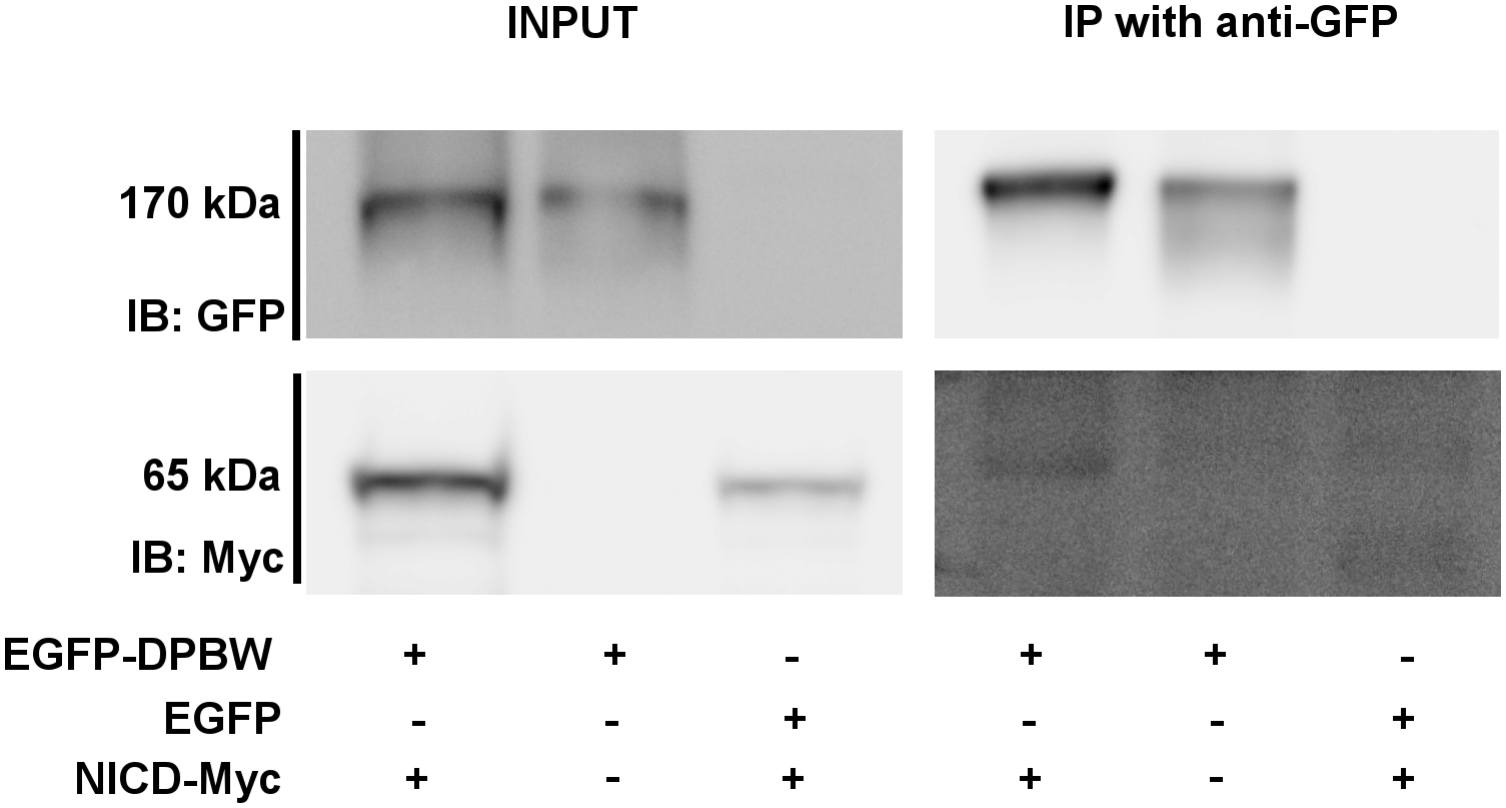
Overexpressed NICD interacts with EGFP-DPBW in HEK293T cells. NICD-Myc co-precipitated with EGFP-DPBW in an immunoprecipitation (IP) using an anti-GFP antibody in HEK293T cells (n = 2). The input for the IP is shown on the left. An immunoblot (IB) for GFP shows EGFP-DPBW at the expected molecular weight of 170 kDa. The IB for Myc shows a signal for NICD-Myc at the expected molecular weight of 65 kDa. IP of EGFP did not lead to a co-precipitation of NICD-Myc, supporting a specific interaction between EGFP-DPBW fusion protein and NICD-Myc. kDa = kiloDalton.

Previously, *Notch* has been identified as a genetic interactor of the *Nbea* homologs *rugose* and *SEL-2*. In the Y2H screen the NOTCH1 intracellular domain (NICD) [Genbank: 224967065] (His2223-Leu2457) was identified to interact with the PBW domain module. This interaction was confirmed by co-precipitating overexpressed NICD-myc with EGFP-DPBW (Fig 5). Upon activation, NOTCH1 is cleaved and its NICD translocates to the nucleus to initiate transcription. Therefore, colocalization with NICD and the effect of NBEA expression on downstream NOTCH mediated transcription were investigated.

**Fig 6:**
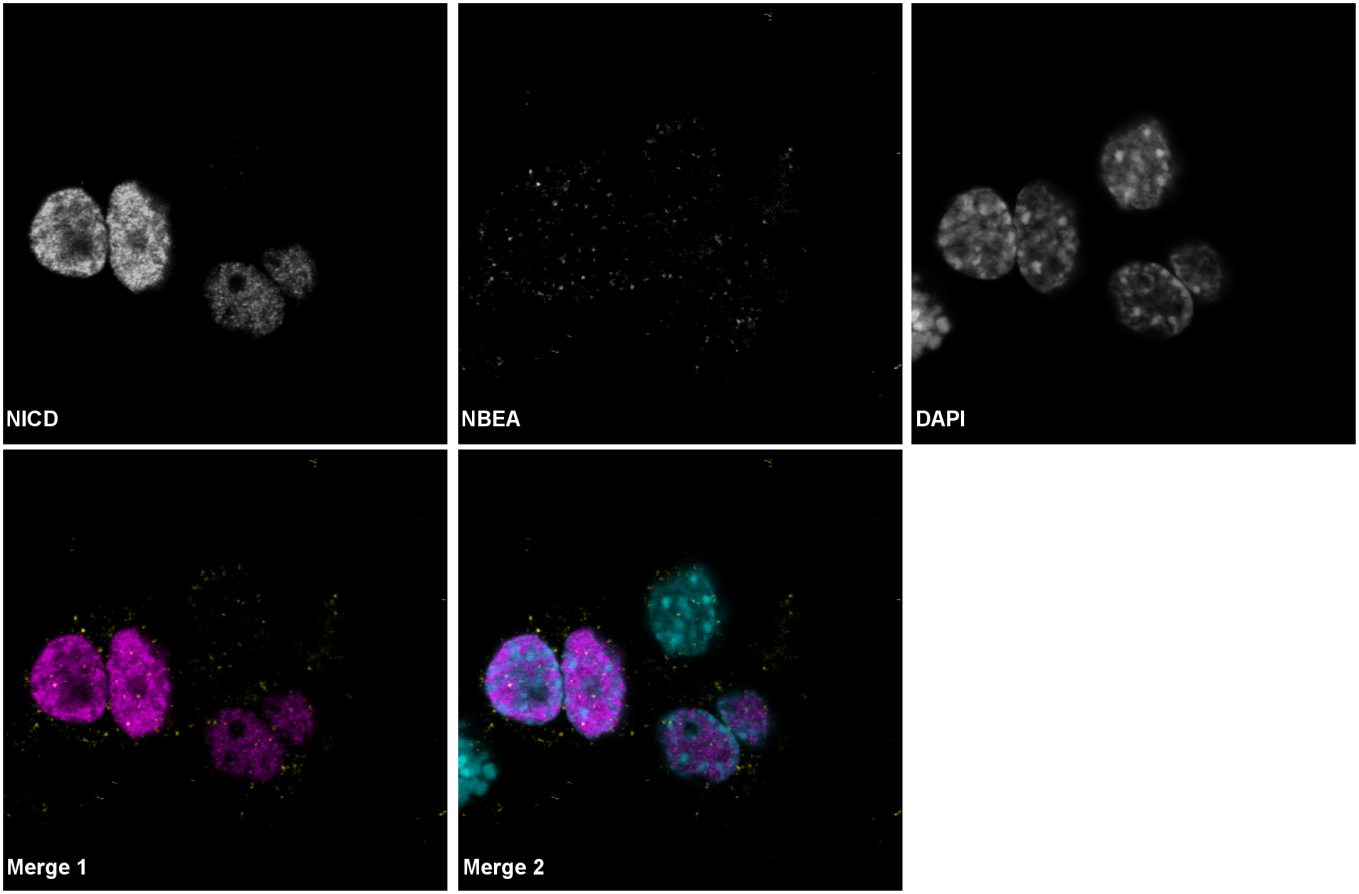
Overexpressed NICD partially co-localizes with NBEA in N2a cells. Confocal image of endogenous NBEA (yellow) and overexpressed NICD (magenta) in N2a cells. DAPI (cyan) was used to stain the nucleus. The separate images are shown in greyscale.

Overexpressed NICD will immediately translocate to the nucleus and only colocalized with the nuclear fraction of endogenous NBEA (Fig 6, S6 Fig, S4 Mov). However, this colocalization was not mutually exclusive, since the NICD nuclear localization was not punctate, but homogenous throughout the nucleus.

**Fig 7:**
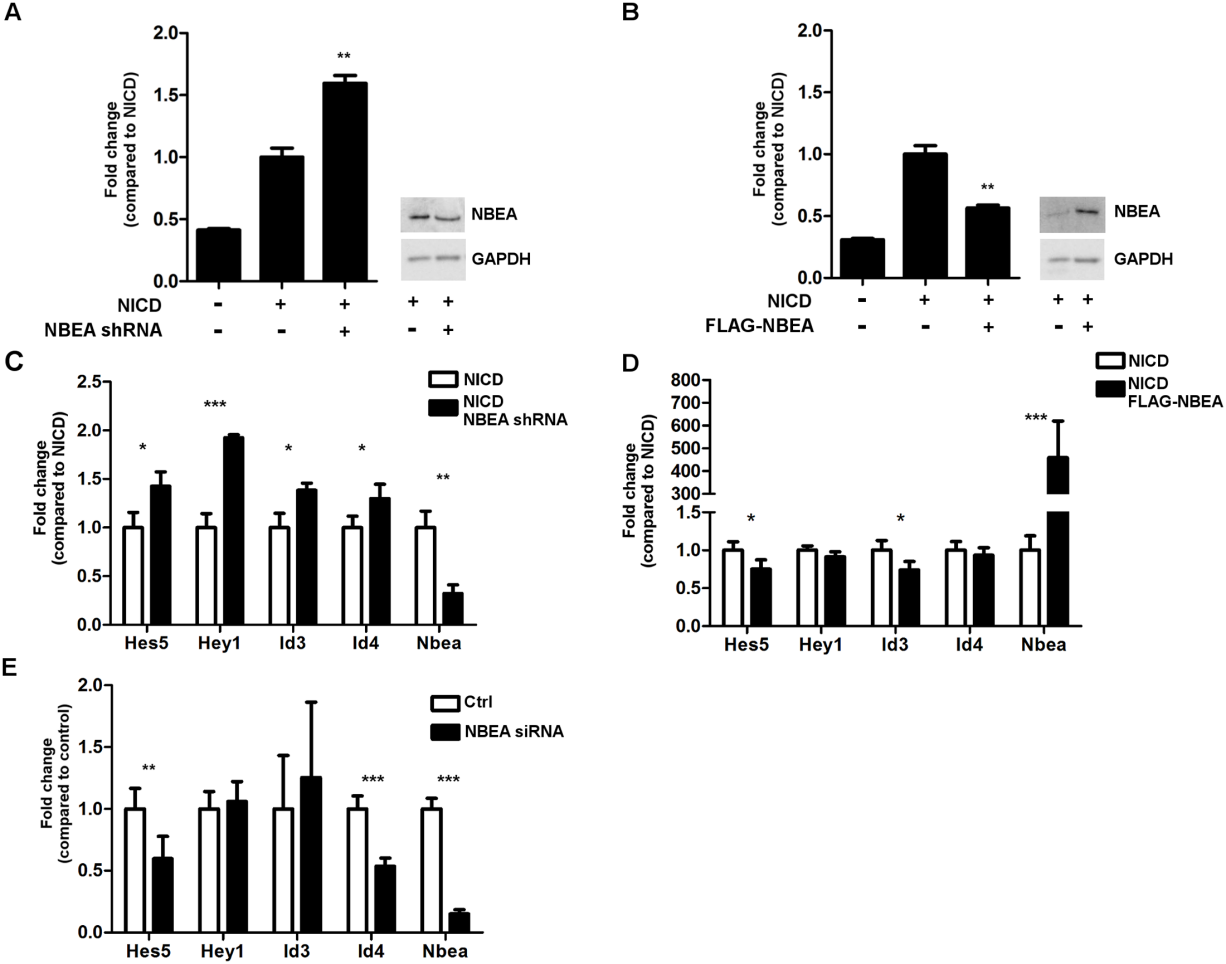
Notch-mediated transcription is affected by NBEA knockdown or overexpression. **A,B)** NICD activity was measured with a luciferase reporter assay. The signal of the 4xwtCBF1 firefly luciferase reporter was measured and normalized to the renilla luciferase activity to control for transfection efficiency. The fold change of the normalized luciferase signal is shown compared to N2a cells with NICD stimulation (NICD) and without altered NBEA expression. N = 3/condition. **A)** N2a cells with or without NBEA KD (NBEA shRNA) were stimulated with overexpression of NICD. A representative western blot shows the 0.7-fold ± 0.1 KD that is obtained for NBEA (*P* = 7E-6). Equal loading of the western blot is shown by GAPDH. **B)** N2a cells with or without NBEA overexpression (FLAG-NBEA) were stimulated with overexpression of NICD. A representative western blot shows the 2.6-fold ± 0.5 overexpression that is obtained for NBEA (*P* = 0.001). Equal loading of the western blot is shown by GAPDH. **C, D, E)** qRT-PCR results are shown for *Nbea* and for four Notch target genes: *Hes5*, *Hey1*, *Id3* and *Id4*. The fold change is shown relative to the condition with normal NBEA expression levels. **C)** The results for N2a cells with NICD stimulation together with or without knockdown of NBEA (NBEA shRNA) are shown. **D)** The results for N2a cells with NICD stimulation together with or without overexpression of NBEA (FLAG-NBEA) are shown. **E)** The results for N2a cells without NICD stimulation together with or without knockdown of NBEA (NBEA siRNA) are shown.

After nuclear translocation, the NICD forms a transcription activating complex with C-promotor binding factor 1 (CBF1) and Mastermind. Therefore, the previously described 4xwtCBF1 luciferase reporter was used to measure NOTCH activity. Overexpressing NICD-myc together with either overexpression (2.6-fold ± 0.5) or knockdown (0.7-fold ± 0.1; shRNA) of NBEA, resulted in a 50% decrease (*P* = 0.004) or increase (*P* = 0.003) in the Notch-mediated transcription of the luciferase reporter, respectively (Fig 7A and 7B).

To validate the results obtained with the luciferase reporter assay, we performed qRT-PCR to assess the expression levels of NOTCH transcriptional targets, namely *Hes5*, *Hey1*, *Id3* and *Id4*. Combining NICD overexpression with knockdown of NBEA (0.3-fold ± 0.1; shRNA) indeed resulted in a significantly increased expression of all the addressed NOTCH targets (*Hey1*: *P* = 0.003; *Hes5*: *P* = 0.02; *Id3*: *P* = 0.02; *Id4*: *P* = 0.04) (Fig 7C), while NBEA overexpression (459-fold ± 161) resulted in a significantly decreased NOTCH-mediated transcription of *Hes5* (*P* = 0.04) and *Id3* (*P* = 0.04). For *Hey1* and *Id4* the differences were not significant (Fig 7D).

Since the Y2H screen for the PBW domain module also picked up HES5 and HEY1 as potential physical interactors, we assessed if knockdown of NBEA could also affect the transcription levels of NOTCH target genes independent of NICD overexpression. Knockdown of NBEA (0.15-fold ± 0.03; siRNA) resulted in decreased expression of *Hes5* (*P* = 0.005) and *Id4* (*P* = 7E-6), while no significant differences in expression could be observed for *Hey1* and *Id3* (Fig 7E). This means that the transcriptional repression of NOTCH targets by NBEA are mediated by NICD. Without NICD no (*Hey1* and *Id3*) or even an opposite effect (*Hes5* and *Id4*) is obtained.

## Discussion

In this study we conducted Y2H screens for the ACA and PBW domain modules of NBEA. PBW interacted mostly with nuclear transcription regulators, while the ACA interactors were enriched in PKA substrates. Furthermore, NBEA was detected in the nucleus in addition to the cytoplasm. A classical nuclear localization signal in the DUF1088 domain was identified and validated. These data suggest a role for NBEA in regulating transcription, besides the already established function in regulating phosphorylation of PKA substrates.

In the Y2H screen, one of the interacting transcription regulators was NOTCH1, and more specifically the NICD. NICD could also be co-precipitated with overexpressed EGFP-DPBW, confirming the physical interaction detected with the Y2H screen. Overexpressing or knocking down NBEA together with NICD showed that NBEA is a negative regulator of NOTCH-mediated transcription.

Based on the Y2H screen, the N-terminal ACA domain module seemed to interact with several PKA substrates, of which two were ASD-implicated genes. This supports the role of NBEA as an AKAP, although, for these proteins, no effect on phosphorylation was observed after knockdown (data not shown). Many of the NBEA domain interactors were previously linked to neurological disorders and/or ASD indicating that NBEA may contribute to ASD by means of one of its interaction partners.

The most established ASD gene is BCKDK which already has been shown to be implicated in five unrelated families with patients with ASD or autistic features [41,42]. The observed Y2H interaction with NBEA domains provides further support for being an ASD gene, since disorder-linked proteins are more likely to establish direct protein interactions with each other, rather than be isolated in a network [43].

One caveat in this study is that, except for NICD, the interactions defined with the Y2H screens have not been validated yet with another technique. Furthermore interaction with a conserved NBEA domain, does not ascertain an interaction with full-length NBEA. Nonetheless, the exploratory nature of this paper will provide an informative base for future studies on NBEA molecular functioning.

### Nuclear NBEA may regulate transcription

LRBA, the closest homolog of NBEA, has recently been shown to shuttle to the nucleus in mouse bone marrow cells after stimulation with LPS [44]. This homolog also contains the PBW domain module which is 76% identical to the module of NBEA. Just before the PBW domain module, a domain of unknown function (DUF1088) is conserved between both proteins. This domain includes a classical NLS consisting of a penta-arginine sequence, in NBEA as well as LRBA. By mutating this NLS we showed that the DUF1088 domain is important for the nuclear localization of the DPBW module. Despite several attempts, NLS mutagenesis of full length NBEA was unsuccessful due to its large GC-rich cDNA sequence. Therefore it is still plausible that other signaling might contribute to the nuclear localization of full length NBEA.

On average, 20% of total NBEA protein showed a punctate pattern in the nucleus of N2a cells, suggesting nuclear compartmentalization. NBEA might have a more pronounced nuclear localization after a specific cell stimulus, similar to LRBA. However stimulation of cAMP production, important for PKA signaling, did not increase nuclear localization of NBEA. Overexpression of NBEA in HEK293T cells, which do not express NBEA endogenously, did not result in any nuclear signal. Thus, a more pronounced nuclear expression will not only depend on cell stimulus, but also on cell type and developmental stage. Rugose, the shared homolog for LRBA and NBEA in *D*. *melanogaster*, has also been observed in some nuclei of neuroblasts delaminating from the ectoderm during stage 8–9 of embryogenesis [45]. Information obtained from the Human protein atlas further supports nuclear expression of NBEA in neuroblast cell lines [40].

Since NBEA is a large protein (327kDa), it will have to be actively imported into the nucleus. Active nuclear import can be mediated by three classes of importins and goes through nuclear pores. The Y2H screen for the PBW domain module also picked up the nuclear pore component 153 (NUP153) as a possible physical interactor, which is important for Importin-β mediated import [46]. Further studies will have to point out how NBEA is imported into the nucleus.

Recently, a second BEACH family member has been found to be implicated in ASD, namely WDFY3. WDFY3 also contains a PBW domain module and a CALL domain, but with very low sequence identity to NBEA. WDFY3 resides mainly in the nucleus, but does not contain a DUF1088 domain. Its function in the nucleus is unknown, but WDFY3 was still categorized into the transcription regulation network implicated in ASD pathogenesis [23,47,48]. Loss of WDFY3 in mice leads to increased proliferation of radial glia cells, increasing the brain cortical size [49].

Studies documenting networks important in ASD pathogenesis mostly pick up protein networks implicated in synaptogenesis and neural growth. But recently proteins involved in chromatin remodeling and transcription regulation have shown to be enriched protein networks for ASD as well [23,50]. Since, based on the Y2H screen, PBW interacted with a number of transcription regulators and NBEA is partially distributed in the nucleus, the effect of NBEA on transcription warrants further research.

### NBEA is a negative regulator of Notch signaling

Here we provide support that NOTCH1 and NBEA can physically interact with each other and that NBEA expression levels can even affect NOTCH-mediated transcription. Studies in *D*. *melanogaster* and *C*. *elegans* have already suggested genetic interactions between the NOTCH pathway and the *Nbea* homologs, rugose and SEL-2, respectively [16,18,19]. Since in *Drosophila* Rg expression is not regulated by NOTCH-mediated transcription, interactions at the protein level with components of NOTCH signaling were suggested [16]. The effect of NBEA levels on NOTCH-mediated transcription is downstream of the NOTCH receptor activation, followed by its internal and external cleavage. Once NOTCH1 is cleaved, the NICD almost immediately translocates to the nucleus to initiate transcription. Therefore, there is a high probability that NBEA will interact with NICD mainly in the nucleus. Alternatively, NBEA overexpression may interfere with NICD nuclear translocation, for instance because they might use the same nuclear transport mechanism, namely Importin-β [51]. Interestingly, Yatim *et al* co-immunoprecipitated NBEA from nuclear extracts of a T-ALL cell line using NICD as bait [52]. These data confirm both the nuclear localization and the potential physical interaction of NBEA with NICD, as observed in our study.

Assessing the effect of NBEA expression on the downstream NOTCH-mediated transcription with the generalized CBF1-dependent luciferase assay, suggested for NBEA to be a negative regulator. There was an opposite effect between overexpression and knockdown of NBEA. This effect could be reproduced when studying specific NOTCH target genes with qRT-PCR, but with a somewhat variable outcome. The effect of NBEA overexpression on specific NOTCH target genes is less pronounced, compared to the effect on expression following NBEA knockdown. It can be expected that a more straightforward result is generated with the luciferase assay, since this is a general assay based on a luciferase plasmid, which is not endogenously expressed. In contrast, the NOTCH specific target genes are endogenously present in N2a cells and might be endogenously regulated at a more complex level, compared to the luciferase-construct. The complete composition of the NOTCH co-activator transcription complex is still not fully characterized and some negative regulators of NOTCH-mediated transcription have already been described. Furthermore, these target genes can be regulated at the level of epi-genetics, for example the methylation status of their promoter. A regional difference in the latter case could explain why not all NOTCH target genes behave in exactly the same way following altered NBEA levels.

Since NICD most likely physically interacts with NBEA, NBEA might interfere with the formation of the co-activating transcription complex of NICD. However, since NICD can also be regulated *post*-translationally and NBEA is an AKAP, NBEA could negatively regulate NOTCH-mediated transcription by affecting the phosphorylation status of NICD.

Although only canonical NOTCH target genes were assessed in this paper, it should be noted that non-canonical pathways could also be affected and contribute to a neuronal phenotype.

Until now, no ASD patients have been described with mutations in *Notch* genes, which may be because NOTCH is implicated in many biological processes during development and will cause more severe developmental disorders. *Notch1* knockout mice already die during embryogenesis [53]. Mutations in human *NOTCH1* can cause aortic valve disease and T-cell acute lymphoblastic leukemia [54]. However regulators of the NOTCH pathway that are highly expressed in the brain, like NBEA, might lead to less severe and tissue-specific symptoms that may be important for ASD etiology.

Recently, an integrated network analysis of *de novo* mutations in ASD patients has highlighted the NOTCH pathway as an important, enriched network. This network seemed to be common for ASD patients with and without intellectual disability (ID), while a network related to synaptic plasticity was only specific for ASD patients with ID [55]. In addition, three gene expression profiling studies of cerebellar or blood samples from ASD patients picked up an enrichment of the NOTCH pathway compared to controls [56–58]. In the central nervous system the NOTCH pathway is important for learning and memory, neuronal maturation and glial cell fate specification [59,60]. Conditional deletions of *Notch1* in brain regions lead to precocious neuronal differentiation [59]. The NOTCH pathway is also known to be important in the cell fate decision between neurogenesis and gliogenesis. Gliogenesis also happened to be enriched for the Y2H PBW interaction list based on the following proteins: NOTCH1, ASCL1, SOX9 and SOX6. ASCL1, also known as MASH1, can regulate and is regulated by the NOTCH signaling pathway [61]. In contrast to NOTCH1, ASCL1 is a well-known proneural transcription factor and blocks gliogenesis [62]. HES1 and HES5, which are NOTCH1 transcription targets, can both repress ASCL1 expression and inhibit ASCL1 through protein-protein interactions. On the other hand ASCL1 can affect NOTCH signaling in neighboring cells by enhancing the expression of DELTA, a ligand of the NOTCH receptor [61]. SOX9 is a glial-specification transcription factor and has been shown to be a transcription target of NOTCH [63]. SOX6 expression can be regulated by SOX9 and has been shown to be important for cell specification to the glial lineage of oligodendrocytes, but is also essential for the maintenance of neural precursor cells by inhibiting neurogenesis [64,65].

A gene expression profiling study of lymphoblastoid cell-lines derived from ASD patients showed a significantly increased expression of SOX9 [56]. Post-mortem neuropathologic studies of persons with ASD often find a decreased number of GABA-ergic Purkinje neurons in the cerebellum and Bailey *et al*. also reported an increase of cerebellar Bergmann glia [66,67]. In general, a switch of cell-fate from neural to glial cells might contribute to ASD pathogenesis, in which the NOTCH signaling pathway can be important and where NBEA might be a relevant modulator.

### The AKAP NBEA may bind PKA substrates

Based on the Y2H screens, the NBEA ACA domain module interacted with a number of validated PKA substrates (MAP4K1, ACTB, FLNA and GBF1), while the PBW module interacted with one (SOX9). It is known that AKAP-proteins are able to bind PKA substrates themselves, bringing the substrates in closer proximity of PKA. Since two other PKA substrates (CREB and CALPAIN2) have already been shown to have increased phosphorylation *in vivo*, due to NBEA haploinsufficiency, we assessed this for GBF1, ACTB and FLNA (data not shown)[26,68]. In N2a cells ACTB did not seem to be phosphorylated by PKA. GBF1 and FLNA showed equal expression after NBEA knockdown, but also equal PKA-mediated phosphorylation. Previously, we have demonstrated that haploinsufficiency of NBEA in mouse platelets affected a significant proportion of the PKA-related phosphoproteome [68]. Knockdown of NBEA in N2A cells did not produce such a pronounced effect (data not shown), suggesting that the chosen experimental settings may not be conductive to study the AKAP function of NBEA. However, it remains tempting to speculate that the ACA domain module plays a role in substrate presentation to PKA.

### NBEA, neurotransmitter receptor trafficking and the cytoskeleton

The cytoskeleton was an enriched GO term, when analyzing possible interactors of the ACA domain module. This is in line with the suggestion that the Concanavalin A-like lectin domain is important for intracellular sorting. In addition this GO enrichment can be of interest for two reasons. Firstly, it has been suggested that NBEA might affect synaptic architecture through modulating the actin cytoskeleton [69]. Here we detected a direct interaction between the NBEA ACA domain module and β-ACTIN (ACTB). Secondly, BEACH proteins are suggested to be involved in (vesicle) trafficking, in which cytoskeletal proteins play an important part. NBEA already has been shown to affect postsynaptic trafficking of neurotransmitter receptors [12]. It has been described to bind to Glycine receptor β-subunit and Synapse associated protein 2 (SAP102), which is implicated in glutamate receptor trafficking. Knockout of NBEA resulted in reduced surface levels of glutamate and Gamma-aminobutyric acid A receptors (GABA_A_R) [24,70]. Recent work found that the PKA-binding ability of NBEA can influence GABA_A_R trafficking, but is not essential [71]. Here we discovered a possible interaction between the NBEA ACA domain module and the GABA_A_R-associated protein (GABARAP). GABARAP regulates clustering of the GABA_A_R at the plasma membrane and can bind directly to microtubuli and indirectly to the actin cytoskeleton [72,73]. Our findings suggest that NBEA might regulate the trafficking of the GABA_A_R through binding with GABARAP and other cytoskeletal proteins, like ACTB and the microtubule network.

Previously a co-immunoprecipitation with full length NBEA in mouse adult tissue identified two tubulins, namely TUBB4A and TUBA4, as possible interactors [24]. Our Y2H analysis also picked up a tubulin, namely TUBA1A, further substantiating an important role for NBEA to be involved in the cytoskeleton.

### The AKAP NBEA may bind other phosphatases and kinases

Based on the Y2H screens, CTTNBP2NL was the only common interactor of the ACA and the PBW domain module. Interestingly, CTTNBP2NL also interacts with two other previously described NBEA interactors, namely Striatin (STRN) and Zinedin (STRN4), forming the striatin-interacting phosphatase and kinase complex (STRIPAK) [24,74,75]. The STRIPAK complex has been described to be implicated in vesicle trafficking, dendritic spine formation and cell migration [75]. Although these specific interactions of NBEA with CTTNBP2BL, STRN and STRN4 have not been validated yet, the fact that they form a protein complex with each other and all have been picked up separately strengthens the possibility that these will be genuine interactors.

PP2A, present in the core of the STRIPAK complex, consists of three functional subunits, the structural subunit A, the catalytic subunit C and the regulatory or scaffolding subunit B. The subunit B can further be divided into three subgroups: B, B’ and B” [76]. The striatin family has been suggested to belong to a new B”‘ group, but this is still controversial [76,77]. In the Y2H screens the ACA domain did interact with other structural subunits of PP2A (PPP2R5E (B’ subunit) and PPP2R3C (B” subunit)), but we did not find any member of the striatin family to be interacting with a NBEA domain module. This raises the hypothesis that NBEA is able to interact with the serine/threonine (S/T) kinase PKA, but also with the S/T phosphatase PP2A.

A-kinase anchoring proteins (AKAPs) can form multi-protein complexes binding to other phosphatases and kinases as well [27]. In this way there can be a tight regulation of S/T phosphorylation and other posttranslational signaling in a specific cell compartment. In addition, other interacting kinases we picked up with the Y2H screens were BCKDK, PKM, MAP4K1, CDK4 and MAP2K2. NBEA might be an important scaffold for the formation of many different signaling complexes.

## Conclusions

In this study we have provided the first evidence for nuclear localization of NBEA and identified a NLS. The possible interaction of the PBW domain module with a number of transcription factors suggests a role in regulating transcription. This was further substantiated with the functional interaction with NOTCH1, which has previously been shown to be a genetic interactor in *D*. *melanogaster* and *C*. *Elegans* model systems. Our data also suggest that the ACA domain module may play a role in PKA substrate presentation. Taken together, this study provides experimental evidence to the multifaceted role of NBEA in signaling networks relevant for ASD.

## Supporting Information

S1 Appendix Nuclear localization signal prediction results obtained with NucPred. A NucPred score is given for the protein sequence of NBEA. The NBEA amino acid sequence is shown combined with color coding for positively and negatively influencing subsequences for nuclear localization. The color coding legend is shown at the bottom of the sequence. A red framework highlights the penta-arginine sequence in the DUF1088 domain that is predicted to be a nuclear localization signal.
(DOCX)

S2 Appendix Nuclear export signal prediction results obtained with NetNES 1.1. The bio-informatics results for leucine-rich NES prediction in the NBEA protein obtained with NetNES 1.1. Each amino acid of NBEA has a NES-score. If a NES is predicted for an amino acid within a certain sequence a ‘y’ for yes is shown in the ‘Predicted’ column. A prediction for a NES is made when the NES-score is higher than a predefined threshold within the tool. The NES scores for each amino acid compared to the threshold level are visualized in a graph.
(DOCX)

S1 Fig Specificity of the homemade antibody for endogenous NBEA. **A)** HEK293T cells, which do not express NBEA endogenously, were transfected with the pcDNA3.1-FLAG-Nbea construct. Confocal images show an exclusive colocalization between Flag (yellow) and NBEA (magenta) for cells that overexpress FLAG-NBEA. Untransfected HEK293T cells do not show any non-specific immunostaining for NBEA. Nuclei were stained with DAPI (cyan). Separate confocal images are shown in greyscale. A 2D Z-projection of the confocal Z-stack is shown as well (Z-project). **B)** The NBEA antibody detects overexpressed FLAG-NBEA at the expected molecular weight of 327 kDa in HEK293T cell lysate (lane 2) compared to untransfected HEK293T cells (lane 1). GAPDH is shown for normalization. **C)** The protein of 327 kDa is endogenously detected by the home-made antibody in N2a cell lysates (lane 1 and 3). This is NBEA-specific, since this fragment cannot be detected anymore in N2a cell lysates transfected with *Nbea*-specific shRNA (lane 1 and 3). GAPDH is shown for normalization.
(TIF)

S2 Fig A 2D Z-projection of endogenous NBEA in N2a cells. A 2D Z-projection of endogenous NBEA (yellow) in N2a cells is shown. DAPI (cyan) was used to stain the nucleus. The separate images are shown in greyscale.
(TIF)

S3 Fig Nuclear localization of endogenous NBEA compared to background control. **A)** Confocal image of endogenous NBEA (yellow) in N2a cells. DAPI (cyan) was used to stain the nucleus. The separate images are shown in greyscale. Scale (white bar) is 10 μm. **B)** Confocal image as a control for background staining, where no primary antibody, but the same secondary antibody (yellow) and confocal settings were used. DAPI (Cyan) was used to stain the nucleus. The separate images are shown in greyscale. Scale (white bar) is 10 μm.
(TIF)

S4 Fig Human protein atlas data for endogenous NBEA. **A)** Confocal image obtained from the Human protein atlas database (www.proteinatlas.org) of SH-SY5Y, human neuroblast cells for endogenous NBEA (green) immunostaining. DAPI (blue) was used to stain the nucleus. Another antibody was used for NBEA immunostaining than is used in this article, namely HPA040385. **B)** A larger magnification is shown from the area in the merged panel A, which is marked with a dashed, white line.
(TIF)

S5 Fig A 2D Z-projection of the EGFP-DPBW fusion protein without or with the NLS mutation. **A)** A 2D Z-projection of the EGFP-DPBW fusion protein (yellow) in N2a cells is shown. DAPI (cyan) was used to stain the nucleus. The separate images are shown in greyscale. **B)** A 2D Z-projection of the EGFP-DPBW^MUT^ fusion protein (yellow) in N2a cells is shown. DAPI (cyan) was used to stain the nucleus. The separate images are shown in greyscale.
(TIF)

S6 Fig A 2D Z-projection of overexpressed NICD and endogenous NBEA in N2a cells. A 2D Z-projection of endogenous NBEA (yellow) and overexpressed NICD (magenta) in N2a cells is shown. DAPI (cyan) was used to stain the nucleus. The separate images are shown in greyscale.
(TIF)

S1 Mov Z-stack of endogenous NBEA in N2a cells. A Z-stack of confocal images for endogenous NBEA (yellow) in N2a cells is shown. DAPI (cyan) was used to stain the nucleus.
(AVI)

S2 Mov Z-stack of the EGFP-DPBW fusion proteins in N2a cells. A Z-stack of confocal images for the EGFP-DPBW fusion protein (yellow) in N2a cells is shown. DAPI (cyan) was used to stain the nucleus.
(AVI)

S3 Mov Z-stack of the EGFP-DPBW^MUT^ fusion protein in N2a cells. A Z-stack of confocal images for the EGFP-DPBW^MUT^ fusion protein (yellow) in N2a cells is shown. DAPI (cyan) was used to stain the nucleus.
(AVI)

S4 Mov Z-stack of overexpressed NICD and endogenous NBEA in N2a cells. A Z-stack of confocal images for endogenous NBEA (yellow) and overexpressed NICD (magenta) in N2a cells is shown.
(AVI)

S1 Table Targeted sequences of *Nbea* with siGENOME Mouse Nbea (26422) siRNA. The targeted sequences of *Nbea* are shown coupled to the antisense sequences of siGENOME Mouse Nbea (26422) siRNA SMARTpool (GE Healthcare, UK). AA = amino acid.
(XLSX)

S2 Table Primers per gene used for qRT-PCR and for site-directed mutagenesis PCR of pEGFP-DPBW. The sequences of the primers are presented in a 5’ to 3’ orientation. Nbea: Neurobeachin; Ppia: peptidylprolyl isomerase A; Gadd45a: growth arrest and DNA-damage-inducible alpha; Hes5: hairy and enhancer of split 5 (Drosophila); Hey1: hairy/enhancer-of-split related with YRPW motif 1; Id3: inhibitor of DNA binding 3; Id4: inhibitor of DNA binding 4.
(XLSX)

S3 Table Y2H interaction partners for the NBEA ACA domain module. ^a^ Number of independent colonies or hits compared to the total number of hits for the specific interaction partner.
^b^ Pubmed ID (PMID) of articles linking between the gene and ASD.
^c^ Known PKA substrates according to www.phosphosite.org. *y = yes*
^d^ Other neurological disorders linked to the gene with the corresponding phenotype MIM number (OMIM) or PMID.
(XLSX)

S4 Table Y2H interaction partners for the NBEA PBW domain module. ^a^ Number of independent colonies or hits compared to the total number of hits for the specific interaction partner.
^b^ Pubmed ID (PMID) of articles linking between the gene and ASD.
^c^ Known PKA substrates according to www.phosphosite.org. *y = yes*
^d^ Other neurological disorders linked to the gene with the corresponding phenotype MIM number (OMIM) or PMID.
(XLSX)

S5 Table GO terms showing significant overrepresentation in proteins interacting with the PBW domain module. Complete results from BiNGO analysis within Cytoscape of the protein list interacting with the NBEA PBW domain module. Each tab shows one of the three GO categories: molecular function, cellular component or biological process. *GO-ID*: *Gene Ontology identification*, *x*: *number of interacting proteins with the specific GO-ID*. *X*: *total number of interacting proteins with any GO-ID within the specific GO category*. *n*: *number of proteins in the whole mouse genome with the specific GO-ID*. *N*: *total number of proteins in the whole mouse genome with any GO-ID within the specific GO category*.
(XLS)

S6 Table GO terms showing significant overrepresentation in proteins interacting with the ACA domain module. Complete results from BiNGO analysis within cytoscape of the protein list interacting with the NBEA ACA domain module. Each tab shows one of the three GO categories: molecular function, cellular component or biological process. *GO-ID*: *Gene Ontology identification*, *x*: *number of interacting proteins with the specific GO-ID*. *X*: *total number of interacting proteins with any GO-ID within the specific GO category*. *n*: *number of proteins in the whole mouse genome with the specific GO-ID*. *N*: *total number of proteins in the whole mouse genome with any GO-ID within the specific GO category*.
(XLS)
