# Supplementary material for: Nuclear Localization of the Autism Candidate Gene Neurobeachin and Functional Interaction with the NOTCH1 Intracellular Domain Indicate a Role in Regulating Transcription
Source: PLoS One. 2016 Mar 21;11(3):e0151954. doi: 10.1371/journal.pone.0151954 (PMC4801420; doi:10.1371/journal.pone.0151954)
Supplement: S1 Appendix — A NucPred score is given for the protein sequence of NBEA. The NBEA amino acid sequence is shown combined with color coding for positively and negatively influencing subsequences for nuclear localization. The color coding legend is shown at the bottom of the sequence. A red framework highlights the penta-arginine sequence in the DUF1088 domain that is predicted to be a nuclear localization signal. (DOCX) [file pone.0151954.s001.docx]

**NucPred**

The NucPred score for your sequence is 0.56 (see [score help](http://www.sbc.su.se/%7Emaccallr/nucpred/cgi-bin/single.cgi#scorehelp) below)

**1 MASDKPGPGLEPQPVALLAVGAGGGAGGGGAMGEPRGAAGSGPVVLPAGM 50
 51 INPSVPIRNIRMKFAVLIGLIQVGEVSNRDIVETVLNLLVGGEFDLEMNF 100
 101 IIQDAESITCMTELLEHCDVTCQAEIWSMFTAILRKSVRNLQTSTEVGLI 150
 151 EQVLLKMSAVDDMIADLLVDMLGVLASYSITVKELKLLFSMLRGESGIWP 200
 201 RHAVKLLSVLNQMPQRHGPDTFFNFPGCSAAAIALPPIAKWPYQNGFTLN 250
 251 TWFRMDPLNNINVDKDKPYLYCFRTSKGVGYSAHFVGNCLIVTSLKSKGK 300
 301 GFQHCVKYDFQPRKWYMISIVHIYNRWRNSEIRCYVNGQLVSYGDMAWHV 350
 351 NTNDSYDKCFLGSSETADANRVFCGQLGAVYVFSEALNPAQIFAVHQLGP 400
 401 GYKSTFKFKSESDIHLAEHHKQVLYDGKLASSIAFSYNAKATDAQLCLES 450
 451 SPKENASIFVHSPHALMLQDVKAIVTHSIHSAIHSIGGIQVLFPLFAQLD 500
 501 NRQLNDSQVETTVCATLLAFLVELLKSSVAMQEQMLGGKGFLVIGYLLEK 550
 551 SSRVHITRAVLEQFLSFAKYLDGLSHGAPLLKQLCDHILFNPAIWIHTPA 600
 601 KVQLSLYTYLSAEFIGTATIYTTIRRVGTVLQLMHTLKYYYWVINPADSS 650
 651 GIAPKGLDGPRPSQKEIISLRAFMLLFLKQLILKDRGVKEDELQSILNYL 700
 701 LTMHEDENIHDVLQLLVALMSEHPASMIPAFDQRNGIRVIYKLLASKSES 750
 751 IWVQALKVLGYFLKHLGHKRKVEIMHTHSLFTLLGERLMLHTNTVTVTTY 800
 801 NTLYEILTEQVCTQVVHKPHPEPDSTVKIQNPMILKVVATLLKNSTPSAE 850
 851 LMEVRRLFLSDMIKLFSNSRENRRCLLQCSVWQDWMFSLGYINPKSSEEQ 900
 901 KITEMVYNIFRILLYHAIKYEWGGWRVWVDTLSIAHSKVTYEAHKEYLAK 950
 951 MYEEYQRQEEENIKKGKKGNVSTISGLSSQTAGAKGGMEIREIEDLSQSQ 1000
1001 SPESETDYPVSTDTRDLLMSTKVSDDILGSSDRPGSGVHVEVHDLLVDIK 1050
1051 AEKVEATEVKLDDMDLSPETLVGGENGALVEVESLLDNVYSAAVEKLQNN 1100
1101 VHGSVGIIKKNEEKDNGPLITLADEKEELPNSSTPFLFDKIPRQEEKLLP 1150
1151 ELSSNHIIPNIQDTQVHLGVSDDLGLLAHMTASVELTCTSSIMEEKDFRI 1200
1201 HTTSDGVSSVSERELASSTKGLDYAEMTATTLETESSNSKAVPNVDAGSI 1250
1251 ISDTERSDDGKESGKEIRKIQTTATTQAVQGRSSTQQDRDLRVDLGFRGM 1300
1301 PMTEEQRRQFSPGPRTTMFRIPEFKWSPMHQRLLTDLLFALETDVHVWRS 1350
1351 HSTKSVMDFVNSNENIIFVHNTIHLISQMVDNIIIACGGILPLLSAATSP 1400
1401 TGSKTELENIEVTQGMSAETAVTFLSRLMAMVDVLVFASSLNFSEIEAEK 1450
1451 NMSSGGLMRQCLRLVCCVAVRNCLECRQRQRDRGSKSSHGSSKPQEAPHS 1500
1501 VTAASASKTPLENVPGNLSPIKDPDRLLQDVDINRLRAVVFRDVDDSKQA 1550
1551 QFLALAVVYFISVLMVSKYRDILEPQRETARTGSQPGRNIRQEINSPTST 1600
1601 VVVIPSIPHPSLNHGLLAKLMPEQSFAHSFYKETPATFPDTVKEKETPTP 1650
1651 GEDIQLESSVPHTDSGMGEEQVASILDGAELEPAAGPDAMSELLSTLSSE 1700
1701 VKKSQESLTEHPSEMLKPAPSISSISQTKGINVKEILKSLVAAPVEIAEC 1750
1751 GPEPIPYPDPALKREAHAILPMQFHSFDRSVVVPVKKPPPGSLAVTTVGA 1800
1801 TAAGSGLPTGSTSSIFAAPGATPKSMINTTGAVDSGSSSSSSSSSFVNGA 1850
1851 TSKNLPAVQTVAPMPEDSAENMSITAKLERALEKVAPLLREIFVDFAPFL 1900
1901 SRTLLGSHGQELLIEGLVCMKSSTSVVELVMLLCSQEWQNSIQKNAGLAF 1950
1951 IELINEGRLLCHAMKDHIVRVANEAEFILNRQRAEDVHKHAEFESQCAQY 2000
2001 AADRREEEKMCDHLISAAKHRDHVTANQLKQKILNILTNKHGAWGAVSHS 2050
2051 QLHDFWRLDYWEDDLRRRRRFVRNAFGSTHAEALLKSAVEYGTEEDVVKS 2100
2101 KKAFRSQAIVNQNSETELMLEGDDDAVSLLQEKEIDNLAGPVVLSTPAQL 2150
2151 IAPVVVAKGTLSITTTEIYFEVDEDDAAFKKIDTKVLAYTEGLHGKWMFS 2200
2201 EIRAVFSRRYLLQNTALEVFMANRTSVMFNFPDQATVKKVVYSLPRVGVG 2250
2251 TSYGLPQARRISLATPRQLYKSSNMTQRWQRREISNFEYLMFLNTIAGRT 2300
2301 YNDLNQYPVFPWVLTNYESEELDLTLPGNFRDLSKPIGALNPKRAVFYAE 2350
2351 RYETWEEDQSPPFHYNTHYSTATSALSWLVRIEPFTTFFLNANDGKFDHP 2400
2401 DRTFSSIARSWRTSQRDTSDVKELIPEFYYLPEMFVNSNGYHLGVREDEV 2450
2451 VVNDVDLPPWAKKPEDFVRINRMALESEFVSCQLHQWIDLIFGYKQRGPE 2500
2501 AVRALNVFHYLTYEGSVNLDSITDPVLREAMEAQIQNFGQTPSQLLIEPH 2550
2551 PPRSSAMHLCFLPQSPLMFKDQMQQDVIMVLKFPSNSPVTHVAANTLPHL 2600
2601 TIPAVVTVTCSRLFAVNRWHNTVGLRGAPGYSLDQAHHLPIEMDPLIANN 2650
2651 SGVNKRQITDLVDQSIQINAHCFVVTADNRYILICGFWDKSFRVYSTETG 2700
2701 KLTQIVFGHWDVVTCLARSESYIGGDCYIVSGSRDATLLLWYWSGRHHII 2750
2751 GDNPNSSDYPAPRAVLTGHDHEVVCVSVCAELGLVISGAKEGPCLVHTIT 2800
2801 GDLLRALEGPENCLFPRLISVSSEGHCIIYYERGRFSNFSINGKLLAQME 2850
2851 INDSTRAILLSSDGQNLVTGGDNGVVEVWQACDFKQLYIYPGCDAGIRAM 2900
2901 DLSHDQRTLITGMASGSIVAFNIDFNRWHYEHQNRY 2936**

Positively and negatively influencing subsequences are coloured according to the following scale:

(non-nuclear) negative ||||||||||||||||||||||||||||||||||||||||||||||||||||||||||||||||||||||||||||||||||||||||||||||||||||| positive (nuclear)
