# Supplementary material for: Nuclear Localization of the Autism Candidate Gene Neurobeachin and Functional Interaction with the NOTCH1 Intracellular Domain Indicate a Role in Regulating Transcription
Source: PLoS One. 2016 Mar 21;11(3):e0151954. doi: 10.1371/journal.pone.0151954 (PMC4801420; doi:10.1371/journal.pone.0151954)
Supplement: S2 Appendix — The bio-informatics results for leucine-rich NES prediction in the NBEA protein obtained with NetNES 1.1. Each amino acid of NBEA has a NES-score. If a NES is predicted for an amino acid within a certain sequence a ‘y’ for yes is shown in the ‘Predicted’ column. A prediction for a NES is made when the NES-score is higher than a predefined threshold within the tool. The NES scores for each amino acid compared to the threshold level are visualized in a graph. (DOCX) [file pone.0151954.s002.docx]

>Sequence - NetNES 1.1 prediction


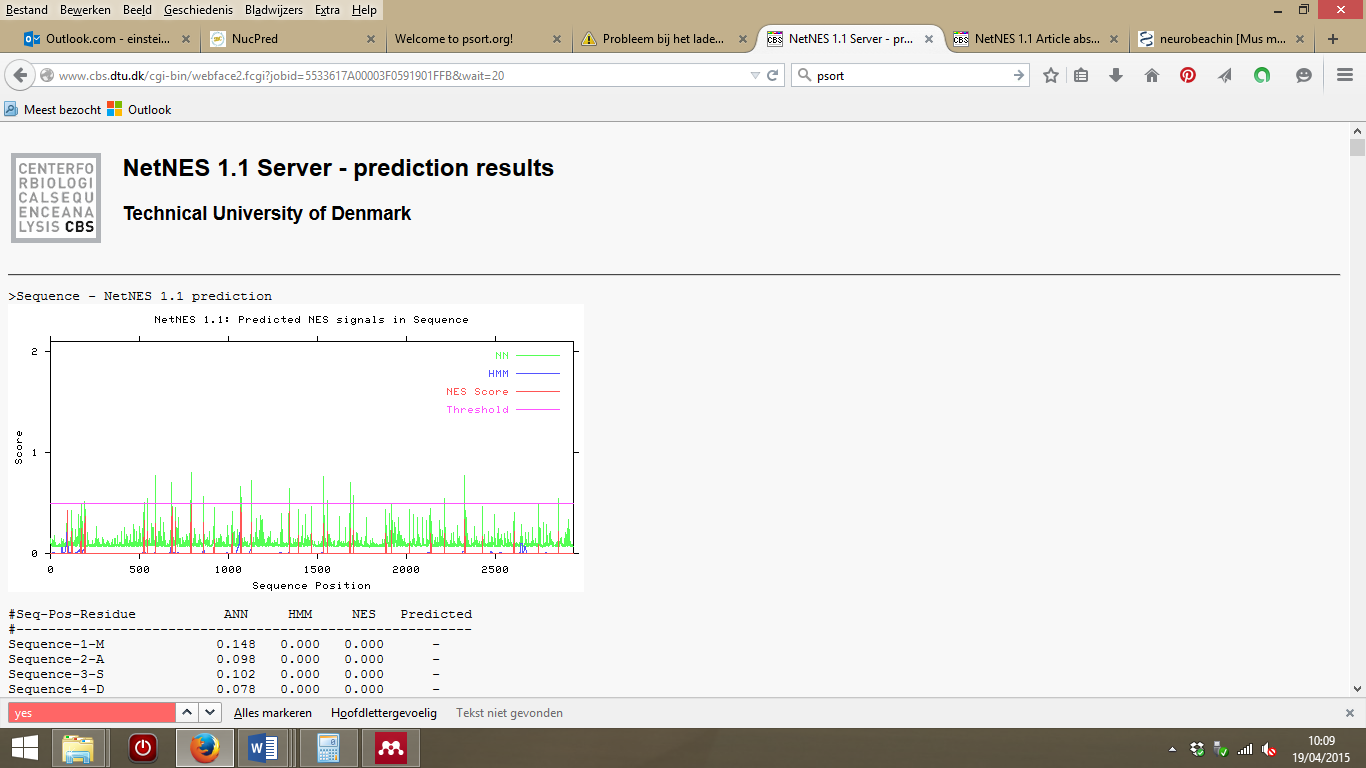


#Seq-Pos-Residue ANN HMM NES Predicted

#---------------------------------------------------------

Sequence-1-M 0.148 0.000 0.000 -

Sequence-2-A 0.098 0.000 0.000 -

Sequence-3-S 0.102 0.000 0.000 -

Sequence-4-D 0.078 0.000 0.000 -

Sequence-5-K 0.101 0.000 0.000 -

Sequence-6-P 0.081 0.000 0.000 -

Sequence-7-G 0.084 0.000 0.000 -

Sequence-8-P 0.076 0.000 0.000 -

Sequence-9-G 0.075 0.000 0.000 -

Sequence-10-L 0.142 0.009 0.000 -

Sequence-11-E 0.074 0.009 0.000 -

Sequence-12-P 0.135 0.009 0.000 -

Sequence-13-Q 0.070 0.009 0.000 -

Sequence-14-P 0.074 0.009 0.000 -

Sequence-15-V 0.090 0.009 0.000 -

Sequence-16-A 0.070 0.009 0.000 -

Sequence-17-L 0.128 0.009 0.000 -

Sequence-18-L 0.080 0.005 0.000 -

Sequence-19-A 0.077 0.005 0.000 -

Sequence-20-V 0.180 0.005 0.000 -

Sequence-21-G 0.070 0.000 0.000 -

Sequence-22-A 0.168 0.000 0.000 -

Sequence-23-G 0.109 0.000 0.000 -

Sequence-24-G 0.068 0.000 0.000 -

Sequence-25-G 0.078 0.000 0.000 -

Sequence-26-A 0.084 0.000 0.000 -

Sequence-27-G 0.106 0.000 0.000 -

Sequence-28-G 0.071 0.000 0.000 -

Sequence-29-G 0.066 0.000 0.000 -

Sequence-30-G 0.067 0.000 0.000 -

Sequence-31-A 0.067 0.000 0.000 -

Sequence-32-M 0.076 0.000 0.000 -

Sequence-33-G 0.070 0.000 0.000 -

Sequence-34-E 0.080 0.000 0.000 -

Sequence-35-P 0.067 0.000 0.000 -

Sequence-36-R 0.069 0.000 0.000 -

Sequence-37-G 0.068 0.000 0.000 -

Sequence-38-A 0.066 0.000 0.000 -

Sequence-39-A 0.070 0.000 0.000 -

Sequence-40-G 0.084 0.000 0.000 -

Sequence-41-S 0.069 0.000 0.000 -

Sequence-42-G 0.069 0.000 0.000 -

Sequence-43-P 0.068 0.000 0.000 -

Sequence-44-V 0.073 0.000 0.000 -

Sequence-45-V 0.073 0.000 0.000 -

Sequence-46-L 0.086 0.000 0.000 -

Sequence-47-P 0.069 0.000 0.000 -

Sequence-48-A 0.074 0.000 0.000 -

Sequence-49-G 0.067 0.000 0.000 -

Sequence-50-M 0.072 0.000 0.000 -

Sequence-51-I 0.103 0.000 0.000 -

Sequence-52-N 0.076 0.000 0.000 -

Sequence-53-P 0.077 0.000 0.000 -

Sequence-54-S 0.071 0.000 0.000 -

Sequence-55-V 0.085 0.004 0.000 -

Sequence-56-P 0.077 0.004 0.000 -

Sequence-57-I 0.078 0.005 0.000 -

Sequence-58-R 0.068 0.004 0.000 -

Sequence-59-N 0.068 0.004 0.000 -

Sequence-60-I 0.094 0.017 0.000 -

Sequence-61-R 0.070 0.017 0.000 -

Sequence-62-M 0.144 0.063 0.000 -

Sequence-63-K 0.073 0.060 0.000 -

Sequence-64-F 0.092 0.060 0.000 -

Sequence-65-A 0.070 0.060 0.000 -

Sequence-66-V 0.087 0.061 0.000 -

Sequence-67-L 0.090 0.059 0.000 -

Sequence-68-I 0.078 0.059 0.000 -

Sequence-69-G 0.096 0.013 0.000 -

Sequence-70-L 0.120 0.014 0.000 -

Sequence-71-I 0.110 0.005 0.000 -

Sequence-72-Q 0.100 0.004 0.000 -

Sequence-73-V 0.134 0.004 0.000 -

Sequence-74-G 0.069 0.001 0.000 -

Sequence-75-E 0.094 0.001 0.000 -

Sequence-76-V 0.104 0.001 0.000 -

Sequence-77-S 0.071 0.001 0.000 -

Sequence-78-N 0.097 0.001 0.000 -

Sequence-79-R 0.079 0.001 0.000 -

Sequence-80-D 0.071 0.001 0.000 -

Sequence-81-I 0.090 0.010 0.000 -

Sequence-82-V 0.068 0.018 0.000 -

Sequence-83-E 0.072 0.018 0.000 -

Sequence-84-T 0.080 0.023 0.000 -

Sequence-85-V 0.099 0.026 0.000 -

Sequence-86-L 0.108 0.072 0.000 -

Sequence-87-N 0.080 0.072 0.000 -

Sequence-88-L 0.298 0.184 0.000 -

Sequence-89-L 0.084 0.199 0.000 -

Sequence-90-V 0.130 0.204 0.000 -

Sequence-91-G 0.195 0.185 0.000 -

Sequence-92-G 0.094 0.185 0.000 -

Sequence-93-E 0.109 0.185 0.000 -

Sequence-94-F 0.157 0.200 0.000 -

Sequence-95-D 0.097 0.200 0.000 -

Sequence-96-L 0.426 0.211 0.426 -

Sequence-97-E 0.082 0.121 0.000 -

Sequence-98-M 0.158 0.121 0.000 -

Sequence-99-N 0.083 0.031 0.000 -

Sequence-100-F 0.081 0.031 0.000 -

Sequence-101-I 0.121 0.024 0.000 -

Sequence-102-I 0.091 0.021 0.000 -

Sequence-103-Q 0.087 0.007 0.000 -

Sequence-104-D 0.078 0.007 0.000 -

Sequence-105-A 0.115 0.007 0.000 -

Sequence-106-E 0.092 0.007 0.000 -

Sequence-107-S 0.090 0.007 0.000 -

Sequence-108-I 0.119 0.008 0.000 -

Sequence-109-T 0.094 0.008 0.000 -

Sequence-110-C 0.099 0.008 0.000 -

Sequence-111-M 0.100 0.008 0.000 -

Sequence-112-T 0.075 0.007 0.000 -

Sequence-113-E 0.093 0.007 0.000 -

Sequence-114-L 0.163 0.007 0.000 -

Sequence-115-L 0.080 0.002 0.000 -

Sequence-116-E 0.110 0.000 0.000 -

Sequence-117-H 0.215 0.000 0.000 -

Sequence-118-C 0.086 0.000 0.000 -

Sequence-119-D 0.156 0.000 0.000 -

Sequence-120-V 0.428 0.000 0.215 -

Sequence-121-T 0.083 0.000 0.000 -

Sequence-122-C 0.158 0.000 0.000 -

Sequence-123-Q 0.090 0.000 0.000 -

Sequence-124-A 0.129 0.000 0.000 -

Sequence-125-E 0.089 0.000 0.000 -

Sequence-126-I 0.096 0.000 0.000 -

Sequence-127-W 0.080 0.000 0.000 -

Sequence-128-S 0.076 0.000 0.000 -

Sequence-129-M 0.112 0.000 0.000 -

Sequence-130-F 0.084 0.000 0.000 -

Sequence-131-T 0.089 0.000 0.000 -

Sequence-132-A 0.084 0.000 0.000 -

Sequence-133-I 0.113 0.000 0.000 -

Sequence-134-L 0.095 0.000 0.000 -

Sequence-135-R 0.097 0.000 0.000 -

Sequence-136-K 0.105 0.000 0.000 -

Sequence-137-S 0.080 0.000 0.000 -

Sequence-138-V 0.187 0.000 0.000 -

Sequence-139-R 0.099 0.000 0.000 -

Sequence-140-N 0.071 0.000 0.000 -

Sequence-141-L 0.150 0.007 0.000 -

Sequence-142-Q 0.087 0.006 0.000 -

Sequence-143-T 0.235 0.006 0.000 -

Sequence-144-S 0.078 0.006 0.000 -

Sequence-145-T 0.068 0.006 0.000 -

Sequence-146-E 0.101 0.006 0.000 -

Sequence-147-V 0.077 0.008 0.000 -

Sequence-148-G 0.077 0.008 0.000 -

Sequence-149-L 0.079 0.013 0.000 -

Sequence-150-I 0.171 0.013 0.000 -

Sequence-151-E 0.101 0.013 0.000 -

Sequence-152-Q 0.076 0.013 0.000 -

Sequence-153-V 0.146 0.014 0.000 -

Sequence-154-L 0.135 0.018 0.000 -

Sequence-155-L 0.156 0.026 0.000 -

Sequence-156-K 0.152 0.023 0.000 -

Sequence-157-M 0.108 0.026 0.000 -

Sequence-158-S 0.102 0.022 0.000 -

Sequence-159-A 0.385 0.022 0.000 -

Sequence-160-V 0.117 0.026 0.000 -

Sequence-161-D 0.073 0.022 0.000 -

Sequence-162-D 0.158 0.022 0.000 -

Sequence-163-M 0.090 0.031 0.000 -

Sequence-164-I 0.146 0.045 0.000 -

Sequence-165-A 0.118 0.045 0.000 -

Sequence-166-D 0.072 0.045 0.000 -

Sequence-167-L 0.133 0.046 0.000 -

Sequence-168-L 0.085 0.043 0.000 -

Sequence-169-V 0.143 0.043 0.000 -

Sequence-170-D 0.079 0.037 0.000 -

Sequence-171-M 0.246 0.037 0.000 -

Sequence-172-L 0.493 0.011 0.247 -

Sequence-173-G 0.250 0.011 0.000 -

Sequence-174-V 0.201 0.011 0.000 -

Sequence-175-L 0.153 0.007 0.000 -

Sequence-176-A 0.233 0.005 0.000 -

Sequence-177-S 0.315 0.005 0.000 -

Sequence-178-Y 0.074 0.005 0.000 -

Sequence-179-S 0.087 0.005 0.000 -

Sequence-180-I 0.160 0.019 0.000 -

Sequence-181-T 0.084 0.020 0.000 -

Sequence-182-V 0.088 0.029 0.000 -

Sequence-183-K 0.070 0.026 0.000 -

Sequence-184-E 0.074 0.026 0.000 -

Sequence-185-L 0.099 0.037 0.000 -

Sequence-186-K 0.069 0.037 0.000 -

Sequence-187-L 0.185 0.037 0.000 -

Sequence-188-L 0.114 0.028 0.000 -

Sequence-189-F 0.187 0.027 0.000 -

Sequence-190-S 0.236 0.017 0.000 -

Sequence-191-M 0.171 0.017 0.000 -

Sequence-192-L 0.518 0.000 0.293 -

Sequence-193-R 0.229 0.000 0.000 -

Sequence-194-G 0.440 0.000 0.367 -

Sequence-195-E 0.108 0.000 0.000 -

Sequence-196-S 0.104 0.000 0.000 -

Sequence-197-G 0.153 0.000 0.000 -

Sequence-198-I 0.109 0.000 0.000 -

Sequence-199-W 0.081 0.000 0.000 -

Sequence-200-P 0.079 0.000 0.000 -

Sequence-201-R 0.074 0.000 0.000 -

Sequence-202-H 0.074 0.000 0.000 -

Sequence-203-A 0.080 0.000 0.000 -

Sequence-204-V 0.073 0.001 0.000 -

Sequence-205-K 0.067 0.001 0.000 -

Sequence-206-L 0.120 0.001 0.000 -

Sequence-207-L 0.095 0.001 0.000 -

Sequence-208-S 0.085 0.001 0.000 -

Sequence-209-V 0.209 0.001 0.000 -

Sequence-210-L 0.104 0.001 0.000 -

Sequence-211-N 0.095 0.001 0.000 -

Sequence-212-Q 0.125 0.001 0.000 -

Sequence-213-M 0.075 0.001 0.000 -

Sequence-214-P 0.082 0.000 0.000 -

Sequence-215-Q 0.125 0.000 0.000 -

Sequence-216-R 0.102 0.000 0.000 -

Sequence-217-H 0.083 0.000 0.000 -

Sequence-218-G 0.072 0.000 0.000 -

Sequence-219-P 0.077 0.000 0.000 -

Sequence-220-D 0.068 0.000 0.000 -

Sequence-221-T 0.069 0.000 0.000 -

Sequence-222-F 0.077 0.000 0.000 -

Sequence-223-F 0.086 0.000 0.000 -

Sequence-224-N 0.073 0.000 0.000 -

Sequence-225-F 0.080 0.000 0.000 -

Sequence-226-P 0.074 0.000 0.000 -

Sequence-227-G 0.072 0.000 0.000 -

Sequence-228-C 0.084 0.000 0.000 -

Sequence-229-S 0.071 0.000 0.000 -

Sequence-230-A 0.081 0.000 0.000 -

Sequence-231-A 0.085 0.000 0.000 -

Sequence-232-A 0.070 0.000 0.000 -

Sequence-233-I 0.088 0.000 0.000 -

Sequence-234-A 0.079 0.000 0.000 -

Sequence-235-L 0.132 0.000 0.000 -

Sequence-236-P 0.070 0.000 0.000 -

Sequence-237-P 0.097 0.000 0.000 -

Sequence-238-I 0.099 0.000 0.000 -

Sequence-239-A 0.068 0.000 0.000 -

Sequence-240-K 0.094 0.000 0.000 -

Sequence-241-W 0.072 0.000 0.000 -

Sequence-242-P 0.076 0.000 0.000 -

Sequence-243-Y 0.080 0.000 0.000 -

Sequence-244-Q 0.074 0.000 0.000 -

Sequence-245-N 0.101 0.000 0.000 -

Sequence-246-G 0.073 0.000 0.000 -

Sequence-247-F 0.070 0.000 0.000 -

Sequence-248-T 0.075 0.000 0.000 -

Sequence-249-L 0.070 0.000 0.000 -

Sequence-250-N 0.074 0.000 0.000 -

Sequence-251-T 0.093 0.000 0.000 -

Sequence-252-W 0.071 0.000 0.000 -

Sequence-253-F 0.082 0.000 0.000 -

Sequence-254-R 0.074 0.000 0.000 -

Sequence-255-M 0.144 0.000 0.000 -

Sequence-256-D 0.083 0.000 0.000 -

Sequence-257-P 0.075 0.000 0.000 -

Sequence-258-L 0.265 0.000 0.000 -

Sequence-259-N 0.088 0.000 0.000 -

Sequence-260-N 0.086 0.000 0.000 -

Sequence-261-I 0.086 0.000 0.000 -

Sequence-262-N 0.074 0.000 0.000 -

Sequence-263-V 0.141 0.000 0.000 -

Sequence-264-D 0.077 0.000 0.000 -

Sequence-265-K 0.076 0.000 0.000 -

Sequence-266-D 0.066 0.000 0.000 -

Sequence-267-K 0.093 0.000 0.000 -

Sequence-268-P 0.073 0.000 0.000 -

Sequence-269-Y 0.086 0.000 0.000 -

Sequence-270-L 0.134 0.000 0.000 -

Sequence-271-Y 0.071 0.000 0.000 -

Sequence-272-C 0.146 0.000 0.000 -

Sequence-273-F 0.066 0.000 0.000 -

Sequence-274-R 0.094 0.000 0.000 -

Sequence-275-T 0.105 0.000 0.000 -

Sequence-276-S 0.093 0.000 0.000 -

Sequence-277-K 0.077 0.000 0.000 -

Sequence-278-G 0.094 0.000 0.000 -

Sequence-279-V 0.080 0.000 0.000 -

Sequence-280-G 0.081 0.000 0.000 -

Sequence-281-Y 0.072 0.000 0.000 -

Sequence-282-S 0.070 0.000 0.000 -

Sequence-283-A 0.080 0.000 0.000 -

Sequence-284-H 0.075 0.000 0.000 -

Sequence-285-F 0.071 0.000 0.000 -

Sequence-286-V 0.086 0.000 0.000 -

Sequence-287-G 0.080 0.000 0.000 -

Sequence-288-N 0.072 0.000 0.000 -

Sequence-289-C 0.071 0.000 0.000 -

Sequence-290-L 0.091 0.000 0.000 -

Sequence-291-I 0.154 0.000 0.000 -

Sequence-292-V 0.092 0.000 0.000 -

Sequence-293-T 0.082 0.000 0.000 -

Sequence-294-S 0.086 0.000 0.000 -

Sequence-295-L 0.142 0.000 0.000 -

Sequence-296-K 0.073 0.000 0.000 -

Sequence-297-S 0.135 0.000 0.000 -

Sequence-298-K 0.078 0.000 0.000 -

Sequence-299-G 0.082 0.000 0.000 -

Sequence-300-K 0.112 0.000 0.000 -

Sequence-301-G 0.073 0.000 0.000 -

Sequence-302-F 0.068 0.000 0.000 -

Sequence-303-Q 0.089 0.000 0.000 -

Sequence-304-H 0.083 0.000 0.000 -

Sequence-305-C 0.096 0.000 0.000 -

Sequence-306-V 0.075 0.000 0.000 -

Sequence-307-K 0.078 0.000 0.000 -

Sequence-308-Y 0.093 0.000 0.000 -

Sequence-309-D 0.070 0.000 0.000 -

Sequence-310-F 0.074 0.000 0.000 -

Sequence-311-Q 0.079 0.000 0.000 -

Sequence-312-P 0.076 0.000 0.000 -

Sequence-313-R 0.077 0.000 0.000 -

Sequence-314-K 0.073 0.000 0.000 -

Sequence-315-W 0.085 0.000 0.000 -

Sequence-316-Y 0.079 0.000 0.000 -

Sequence-317-M 0.112 0.000 0.000 -

Sequence-318-I 0.080 0.000 0.000 -

Sequence-319-S 0.075 0.000 0.000 -

Sequence-320-I 0.086 0.000 0.000 -

Sequence-321-V 0.127 0.000 0.000 -

Sequence-322-H 0.091 0.000 0.000 -

Sequence-323-I 0.122 0.000 0.000 -

Sequence-324-Y 0.070 0.000 0.000 -

Sequence-325-N 0.083 0.000 0.000 -

Sequence-326-R 0.093 0.000 0.000 -

Sequence-327-W 0.072 0.000 0.000 -

Sequence-328-R 0.080 0.000 0.000 -

Sequence-329-N 0.099 0.000 0.000 -

Sequence-330-S 0.070 0.000 0.000 -

Sequence-331-E 0.069 0.000 0.000 -

Sequence-332-I 0.103 0.000 0.000 -

Sequence-333-R 0.070 0.000 0.000 -

Sequence-334-C 0.078 0.000 0.000 -

Sequence-335-Y 0.069 0.000 0.000 -

Sequence-336-V 0.072 0.000 0.000 -

Sequence-337-N 0.072 0.000 0.000 -

Sequence-338-G 0.075 0.000 0.000 -

Sequence-339-Q 0.072 0.000 0.000 -

Sequence-340-L 0.089 0.000 0.000 -

Sequence-341-V 0.084 0.000 0.000 -

Sequence-342-S 0.109 0.000 0.000 -

Sequence-343-Y 0.078 0.000 0.000 -

Sequence-344-G 0.075 0.000 0.000 -

Sequence-345-D 0.067 0.000 0.000 -

Sequence-346-M 0.080 0.000 0.000 -

Sequence-347-A 0.068 0.000 0.000 -

Sequence-348-W 0.143 0.000 0.000 -

Sequence-349-H 0.074 0.000 0.000 -

Sequence-350-V 0.113 0.000 0.000 -

Sequence-351-N 0.075 0.000 0.000 -

Sequence-352-T 0.070 0.000 0.000 -

Sequence-353-N 0.088 0.000 0.000 -

Sequence-354-D 0.069 0.000 0.000 -

Sequence-355-S 0.076 0.000 0.000 -

Sequence-356-Y 0.077 0.000 0.000 -

Sequence-357-D 0.082 0.000 0.000 -

Sequence-358-K 0.072 0.000 0.000 -

Sequence-359-C 0.071 0.000 0.000 -

Sequence-360-F 0.074 0.000 0.000 -

Sequence-361-L 0.148 0.000 0.000 -

Sequence-362-G 0.095 0.000 0.000 -

Sequence-363-S 0.086 0.000 0.000 -

Sequence-364-S 0.080 0.000 0.000 -

Sequence-365-E 0.144 0.000 0.000 -

Sequence-366-T 0.107 0.000 0.000 -

Sequence-367-A 0.078 0.000 0.000 -

Sequence-368-D 0.077 0.000 0.000 -

Sequence-369-A 0.082 0.000 0.000 -

Sequence-370-N 0.074 0.000 0.000 -

Sequence-371-R 0.087 0.000 0.000 -

Sequence-372-V 0.071 0.000 0.000 -

Sequence-373-F 0.094 0.000 0.000 -

Sequence-374-C 0.092 0.000 0.000 -

Sequence-375-G 0.078 0.000 0.000 -

Sequence-376-Q 0.078 0.000 0.000 -

Sequence-377-L 0.174 0.000 0.000 -

Sequence-378-G 0.078 0.000 0.000 -

Sequence-379-A 0.087 0.000 0.000 -

Sequence-380-V 0.080 0.000 0.000 -

Sequence-381-Y 0.066 0.000 0.000 -

Sequence-382-V 0.089 0.000 0.000 -

Sequence-383-F 0.088 0.000 0.000 -

Sequence-384-S 0.079 0.000 0.000 -

Sequence-385-E 0.084 0.000 0.000 -

Sequence-386-A 0.080 0.000 0.000 -

Sequence-387-L 0.112 0.000 0.000 -

Sequence-388-N 0.075 0.000 0.000 -

Sequence-389-P 0.081 0.000 0.000 -

Sequence-390-A 0.068 0.000 0.000 -

Sequence-391-Q 0.079 0.000 0.000 -

Sequence-392-I 0.109 0.000 0.000 -

Sequence-393-F 0.081 0.000 0.000 -

Sequence-394-A 0.082 0.000 0.000 -

Sequence-395-V 0.105 0.000 0.000 -

Sequence-396-H 0.073 0.000 0.000 -

Sequence-397-Q 0.096 0.000 0.000 -

Sequence-398-L 0.215 0.000 0.000 -

Sequence-399-G 0.066 0.000 0.000 -

Sequence-400-P 0.092 0.000 0.000 -

Sequence-401-G 0.067 0.000 0.000 -

Sequence-402-Y 0.074 0.000 0.000 -

Sequence-403-K 0.088 0.000 0.000 -

Sequence-404-S 0.075 0.000 0.000 -

Sequence-405-T 0.072 0.000 0.000 -

Sequence-406-F 0.079 0.003 0.000 -

Sequence-407-K 0.083 0.003 0.000 -

Sequence-408-F 0.080 0.003 0.000 -

Sequence-409-K 0.071 0.003 0.000 -

Sequence-410-S 0.069 0.003 0.000 -

Sequence-411-E 0.082 0.003 0.000 -

Sequence-412-S 0.088 0.003 0.000 -

Sequence-413-D 0.089 0.003 0.000 -

Sequence-414-I 0.257 0.003 0.000 -

Sequence-415-H 0.072 0.002 0.000 -

Sequence-416-L 0.180 0.002 0.000 -

Sequence-417-A 0.092 0.000 0.000 -

Sequence-418-E 0.117 0.000 0.000 -

Sequence-419-H 0.082 0.000 0.000 -

Sequence-420-H 0.102 0.000 0.000 -

Sequence-421-K 0.141 0.000 0.000 -

Sequence-422-Q 0.090 0.000 0.000 -

Sequence-423-V 0.111 0.000 0.000 -

Sequence-424-L 0.111 0.003 0.000 -

Sequence-425-Y 0.112 0.003 0.000 -

Sequence-426-D 0.144 0.003 0.000 -

Sequence-427-G 0.068 0.003 0.000 -

Sequence-428-K 0.071 0.003 0.000 -

Sequence-429-L 0.193 0.003 0.000 -

Sequence-430-A 0.076 0.003 0.000 -

Sequence-431-S 0.170 0.003 0.000 -

Sequence-432-S 0.081 0.003 0.000 -

Sequence-433-I 0.148 0.003 0.000 -

Sequence-434-A 0.103 0.000 0.000 -

Sequence-435-F 0.082 0.000 0.000 -

Sequence-436-S 0.084 0.000 0.000 -

Sequence-437-Y 0.074 0.000 0.000 -

Sequence-438-N 0.097 0.000 0.000 -

Sequence-439-A 0.080 0.000 0.000 -

Sequence-440-K 0.083 0.000 0.000 -

Sequence-441-A 0.066 0.000 0.000 -

Sequence-442-T 0.079 0.000 0.000 -

Sequence-443-D 0.072 0.000 0.000 -

Sequence-444-A 0.074 0.000 0.000 -

Sequence-445-Q 0.071 0.000 0.000 -

Sequence-446-L 0.130 0.000 0.000 -

Sequence-447-C 0.081 0.000 0.000 -

Sequence-448-L 0.123 0.000 0.000 -

Sequence-449-E 0.100 0.000 0.000 -

Sequence-450-S 0.193 0.000 0.000 -

Sequence-451-S 0.152 0.000 0.000 -

Sequence-452-P 0.083 0.000 0.000 -

Sequence-453-K 0.103 0.000 0.000 -

Sequence-454-E 0.071 0.000 0.000 -

Sequence-455-N 0.109 0.000 0.000 -

Sequence-456-A 0.085 0.000 0.000 -

Sequence-457-S 0.076 0.000 0.000 -

Sequence-458-I 0.105 0.000 0.000 -

Sequence-459-F 0.073 0.000 0.000 -

Sequence-460-V 0.098 0.000 0.000 -

Sequence-461-H 0.091 0.000 0.000 -

Sequence-462-S 0.074 0.000 0.000 -

Sequence-463-P 0.076 0.000 0.000 -

Sequence-464-H 0.085 0.000 0.000 -

Sequence-465-A 0.083 0.000 0.000 -

Sequence-466-L 0.100 0.000 0.000 -

Sequence-467-M 0.070 0.000 0.000 -

Sequence-468-L 0.284 0.000 0.000 -

Sequence-469-Q 0.068 0.000 0.000 -

Sequence-470-D 0.085 0.000 0.000 -

Sequence-471-V 0.234 0.000 0.000 -

Sequence-472-K 0.074 0.000 0.000 -

Sequence-473-A 0.100 0.000 0.000 -

Sequence-474-I 0.096 0.000 0.000 -

Sequence-475-V 0.080 0.000 0.000 -

Sequence-476-T 0.102 0.000 0.000 -

Sequence-477-H 0.104 0.000 0.000 -

Sequence-478-S 0.078 0.000 0.000 -

Sequence-479-I 0.123 0.000 0.000 -

Sequence-480-H 0.076 0.000 0.000 -

Sequence-481-S 0.083 0.000 0.000 -

Sequence-482-A 0.079 0.000 0.000 -

Sequence-483-I 0.213 0.000 0.000 -

Sequence-484-H 0.081 0.000 0.000 -

Sequence-485-S 0.069 0.000 0.000 -

Sequence-486-I 0.103 0.000 0.000 -

Sequence-487-G 0.076 0.000 0.000 -

Sequence-488-G 0.098 0.000 0.000 -

Sequence-489-I 0.089 0.000 0.000 -

Sequence-490-Q 0.065 0.000 0.000 -

Sequence-491-V 0.102 0.000 0.000 -

Sequence-492-L 0.136 0.000 0.000 -

Sequence-493-F 0.069 0.000 0.000 -

Sequence-494-P 0.100 0.000 0.000 -

Sequence-495-L 0.082 0.000 0.000 -

Sequence-496-F 0.082 0.000 0.000 -

Sequence-497-A 0.108 0.000 0.000 -

Sequence-498-Q 0.080 0.000 0.000 -

Sequence-499-L 0.130 0.000 0.000 -

Sequence-500-D 0.080 0.000 0.000 -

Sequence-501-N 0.267 0.000 0.000 -

Sequence-502-R 0.089 0.000 0.000 -

Sequence-503-Q 0.086 0.000 0.000 -

Sequence-504-L 0.203 0.000 0.000 -

Sequence-505-N 0.091 0.000 0.000 -

Sequence-506-D 0.089 0.000 0.000 -

Sequence-507-S 0.071 0.000 0.000 -

Sequence-508-Q 0.091 0.000 0.000 -

Sequence-509-V 0.111 0.000 0.000 -

Sequence-510-E 0.076 0.000 0.000 -

Sequence-511-T 0.081 0.000 0.000 -

Sequence-512-T 0.085 0.000 0.000 -

Sequence-513-V 0.083 0.001 0.000 -

Sequence-514-C 0.115 0.001 0.000 -

Sequence-515-A 0.067 0.001 0.000 -

Sequence-516-T 0.074 0.002 0.000 -

Sequence-517-L 0.111 0.006 0.000 -

Sequence-518-L 0.244 0.013 0.000 -

Sequence-519-A 0.109 0.013 0.000 -

Sequence-520-F 0.141 0.013 0.000 -

Sequence-521-L 0.186 0.018 0.000 -

Sequence-522-V 0.186 0.020 0.000 -

Sequence-523-E 0.199 0.020 0.000 -

Sequence-524-L 0.317 0.020 0.000 -

Sequence-525-L 0.507 0.012 0.330 -

Sequence-526-K 0.168 0.009 0.000 -

Sequence-527-S 0.155 0.009 0.000 -

Sequence-528-S 0.076 0.009 0.000 -

Sequence-529-V 0.281 0.009 0.000 -

Sequence-530-A 0.087 0.008 0.000 -

Sequence-531-M 0.124 0.010 0.000 -

Sequence-532-Q 0.077 0.003 0.000 -

Sequence-533-E 0.107 0.003 0.000 -

Sequence-534-Q 0.098 0.003 0.000 -

Sequence-535-M 0.087 0.003 0.000 -

Sequence-536-L 0.135 0.003 0.000 -

Sequence-537-G 0.070 0.003 0.000 -

Sequence-538-G 0.082 0.003 0.000 -

Sequence-539-K 0.068 0.003 0.000 -

Sequence-540-G 0.082 0.003 0.000 -

Sequence-541-F 0.087 0.003 0.000 -

Sequence-542-L 0.142 0.003 0.000 -

Sequence-543-V 0.108 0.003 0.000 -

Sequence-544-I 0.545 0.003 0.220 -

Sequence-545-G 0.084 0.000 0.000 -

Sequence-546-Y 0.105 0.000 0.000 -

Sequence-547-L 0.119 0.000 0.000 -

Sequence-548-L 0.092 0.001 0.000 -

Sequence-549-E 0.124 0.001 0.000 -

Sequence-550-K 0.095 0.001 0.000 -

Sequence-551-S 0.079 0.001 0.000 -

Sequence-552-S 0.088 0.001 0.000 -

Sequence-553-R 0.142 0.001 0.000 -

Sequence-554-V 0.093 0.001 0.000 -

Sequence-555-H 0.083 0.001 0.000 -

Sequence-556-I 0.147 0.001 0.000 -

Sequence-557-T 0.076 0.000 0.000 -

Sequence-558-R 0.083 0.000 0.000 -

Sequence-559-A 0.082 0.000 0.000 -

Sequence-560-V 0.088 0.000 0.000 -

Sequence-561-L 0.104 0.001 0.000 -

Sequence-562-E 0.093 0.001 0.000 -

Sequence-563-Q 0.079 0.001 0.000 -

Sequence-564-F 0.077 0.001 0.000 -

Sequence-565-L 0.301 0.002 0.000 -

Sequence-566-S 0.078 0.002 0.000 -

Sequence-567-F 0.118 0.002 0.000 -

Sequence-568-A 0.082 0.001 0.000 -

Sequence-569-K 0.107 0.001 0.000 -

Sequence-570-Y 0.091 0.001 0.000 -

Sequence-571-L 0.094 0.002 0.000 -

Sequence-572-D 0.084 0.001 0.000 -

Sequence-573-G 0.258 0.001 0.000 -

Sequence-574-L 0.087 0.002 0.000 -

Sequence-575-S 0.085 0.000 0.000 -

Sequence-576-H 0.254 0.000 0.000 -

Sequence-577-G 0.095 0.000 0.000 -

Sequence-578-A 0.078 0.000 0.000 -

Sequence-579-P 0.104 0.000 0.000 -

Sequence-580-L 0.144 0.002 0.000 -

Sequence-581-L 0.112 0.005 0.000 -

Sequence-582-K 0.103 0.005 0.000 -

Sequence-583-Q 0.082 0.005 0.000 -

Sequence-584-L 0.131 0.005 0.000 -

Sequence-585-C 0.090 0.005 0.000 -

Sequence-586-D 0.127 0.005 0.000 -

Sequence-587-H 0.080 0.005 0.000 -

Sequence-588-I 0.198 0.005 0.000 -

Sequence-589-L 0.777 0.005 0.301 -

Sequence-590-F 0.103 0.005 0.000 -

Sequence-591-N 0.163 0.000 0.000 -

Sequence-592-P 0.087 0.000 0.000 -

Sequence-593-A 0.097 0.000 0.000 -

Sequence-594-I 0.170 0.000 0.000 -

Sequence-595-W 0.070 0.000 0.000 -

Sequence-596-I 0.117 0.000 0.000 -

Sequence-597-H 0.079 0.000 0.000 -

Sequence-598-T 0.086 0.000 0.000 -

Sequence-599-P 0.097 0.000 0.000 -

Sequence-600-A 0.066 0.000 0.000 -

Sequence-601-K 0.071 0.000 0.000 -

Sequence-602-V 0.065 0.000 0.000 -

Sequence-603-Q 0.068 0.000 0.000 -

Sequence-604-L 0.155 0.000 0.000 -

Sequence-605-S 0.073 0.000 0.000 -

Sequence-606-L 0.218 0.000 0.000 -

Sequence-607-Y 0.078 0.000 0.000 -

Sequence-608-T 0.106 0.000 0.000 -

Sequence-609-Y 0.113 0.000 0.000 -

Sequence-610-L 0.105 0.000 0.000 -

Sequence-611-S 0.082 0.000 0.000 -

Sequence-612-A 0.121 0.000 0.000 -

Sequence-613-E 0.095 0.000 0.000 -

Sequence-614-F 0.096 0.000 0.000 -

Sequence-615-I 0.360 0.000 0.000 -

Sequence-616-G 0.079 0.000 0.000 -

Sequence-617-T 0.076 0.000 0.000 -

Sequence-618-A 0.075 0.000 0.000 -

Sequence-619-T 0.111 0.000 0.000 -

Sequence-620-I 0.266 0.000 0.000 -

Sequence-621-Y 0.072 0.000 0.000 -

Sequence-622-T 0.072 0.000 0.000 -

Sequence-623-T 0.073 0.000 0.000 -

Sequence-624-I 0.156 0.000 0.000 -

Sequence-625-R 0.073 0.000 0.000 -

Sequence-626-R 0.098 0.000 0.000 -

Sequence-627-V 0.082 0.000 0.000 -

Sequence-628-G 0.072 0.000 0.000 -

Sequence-629-T 0.092 0.000 0.000 -

Sequence-630-V 0.115 0.000 0.000 -

Sequence-631-L 0.090 0.000 0.000 -

Sequence-632-Q 0.087 0.000 0.000 -

Sequence-633-L 0.311 0.000 0.000 -

Sequence-634-M 0.080 0.000 0.000 -

Sequence-635-H 0.133 0.000 0.000 -

Sequence-636-T 0.162 0.000 0.000 -

Sequence-637-L 0.104 0.000 0.000 -

Sequence-638-K 0.075 0.000 0.000 -

Sequence-639-Y 0.154 0.000 0.000 -

Sequence-640-Y 0.072 0.000 0.000 -

Sequence-641-Y 0.209 0.000 0.000 -

Sequence-642-W 0.154 0.000 0.000 -

Sequence-643-V 0.100 0.000 0.000 -

Sequence-644-I 0.093 0.000 0.000 -

Sequence-645-N 0.070 0.000 0.000 -

Sequence-646-P 0.092 0.000 0.000 -

Sequence-647-A 0.072 0.000 0.000 -

Sequence-648-D 0.086 0.000 0.000 -

Sequence-649-S 0.072 0.000 0.000 -

Sequence-650-S 0.072 0.000 0.000 -

Sequence-651-G 0.071 0.000 0.000 -

Sequence-652-I 0.081 0.000 0.000 -

Sequence-653-A 0.072 0.000 0.000 -

Sequence-654-P 0.075 0.000 0.000 -

Sequence-655-K 0.081 0.000 0.000 -

Sequence-656-G 0.069 0.000 0.000 -

Sequence-657-L 0.081 0.000 0.000 -

Sequence-658-D 0.069 0.000 0.000 -

Sequence-659-G 0.102 0.000 0.000 -

Sequence-660-P 0.067 0.000 0.000 -

Sequence-661-R 0.092 0.000 0.000 -

Sequence-662-P 0.080 0.000 0.000 -

Sequence-663-S 0.079 0.000 0.000 -

Sequence-664-Q 0.084 0.000 0.000 -

Sequence-665-K 0.082 0.000 0.000 -

Sequence-666-E 0.068 0.000 0.000 -

Sequence-667-I 0.102 0.001 0.000 -

Sequence-668-I 0.078 0.004 0.000 -

Sequence-669-S 0.071 0.004 0.000 -

Sequence-670-L 0.127 0.007 0.000 -

Sequence-671-R 0.078 0.007 0.000 -

Sequence-672-A 0.136 0.007 0.000 -

Sequence-673-F 0.082 0.007 0.000 -

Sequence-674-M 0.109 0.015 0.000 -

Sequence-675-L 0.203 0.031 0.000 -

Sequence-676-L 0.223 0.055 0.000 -

Sequence-677-F 0.159 0.052 0.000 -

Sequence-678-L 0.708 0.053 0.364 -

Sequence-679-K 0.107 0.051 0.000 -

Sequence-680-Q 0.287 0.051 0.003 -

Sequence-681-L 0.108 0.055 0.000 -

Sequence-682-I 0.240 0.052 0.000 -

Sequence-683-L 0.465 0.054 0.451 -

Sequence-684-K 0.084 0.009 0.000 -

Sequence-685-D 0.356 0.009 0.000 -

Sequence-686-R 0.138 0.009 0.000 -

Sequence-687-G 0.097 0.009 0.000 -

Sequence-688-V 0.163 0.011 0.000 -

Sequence-689-K 0.083 0.011 0.000 -

Sequence-690-E 0.091 0.011 0.000 -

Sequence-691-D 0.104 0.011 0.000 -

Sequence-692-E 0.080 0.011 0.000 -

Sequence-693-L 0.139 0.013 0.000 -

Sequence-694-Q 0.078 0.012 0.000 -

Sequence-695-S 0.075 0.012 0.000 -

Sequence-696-I 0.089 0.012 0.000 -

Sequence-697-L 0.102 0.006 0.000 -

Sequence-698-N 0.090 0.002 0.000 -

Sequence-699-Y 0.181 0.002 0.000 -

Sequence-700-L 0.125 0.003 0.000 -

Sequence-701-L 0.348 0.003 0.000 -

Sequence-702-T 0.357 0.003 0.000 -

Sequence-703-M 0.451 0.003 0.314 -

Sequence-704-H 0.109 0.001 0.000 -

Sequence-705-E 0.258 0.001 0.000 -

Sequence-706-D 0.096 0.001 0.000 -

Sequence-707-E 0.088 0.001 0.000 -

Sequence-708-N 0.085 0.001 0.000 -

Sequence-709-I 0.160 0.002 0.000 -

Sequence-710-H 0.072 0.001 0.000 -

Sequence-711-D 0.093 0.001 0.000 -

Sequence-712-V 0.099 0.002 0.000 -

Sequence-713-L 0.083 0.009 0.000 -

Sequence-714-Q 0.095 0.008 0.000 -

Sequence-715-L 0.245 0.008 0.000 -

Sequence-716-L 0.096 0.008 0.000 -

Sequence-717-V 0.358 0.008 0.000 -

Sequence-718-A 0.256 0.007 0.000 -

Sequence-719-L 0.335 0.007 0.000 -

Sequence-720-M 0.115 0.000 0.000 -

Sequence-721-S 0.194 0.000 0.000 -

Sequence-722-E 0.100 0.000 0.000 -

Sequence-723-H 0.114 0.000 0.000 -

Sequence-724-P 0.129 0.000 0.000 -

Sequence-725-A 0.101 0.000 0.000 -

Sequence-726-S 0.070 0.000 0.000 -

Sequence-727-M 0.067 0.000 0.000 -

Sequence-728-I 0.144 0.000 0.000 -

Sequence-729-P 0.079 0.000 0.000 -

Sequence-730-A 0.075 0.000 0.000 -

Sequence-731-F 0.072 0.000 0.000 -

Sequence-732-D 0.072 0.000 0.000 -

Sequence-733-Q 0.088 0.000 0.000 -

Sequence-734-R 0.072 0.000 0.000 -

Sequence-735-N 0.076 0.000 0.000 -

Sequence-736-G 0.084 0.000 0.000 -

Sequence-737-I 0.088 0.000 0.000 -

Sequence-738-R 0.071 0.000 0.000 -

Sequence-739-V 0.074 0.000 0.000 -

Sequence-740-I 0.159 0.000 0.000 -

Sequence-741-Y 0.076 0.000 0.000 -

Sequence-742-K 0.089 0.000 0.000 -

Sequence-743-L 0.116 0.000 0.000 -

Sequence-744-L 0.133 0.000 0.000 -

Sequence-745-A 0.110 0.000 0.000 -

Sequence-746-S 0.094 0.000 0.000 -

Sequence-747-K 0.074 0.000 0.000 -

Sequence-748-S 0.104 0.000 0.000 -

Sequence-749-E 0.079 0.000 0.000 -

Sequence-750-S 0.097 0.000 0.000 -

Sequence-751-I 0.092 0.000 0.000 -

Sequence-752-W 0.081 0.000 0.000 -

Sequence-753-V 0.107 0.000 0.000 -

Sequence-754-Q 0.070 0.000 0.000 -

Sequence-755-A 0.079 0.000 0.000 -

Sequence-756-L 0.096 0.000 0.000 -

Sequence-757-K 0.067 0.000 0.000 -

Sequence-758-V 0.103 0.000 0.000 -

Sequence-759-L 0.127 0.000 0.000 -

Sequence-760-G 0.114 0.000 0.000 -

Sequence-761-Y 0.124 0.000 0.000 -

Sequence-762-F 0.076 0.000 0.000 -

Sequence-763-L 0.184 0.003 0.000 -

Sequence-764-K 0.121 0.003 0.000 -

Sequence-765-H 0.143 0.003 0.000 -

Sequence-766-L 0.153 0.003 0.000 -

Sequence-767-G 0.076 0.003 0.000 -

Sequence-768-H 0.169 0.003 0.000 -

Sequence-769-K 0.081 0.003 0.000 -

Sequence-770-R 0.071 0.003 0.000 -

Sequence-771-K 0.148 0.003 0.000 -

Sequence-772-V 0.100 0.003 0.000 -

Sequence-773-E 0.090 0.003 0.000 -

Sequence-774-I 0.130 0.003 0.000 -

Sequence-775-M 0.083 0.000 0.000 -

Sequence-776-H 0.091 0.000 0.000 -

Sequence-777-T 0.083 0.000 0.000 -

Sequence-778-H 0.069 0.000 0.000 -

Sequence-779-S 0.093 0.000 0.000 -

Sequence-780-L 0.138 0.002 0.000 -

Sequence-781-F 0.075 0.002 0.000 -

Sequence-782-T 0.090 0.003 0.000 -

Sequence-783-L 0.318 0.003 0.000 -

Sequence-784-L 0.180 0.002 0.000 -

Sequence-785-G 0.164 0.002 0.000 -

Sequence-786-E 0.106 0.002 0.000 -

Sequence-787-R 0.090 0.002 0.000 -

Sequence-788-L 0.528 0.003 0.290 -

Sequence-789-M 0.538 0.000 0.343 -

Sequence-790-L 0.801 0.000 0.483 -

Sequence-791-H 0.075 0.000 0.065 -

Sequence-792-T 0.155 0.000 0.075 -

Sequence-793-N 0.160 0.000 0.089 -

Sequence-794-T 0.077 0.000 0.000 -

Sequence-795-V 0.102 0.000 0.000 -

Sequence-796-T 0.086 0.000 0.000 -

Sequence-797-V 0.101 0.000 0.000 -

Sequence-798-T 0.080 0.000 0.000 -

Sequence-799-T 0.084 0.000 0.000 -

Sequence-800-Y 0.086 0.000 0.000 -

Sequence-801-N 0.066 0.000 0.000 -

Sequence-802-T 0.076 0.000 0.000 -

Sequence-803-L 0.153 0.000 0.000 -

Sequence-804-Y 0.087 0.000 0.000 -

Sequence-805-E 0.077 0.000 0.000 -

Sequence-806-I 0.182 0.000 0.000 -

Sequence-807-L 0.133 0.000 0.000 -

Sequence-808-T 0.087 0.000 0.000 -

Sequence-809-E 0.244 0.000 0.000 -

Sequence-810-Q 0.078 0.000 0.000 -

Sequence-811-V 0.186 0.000 0.000 -

Sequence-812-C 0.150 0.000 0.000 -

Sequence-813-T 0.084 0.000 0.000 -

Sequence-814-Q 0.092 0.000 0.000 -

Sequence-815-V 0.093 0.000 0.000 -

Sequence-816-V 0.135 0.000 0.000 -

Sequence-817-H 0.074 0.000 0.000 -

Sequence-818-K 0.100 0.000 0.000 -

Sequence-819-P 0.066 0.000 0.000 -

Sequence-820-H 0.083 0.000 0.000 -

Sequence-821-P 0.087 0.000 0.000 -

Sequence-822-E 0.073 0.000 0.000 -

Sequence-823-P 0.076 0.000 0.000 -

Sequence-824-D 0.072 0.000 0.000 -

Sequence-825-S 0.073 0.000 0.000 -

Sequence-826-T 0.081 0.000 0.000 -

Sequence-827-V 0.099 0.000 0.000 -

Sequence-828-K 0.081 0.000 0.000 -

Sequence-829-I 0.113 0.000 0.000 -

Sequence-830-Q 0.072 0.000 0.000 -

Sequence-831-N 0.073 0.000 0.000 -

Sequence-832-P 0.079 0.000 0.000 -

Sequence-833-M 0.078 0.000 0.000 -

Sequence-834-I 0.187 0.000 0.000 -

Sequence-835-L 0.129 0.000 0.000 -

Sequence-836-K 0.074 0.000 0.000 -

Sequence-837-V 0.105 0.000 0.000 -

Sequence-838-V 0.094 0.000 0.000 -

Sequence-839-A 0.090 0.000 0.000 -

Sequence-840-T 0.094 0.000 0.000 -

Sequence-841-L 0.113 0.000 0.000 -

Sequence-842-L 0.122 0.000 0.000 -

Sequence-843-K 0.111 0.000 0.000 -

Sequence-844-N 0.143 0.000 0.000 -

Sequence-845-S 0.072 0.000 0.000 -

Sequence-846-T 0.093 0.001 0.000 -

Sequence-847-P 0.112 0.001 0.000 -

Sequence-848-S 0.072 0.001 0.000 -

Sequence-849-A 0.082 0.001 0.000 -

Sequence-850-E 0.089 0.001 0.000 -

Sequence-851-L 0.103 0.005 0.000 -

Sequence-852-M 0.089 0.013 0.000 -

Sequence-853-E 0.087 0.013 0.000 -

Sequence-854-V 0.102 0.016 0.000 -

Sequence-855-R 0.069 0.016 0.000 -

Sequence-856-R 0.109 0.016 0.000 -

Sequence-857-L 0.404 0.033 0.205 -

Sequence-858-F 0.088 0.033 0.000 -

Sequence-859-L 0.566 0.033 0.312 -

Sequence-860-S 0.090 0.025 0.000 -

Sequence-861-D 0.204 0.025 0.000 -

Sequence-862-M 0.114 0.025 0.000 -

Sequence-863-I 0.186 0.020 0.000 -

Sequence-864-K 0.177 0.020 0.000 -

Sequence-865-L 0.108 0.020 0.000 -

Sequence-866-F 0.099 0.000 0.000 -

Sequence-867-S 0.190 0.000 0.000 -

Sequence-868-N 0.084 0.000 0.000 -

Sequence-869-S 0.091 0.000 0.000 -

Sequence-870-R 0.093 0.000 0.000 -

Sequence-871-E 0.090 0.000 0.000 -

Sequence-872-N 0.080 0.000 0.000 -

Sequence-873-R 0.085 0.000 0.000 -

Sequence-874-R 0.085 0.000 0.000 -

Sequence-875-C 0.093 0.000 0.000 -

Sequence-876-L 0.091 0.000 0.000 -

Sequence-877-L 0.098 0.000 0.000 -

Sequence-878-Q 0.141 0.000 0.000 -

Sequence-879-C 0.102 0.000 0.000 -

Sequence-880-S 0.069 0.000 0.000 -

Sequence-881-V 0.154 0.000 0.000 -

Sequence-882-W 0.132 0.000 0.000 -

Sequence-883-Q 0.090 0.000 0.000 -

Sequence-884-D 0.076 0.000 0.000 -

Sequence-885-W 0.077 0.000 0.000 -

Sequence-886-M 0.079 0.000 0.000 -

Sequence-887-F 0.083 0.000 0.000 -

Sequence-888-S 0.075 0.000 0.000 -

Sequence-889-L 0.133 0.000 0.000 -

Sequence-890-G 0.071 0.000 0.000 -

Sequence-891-Y 0.082 0.000 0.000 -

Sequence-892-I 0.214 0.000 0.000 -

Sequence-893-N 0.073 0.000 0.000 -

Sequence-894-P 0.137 0.000 0.000 -

Sequence-895-K 0.077 0.000 0.000 -

Sequence-896-S 0.080 0.000 0.000 -

Sequence-897-S 0.070 0.000 0.000 -

Sequence-898-E 0.076 0.000 0.000 -

Sequence-899-E 0.078 0.000 0.000 -

Sequence-900-Q 0.068 0.000 0.000 -

Sequence-901-K 0.092 0.000 0.000 -

Sequence-902-I 0.076 0.000 0.000 -

Sequence-903-T 0.074 0.000 0.000 -

Sequence-904-E 0.076 0.000 0.000 -

Sequence-905-M 0.089 0.000 0.000 -

Sequence-906-V 0.082 0.000 0.000 -

Sequence-907-Y 0.085 0.000 0.000 -

Sequence-908-N 0.081 0.000 0.000 -

Sequence-909-I 0.093 0.000 0.000 -

Sequence-910-F 0.091 0.000 0.000 -

Sequence-911-R 0.125 0.000 0.000 -

Sequence-912-I 0.114 0.000 0.000 -

Sequence-913-L 0.110 0.000 0.000 -

Sequence-914-L 0.182 0.000 0.000 -

Sequence-915-Y 0.255 0.000 0.000 -

Sequence-916-H 0.100 0.000 0.000 -

Sequence-917-A 0.095 0.000 0.000 -

Sequence-918-I 0.456 0.000 0.140 -

Sequence-919-K 0.092 0.000 0.000 -

Sequence-920-Y 0.082 0.000 0.000 -

Sequence-921-E 0.091 0.000 0.000 -

Sequence-922-W 0.101 0.000 0.000 -

Sequence-923-G 0.087 0.000 0.000 -

Sequence-924-G 0.069 0.000 0.000 -

Sequence-925-W 0.069 0.000 0.000 -

Sequence-926-R 0.065 0.000 0.000 -

Sequence-927-V 0.086 0.000 0.000 -

Sequence-928-W 0.081 0.000 0.000 -

Sequence-929-V 0.134 0.000 0.000 -

Sequence-930-D 0.072 0.000 0.000 -

Sequence-931-T 0.073 0.000 0.000 -

Sequence-932-L 0.103 0.000 0.000 -

Sequence-933-S 0.066 0.000 0.000 -

Sequence-934-I 0.144 0.000 0.000 -

Sequence-935-A 0.073 0.000 0.000 -

Sequence-936-H 0.099 0.000 0.000 -

Sequence-937-S 0.078 0.000 0.000 -

Sequence-938-K 0.081 0.000 0.000 -

Sequence-939-V 0.104 0.000 0.000 -

Sequence-940-T 0.071 0.000 0.000 -

Sequence-941-Y 0.148 0.000 0.000 -

Sequence-942-E 0.080 0.000 0.000 -

Sequence-943-A 0.074 0.000 0.000 -

Sequence-944-H 0.099 0.000 0.000 -

Sequence-945-K 0.070 0.000 0.000 -

Sequence-946-E 0.072 0.000 0.000 -

Sequence-947-Y 0.086 0.000 0.000 -

Sequence-948-L 0.146 0.000 0.000 -

Sequence-949-A 0.075 0.000 0.000 -

Sequence-950-K 0.144 0.000 0.000 -

Sequence-951-M 0.072 0.000 0.000 -

Sequence-952-Y 0.084 0.000 0.000 -

Sequence-953-E 0.192 0.000 0.000 -

Sequence-954-E 0.074 0.000 0.000 -

Sequence-955-Y 0.103 0.000 0.000 -

Sequence-956-Q 0.094 0.000 0.000 -

Sequence-957-R 0.170 0.000 0.000 -

Sequence-958-Q 0.086 0.000 0.000 -

Sequence-959-E 0.091 0.000 0.000 -

Sequence-960-E 0.098 0.000 0.000 -

Sequence-961-E 0.080 0.000 0.000 -

Sequence-962-N 0.074 0.000 0.000 -

Sequence-963-I 0.106 0.000 0.000 -

Sequence-964-K 0.075 0.000 0.000 -

Sequence-965-K 0.104 0.000 0.000 -

Sequence-966-G 0.077 0.000 0.000 -

Sequence-967-K 0.089 0.000 0.000 -

Sequence-968-K 0.101 0.000 0.000 -

Sequence-969-G 0.069 0.000 0.000 -

Sequence-970-N 0.091 0.000 0.000 -

Sequence-971-V 0.118 0.000 0.000 -

Sequence-972-S 0.073 0.000 0.000 -

Sequence-973-T 0.075 0.000 0.000 -

Sequence-974-I 0.096 0.000 0.000 -

Sequence-975-S 0.070 0.000 0.000 -

Sequence-976-G 0.077 0.000 0.000 -

Sequence-977-L 0.081 0.000 0.000 -

Sequence-978-S 0.076 0.000 0.000 -

Sequence-979-S 0.109 0.000 0.000 -

Sequence-980-Q 0.068 0.000 0.000 -

Sequence-981-T 0.088 0.000 0.000 -

Sequence-982-A 0.087 0.000 0.000 -

Sequence-983-G 0.073 0.000 0.000 -

Sequence-984-A 0.077 0.000 0.000 -

Sequence-985-K 0.086 0.000 0.000 -

Sequence-986-G 0.077 0.000 0.000 -

Sequence-987-G 0.072 0.000 0.000 -

Sequence-988-M 0.079 0.009 0.000 -

Sequence-989-E 0.082 0.009 0.000 -

Sequence-990-I 0.158 0.009 0.000 -

Sequence-991-R 0.071 0.008 0.000 -

Sequence-992-E 0.098 0.008 0.000 -

Sequence-993-I 0.141 0.008 0.000 -

Sequence-994-E 0.069 0.008 0.000 -

Sequence-995-D 0.090 0.008 0.000 -

Sequence-996-L 0.273 0.008 0.000 -

Sequence-997-S 0.076 0.000 0.000 -

Sequence-998-Q 0.131 0.000 0.000 -

Sequence-999-S 0.081 0.000 0.000 -

Sequence-1000-Q 0.100 0.000 0.000 -

Sequence-1001-S 0.104 0.000 0.000 -

Sequence-1002-P 0.086 0.000 0.000 -

Sequence-1003-E 0.077 0.000 0.000 -

Sequence-1004-S 0.074 0.000 0.000 -

Sequence-1005-E 0.076 0.000 0.000 -

Sequence-1006-T 0.085 0.000 0.000 -

Sequence-1007-D 0.074 0.000 0.000 -

Sequence-1008-Y 0.075 0.000 0.000 -

Sequence-1009-P 0.074 0.000 0.000 -

Sequence-1010-V 0.091 0.000 0.000 -

Sequence-1011-S 0.083 0.000 0.000 -

Sequence-1012-T 0.076 0.000 0.000 -

Sequence-1013-D 0.096 0.000 0.000 -

Sequence-1014-T 0.082 0.000 0.000 -

Sequence-1015-R 0.095 0.000 0.000 -

Sequence-1016-D 0.090 0.000 0.000 -

Sequence-1017-L 0.148 0.000 0.000 -

Sequence-1018-L 0.155 0.000 0.000 -

Sequence-1019-M 0.412 0.000 0.197 -

Sequence-1020-S 0.092 0.000 0.000 -

Sequence-1021-T 0.291 0.000 0.000 -

Sequence-1022-K 0.173 0.000 0.000 -

Sequence-1023-V 0.112 0.000 0.000 -

Sequence-1024-S 0.106 0.000 0.000 -

Sequence-1025-D 0.080 0.000 0.000 -

Sequence-1026-D 0.093 0.000 0.000 -

Sequence-1027-I 0.168 0.000 0.000 -

Sequence-1028-L 0.207 0.000 0.000 -

Sequence-1029-G 0.081 0.000 0.000 -

Sequence-1030-S 0.084 0.000 0.000 -

Sequence-1031-S 0.076 0.000 0.000 -

Sequence-1032-D 0.082 0.000 0.000 -

Sequence-1033-R 0.213 0.000 0.000 -

Sequence-1034-P 0.109 0.000 0.000 -

Sequence-1035-G 0.082 0.000 0.000 -

Sequence-1036-S 0.070 0.000 0.000 -

Sequence-1037-G 0.080 0.000 0.000 -

Sequence-1038-V 0.097 0.000 0.000 -

Sequence-1039-H 0.070 0.000 0.000 -

Sequence-1040-V 0.089 0.000 0.000 -

Sequence-1041-E 0.067 0.000 0.000 -

Sequence-1042-V 0.081 0.001 0.000 -

Sequence-1043-H 0.079 0.001 0.000 -

Sequence-1044-D 0.074 0.001 0.000 -

Sequence-1045-L 0.147 0.011 0.000 -

Sequence-1046-L 0.081 0.027 0.000 -

Sequence-1047-V 0.116 0.033 0.000 -

Sequence-1048-D 0.083 0.032 0.000 -

Sequence-1049-I 0.239 0.058 0.000 -

Sequence-1050-K 0.105 0.059 0.000 -

Sequence-1051-A 0.181 0.059 0.000 -

Sequence-1052-E 0.103 0.059 0.000 -

Sequence-1053-K 0.098 0.061 0.000 -

Sequence-1054-V 0.175 0.083 0.000 -

Sequence-1055-E 0.091 0.083 0.000 -

Sequence-1056-A 0.112 0.083 0.000 -

Sequence-1057-T 0.068 0.091 0.000 -

Sequence-1058-E 0.076 0.091 0.000 -

Sequence-1059-V 0.101 0.208 0.000 -

Sequence-1060-K 0.076 0.208 0.000 -

Sequence-1061-L 0.106 0.211 0.000 -

Sequence-1062-D 0.090 0.210 0.000 -

Sequence-1063-D 0.084 0.210 0.000 -

Sequence-1064-M 0.109 0.215 0.000 -

Sequence-1065-D 0.103 0.213 0.000 -

Sequence-1066-L 0.664 0.213 0.457 -

Sequence-1067-S 0.077 0.008 0.000 -

Sequence-1068-P 0.319 0.008 0.000 -

Sequence-1069-E 0.081 0.008 0.000 -

Sequence-1070-T 0.193 0.008 0.000 -

Sequence-1071-L 0.554 0.008 0.410 -

Sequence-1072-V 0.079 0.000 0.000 -

Sequence-1073-G 0.134 0.000 0.000 -

Sequence-1074-G 0.073 0.000 0.000 -

Sequence-1075-E 0.081 0.000 0.000 -

Sequence-1076-N 0.130 0.000 0.000 -

Sequence-1077-G 0.105 0.000 0.000 -

Sequence-1078-A 0.085 0.000 0.000 -

Sequence-1079-L 0.144 0.000 0.000 -

Sequence-1080-V 0.073 0.001 0.000 -

Sequence-1081-E 0.087 0.001 0.000 -

Sequence-1082-V 0.090 0.001 0.000 -

Sequence-1083-E 0.072 0.001 0.000 -

Sequence-1084-S 0.088 0.001 0.000 -

Sequence-1085-L 0.111 0.001 0.000 -

Sequence-1086-L 0.124 0.001 0.000 -

Sequence-1087-D 0.162 0.000 0.000 -

Sequence-1088-N 0.125 0.000 0.000 -

Sequence-1089-V 0.082 0.000 0.000 -

Sequence-1090-Y 0.104 0.000 0.000 -

Sequence-1091-S 0.109 0.000 0.000 -

Sequence-1092-A 0.077 0.000 0.000 -

Sequence-1093-A 0.098 0.000 0.000 -

Sequence-1094-V 0.117 0.000 0.000 -

Sequence-1095-E 0.095 0.000 0.000 -

Sequence-1096-K 0.080 0.000 0.000 -

Sequence-1097-L 0.089 0.000 0.000 -

Sequence-1098-Q 0.068 0.000 0.000 -

Sequence-1099-N 0.106 0.000 0.000 -

Sequence-1100-N 0.072 0.000 0.000 -

Sequence-1101-V 0.077 0.000 0.000 -

Sequence-1102-H 0.080 0.000 0.000 -

Sequence-1103-G 0.073 0.000 0.000 -

Sequence-1104-S 0.079 0.000 0.000 -

Sequence-1105-V 0.092 0.000 0.000 -

Sequence-1106-G 0.090 0.000 0.000 -

Sequence-1107-I 0.114 0.000 0.000 -

Sequence-1108-I 0.100 0.000 0.000 -

Sequence-1109-K 0.073 0.000 0.000 -

Sequence-1110-K 0.080 0.000 0.000 -

Sequence-1111-N 0.074 0.000 0.000 -

Sequence-1112-E 0.082 0.000 0.000 -

Sequence-1113-E 0.075 0.000 0.000 -

Sequence-1114-K 0.070 0.000 0.000 -

Sequence-1115-D 0.072 0.000 0.000 -

Sequence-1116-N 0.082 0.000 0.000 -

Sequence-1117-G 0.080 0.000 0.000 -

Sequence-1118-P 0.084 0.000 0.000 -

Sequence-1119-L 0.099 0.021 0.000 -

Sequence-1120-I 0.102 0.038 0.000 -

Sequence-1121-T 0.119 0.038 0.000 -

Sequence-1122-L 0.146 0.038 0.000 -

Sequence-1123-A 0.092 0.038 0.000 -

Sequence-1124-D 0.189 0.038 0.000 -

Sequence-1125-E 0.085 0.038 0.000 -

Sequence-1126-K 0.097 0.038 0.000 -

Sequence-1127-E 0.083 0.038 0.000 -

Sequence-1128-E 0.101 0.038 0.000 -

Sequence-1129-L 0.723 0.038 0.308 -

Sequence-1130-P 0.078 0.000 0.000 -

Sequence-1131-N 0.111 0.000 0.000 -

Sequence-1132-S 0.084 0.000 0.000 -

Sequence-1133-S 0.072 0.000 0.000 -

Sequence-1134-T 0.081 0.000 0.000 -

Sequence-1135-P 0.074 0.000 0.000 -

Sequence-1136-F 0.090 0.000 0.000 -

Sequence-1137-L 0.150 0.000 0.000 -

Sequence-1138-F 0.092 0.000 0.000 -

Sequence-1139-D 0.151 0.000 0.000 -

Sequence-1140-K 0.072 0.000 0.000 -

Sequence-1141-I 0.154 0.000 0.000 -

Sequence-1142-P 0.080 0.000 0.000 -

Sequence-1143-R 0.073 0.000 0.000 -

Sequence-1144-Q 0.092 0.000 0.000 -

Sequence-1145-E 0.080 0.000 0.000 -

Sequence-1146-E 0.099 0.000 0.000 -

Sequence-1147-K 0.108 0.000 0.000 -

Sequence-1148-L 0.097 0.000 0.000 -

Sequence-1149-L 0.109 0.000 0.000 -

Sequence-1150-P 0.143 0.000 0.000 -

Sequence-1151-E 0.096 0.000 0.000 -

Sequence-1152-L 0.170 0.000 0.000 -

Sequence-1153-S 0.087 0.000 0.000 -

Sequence-1154-S 0.351 0.000 0.000 -

Sequence-1155-N 0.078 0.000 0.000 -

Sequence-1156-H 0.127 0.000 0.000 -

Sequence-1157-I 0.297 0.000 0.000 -

Sequence-1158-I 0.252 0.000 0.000 -

Sequence-1159-P 0.078 0.000 0.000 -

Sequence-1160-N 0.073 0.000 0.000 -

Sequence-1161-I 0.111 0.001 0.000 -

Sequence-1162-Q 0.078 0.001 0.000 -

Sequence-1163-D 0.074 0.001 0.000 -

Sequence-1164-T 0.072 0.001 0.000 -

Sequence-1165-Q 0.070 0.001 0.000 -

Sequence-1166-V 0.084 0.002 0.000 -

Sequence-1167-H 0.070 0.002 0.000 -

Sequence-1168-L 0.141 0.004 0.000 -

Sequence-1169-G 0.080 0.004 0.000 -

Sequence-1170-V 0.087 0.004 0.000 -

Sequence-1171-S 0.072 0.004 0.000 -

Sequence-1172-D 0.070 0.004 0.000 -

Sequence-1173-D 0.155 0.004 0.000 -

Sequence-1174-L 0.106 0.005 0.000 -

Sequence-1175-G 0.092 0.004 0.000 -

Sequence-1176-L 0.377 0.005 0.000 -

Sequence-1177-L 0.201 0.002 0.000 -

Sequence-1178-A 0.120 0.002 0.000 -

Sequence-1179-H 0.221 0.002 0.000 -

Sequence-1180-M 0.084 0.002 0.000 -

Sequence-1181-T 0.117 0.002 0.000 -

Sequence-1182-A 0.118 0.002 0.000 -

Sequence-1183-S 0.095 0.002 0.000 -

Sequence-1184-V 0.109 0.002 0.000 -

Sequence-1185-E 0.079 0.002 0.000 -

Sequence-1186-L 0.327 0.003 0.000 -

Sequence-1187-T 0.071 0.001 0.000 -

Sequence-1188-C 0.090 0.001 0.000 -

Sequence-1189-T 0.086 0.001 0.000 -

Sequence-1190-S 0.079 0.001 0.000 -

Sequence-1191-S 0.091 0.001 0.000 -

Sequence-1192-I 0.334 0.001 0.000 -

Sequence-1193-M 0.089 0.002 0.000 -

Sequence-1194-E 0.086 0.002 0.000 -

Sequence-1195-E 0.115 0.002 0.000 -

Sequence-1196-K 0.091 0.002 0.000 -

Sequence-1197-D 0.083 0.002 0.000 -

Sequence-1198-F 0.084 0.002 0.000 -

Sequence-1199-R 0.079 0.002 0.000 -

Sequence-1200-I 0.139 0.002 0.000 -

Sequence-1201-H 0.094 0.000 0.000 -

Sequence-1202-T 0.137 0.000 0.000 -

Sequence-1203-T 0.111 0.000 0.000 -

Sequence-1204-S 0.085 0.000 0.000 -

Sequence-1205-D 0.094 0.000 0.000 -

Sequence-1206-G 0.071 0.000 0.000 -

Sequence-1207-V 0.085 0.000 0.000 -

Sequence-1208-S 0.072 0.000 0.000 -

Sequence-1209-S 0.075 0.000 0.000 -

Sequence-1210-V 0.113 0.000 0.000 -

Sequence-1211-S 0.077 0.000 0.000 -

Sequence-1212-E 0.084 0.000 0.000 -

Sequence-1213-R 0.089 0.000 0.000 -

Sequence-1214-E 0.069 0.000 0.000 -

Sequence-1215-L 0.152 0.000 0.000 -

Sequence-1216-A 0.075 0.000 0.000 -

Sequence-1217-S 0.088 0.000 0.000 -

Sequence-1218-S 0.081 0.000 0.000 -

Sequence-1219-T 0.145 0.000 0.000 -

Sequence-1220-K 0.103 0.000 0.000 -

Sequence-1221-G 0.079 0.000 0.000 -

Sequence-1222-L 0.157 0.000 0.000 -

Sequence-1223-D 0.070 0.000 0.000 -

Sequence-1224-Y 0.165 0.000 0.000 -

Sequence-1225-A 0.076 0.000 0.000 -

Sequence-1226-E 0.097 0.000 0.000 -

Sequence-1227-M 0.132 0.000 0.000 -

Sequence-1228-T 0.080 0.000 0.000 -

Sequence-1229-A 0.103 0.000 0.000 -

Sequence-1230-T 0.087 0.000 0.000 -

Sequence-1231-T 0.118 0.000 0.000 -

Sequence-1232-L 0.220 0.000 0.000 -

Sequence-1233-E 0.077 0.000 0.000 -

Sequence-1234-T 0.088 0.000 0.000 -

Sequence-1235-E 0.095 0.000 0.000 -

Sequence-1236-S 0.083 0.000 0.000 -

Sequence-1237-S 0.117 0.000 0.000 -

Sequence-1238-N 0.089 0.000 0.000 -

Sequence-1239-S 0.083 0.000 0.000 -

Sequence-1240-K 0.079 0.000 0.000 -

Sequence-1241-A 0.096 0.000 0.000 -

Sequence-1242-V 0.102 0.000 0.000 -

Sequence-1243-P 0.077 0.000 0.000 -

Sequence-1244-N 0.076 0.000 0.000 -

Sequence-1245-V 0.073 0.000 0.000 -

Sequence-1246-D 0.070 0.000 0.000 -

Sequence-1247-A 0.112 0.000 0.000 -

Sequence-1248-G 0.076 0.000 0.000 -

Sequence-1249-S 0.071 0.000 0.000 -

Sequence-1250-I 0.094 0.000 0.000 -

Sequence-1251-I 0.104 0.000 0.000 -

Sequence-1252-S 0.077 0.000 0.000 -

Sequence-1253-D 0.079 0.000 0.000 -

Sequence-1254-T 0.074 0.000 0.000 -

Sequence-1255-E 0.080 0.000 0.000 -

Sequence-1256-R 0.084 0.000 0.000 -

Sequence-1257-S 0.071 0.000 0.000 -

Sequence-1258-D 0.082 0.000 0.000 -

Sequence-1259-D 0.127 0.000 0.000 -

Sequence-1260-G 0.076 0.000 0.000 -

Sequence-1261-K 0.105 0.000 0.000 -

Sequence-1262-E 0.086 0.000 0.000 -

Sequence-1263-S 0.078 0.000 0.000 -

Sequence-1264-G 0.092 0.000 0.000 -

Sequence-1265-K 0.076 0.000 0.000 -

Sequence-1266-E 0.092 0.000 0.000 -

Sequence-1267-I 0.111 0.000 0.000 -

Sequence-1268-R 0.082 0.000 0.000 -

Sequence-1269-K 0.089 0.000 0.000 -

Sequence-1270-I 0.112 0.000 0.000 -

Sequence-1271-Q 0.077 0.000 0.000 -

Sequence-1272-T 0.144 0.000 0.000 -

Sequence-1273-T 0.096 0.000 0.000 -

Sequence-1274-A 0.077 0.000 0.000 -

Sequence-1275-T 0.082 0.000 0.000 -

Sequence-1276-T 0.079 0.000 0.000 -

Sequence-1277-Q 0.085 0.000 0.000 -

Sequence-1278-A 0.075 0.000 0.000 -

Sequence-1279-V 0.088 0.000 0.000 -

Sequence-1280-Q 0.072 0.000 0.000 -

Sequence-1281-G 0.073 0.000 0.000 -

Sequence-1282-R 0.071 0.000 0.000 -

Sequence-1283-S 0.079 0.000 0.000 -

Sequence-1284-S 0.086 0.000 0.000 -

Sequence-1285-T 0.080 0.005 0.000 -

Sequence-1286-Q 0.076 0.005 0.000 -

Sequence-1287-Q 0.081 0.005 0.000 -

Sequence-1288-D 0.090 0.005 0.000 -

Sequence-1289-R 0.065 0.005 0.000 -

Sequence-1290-D 0.077 0.005 0.000 -

Sequence-1291-L 0.198 0.006 0.000 -

Sequence-1292-R 0.074 0.006 0.000 -

Sequence-1293-V 0.205 0.006 0.000 -

Sequence-1294-D 0.074 0.006 0.000 -

Sequence-1295-L 0.394 0.006 0.000 -

Sequence-1296-G 0.082 0.001 0.000 -

Sequence-1297-F 0.231 0.001 0.000 -

Sequence-1298-R 0.179 0.000 0.000 -

Sequence-1299-G 0.078 0.000 0.000 -

Sequence-1300-M 0.240 0.000 0.000 -

Sequence-1301-P 0.091 0.000 0.000 -

Sequence-1302-M 0.166 0.000 0.000 -

Sequence-1303-T 0.070 0.000 0.000 -

Sequence-1304-E 0.095 0.000 0.000 -

Sequence-1305-E 0.075 0.000 0.000 -

Sequence-1306-Q 0.066 0.000 0.000 -

Sequence-1307-R 0.071 0.000 0.000 -

Sequence-1308-R 0.078 0.000 0.000 -

Sequence-1309-Q 0.075 0.000 0.000 -

Sequence-1310-F 0.108 0.000 0.000 -

Sequence-1311-S 0.088 0.000 0.000 -

Sequence-1312-P 0.069 0.000 0.000 -

Sequence-1313-G 0.072 0.000 0.000 -

Sequence-1314-P 0.067 0.000 0.000 -

Sequence-1315-R 0.090 0.000 0.000 -

Sequence-1316-T 0.082 0.000 0.000 -

Sequence-1317-T 0.075 0.000 0.000 -

Sequence-1318-M 0.084 0.000 0.000 -

Sequence-1319-F 0.081 0.000 0.000 -

Sequence-1320-R 0.076 0.000 0.000 -

Sequence-1321-I 0.103 0.000 0.000 -

Sequence-1322-P 0.084 0.000 0.000 -

Sequence-1323-E 0.073 0.000 0.000 -

Sequence-1324-F 0.105 0.000 0.000 -

Sequence-1325-K 0.088 0.000 0.000 -

Sequence-1326-W 0.095 0.000 0.000 -

Sequence-1327-S 0.073 0.000 0.000 -

Sequence-1328-P 0.071 0.000 0.000 -

Sequence-1329-M 0.097 0.000 0.000 -

Sequence-1330-H 0.084 0.000 0.000 -

Sequence-1331-Q 0.084 0.000 0.000 -

Sequence-1332-R 0.068 0.000 0.000 -

Sequence-1333-L 0.121 0.002 0.000 -

Sequence-1334-L 0.105 0.007 0.000 -

Sequence-1335-T 0.087 0.007 0.000 -

Sequence-1336-D 0.127 0.007 0.000 -

Sequence-1337-L 0.195 0.007 0.000 -

Sequence-1338-L 0.397 0.008 0.000 -

Sequence-1339-F 0.145 0.008 0.000 -

Sequence-1340-A 0.167 0.008 0.000 -

Sequence-1341-L 0.495 0.008 0.313 -

Sequence-1342-E 0.178 0.003 0.000 -

Sequence-1343-T 0.642 0.003 0.420 -

Sequence-1344-D 0.104 0.003 0.000 -

Sequence-1345-V 0.141 0.003 0.000 -

Sequence-1346-H 0.111 0.002 0.000 -

Sequence-1347-V 0.154 0.002 0.000 -

Sequence-1348-W 0.109 0.000 0.000 -

Sequence-1349-R 0.073 0.000 0.000 -

Sequence-1350-S 0.087 0.000 0.000 -

Sequence-1351-H 0.100 0.000 0.000 -

Sequence-1352-S 0.072 0.000 0.000 -

Sequence-1353-T 0.077 0.000 0.000 -

Sequence-1354-K 0.082 0.000 0.000 -

Sequence-1355-S 0.067 0.000 0.000 -

Sequence-1356-V 0.078 0.000 0.000 -

Sequence-1357-M 0.076 0.000 0.000 -

Sequence-1358-D 0.086 0.000 0.000 -

Sequence-1359-F 0.088 0.000 0.000 -

Sequence-1360-V 0.069 0.000 0.000 -

Sequence-1361-N 0.076 0.000 0.000 -

Sequence-1362-S 0.079 0.000 0.000 -

Sequence-1363-N 0.072 0.000 0.000 -

Sequence-1364-E 0.094 0.000 0.000 -

Sequence-1365-N 0.079 0.000 0.000 -

Sequence-1366-I 0.145 0.000 0.000 -

Sequence-1367-I 0.071 0.000 0.000 -

Sequence-1368-F 0.087 0.000 0.000 -

Sequence-1369-V 0.081 0.000 0.000 -

Sequence-1370-H 0.079 0.000 0.000 -

Sequence-1371-N 0.091 0.000 0.000 -

Sequence-1372-T 0.072 0.000 0.000 -

Sequence-1373-I 0.091 0.000 0.000 -

Sequence-1374-H 0.074 0.000 0.000 -

Sequence-1375-L 0.126 0.000 0.000 -

Sequence-1376-I 0.139 0.000 0.000 -

Sequence-1377-S 0.078 0.000 0.000 -

Sequence-1378-Q 0.070 0.000 0.000 -

Sequence-1379-M 0.090 0.000 0.000 -

Sequence-1380-V 0.110 0.000 0.000 -

Sequence-1381-D 0.083 0.000 0.000 -

Sequence-1382-N 0.110 0.000 0.000 -

Sequence-1383-I 0.077 0.000 0.000 -

Sequence-1384-I 0.126 0.000 0.000 -

Sequence-1385-I 0.147 0.000 0.000 -

Sequence-1386-A 0.076 0.000 0.000 -

Sequence-1387-C 0.099 0.000 0.000 -

Sequence-1388-G 0.076 0.000 0.000 -

Sequence-1389-G 0.073 0.000 0.000 -

Sequence-1390-I 0.087 0.000 0.000 -

Sequence-1391-L 0.113 0.000 0.000 -

Sequence-1392-P 0.106 0.000 0.000 -

Sequence-1393-L 0.440 0.000 0.179 -

Sequence-1394-L 0.086 0.000 0.000 -

Sequence-1395-S 0.112 0.000 0.000 -

Sequence-1396-A 0.086 0.000 0.000 -

Sequence-1397-A 0.071 0.000 0.000 -

Sequence-1398-T 0.073 0.000 0.000 -

Sequence-1399-S 0.096 0.000 0.000 -

Sequence-1400-P 0.083 0.000 0.000 -

Sequence-1401-T 0.110 0.000 0.000 -

Sequence-1402-G 0.077 0.000 0.000 -

Sequence-1403-S 0.082 0.000 0.000 -

Sequence-1404-K 0.072 0.000 0.000 -

Sequence-1405-T 0.066 0.000 0.000 -

Sequence-1406-E 0.088 0.000 0.000 -

Sequence-1407-L 0.092 0.000 0.000 -

Sequence-1408-E 0.069 0.000 0.000 -

Sequence-1409-N 0.104 0.000 0.000 -

Sequence-1410-I 0.117 0.000 0.000 -

Sequence-1411-E 0.072 0.000 0.000 -

Sequence-1412-V 0.277 0.000 0.000 -

Sequence-1413-T 0.093 0.000 0.000 -

Sequence-1414-Q 0.109 0.000 0.000 -

Sequence-1415-G 0.096 0.000 0.000 -

Sequence-1416-M 0.087 0.000 0.000 -

Sequence-1417-S 0.101 0.000 0.000 -

Sequence-1418-A 0.096 0.000 0.000 -

Sequence-1419-E 0.077 0.000 0.000 -

Sequence-1420-T 0.071 0.000 0.000 -

Sequence-1421-A 0.091 0.000 0.000 -

Sequence-1422-V 0.073 0.000 0.000 -

Sequence-1423-T 0.070 0.000 0.000 -

Sequence-1424-F 0.111 0.000 0.000 -

Sequence-1425-L 0.146 0.002 0.000 -

Sequence-1426-S 0.070 0.002 0.000 -

Sequence-1427-R 0.129 0.002 0.000 -

Sequence-1428-L 0.146 0.002 0.000 -

Sequence-1429-M 0.115 0.002 0.000 -

Sequence-1430-A 0.388 0.002 0.000 -

Sequence-1431-M 0.104 0.002 0.000 -

Sequence-1432-V 0.081 0.002 0.000 -

Sequence-1433-D 0.156 0.002 0.000 -

Sequence-1434-V 0.122 0.002 0.000 -

Sequence-1435-L 0.134 0.001 0.000 -

Sequence-1436-V 0.113 0.001 0.000 -

Sequence-1437-F 0.104 0.001 0.000 -

Sequence-1438-A 0.091 0.001 0.000 -

Sequence-1439-S 0.075 0.001 0.000 -

Sequence-1440-S 0.086 0.001 0.000 -

Sequence-1441-L 0.104 0.001 0.000 -

Sequence-1442-N 0.074 0.000 0.000 -

Sequence-1443-F 0.180 0.000 0.000 -

Sequence-1444-S 0.071 0.000 0.000 -

Sequence-1445-E 0.081 0.000 0.000 -

Sequence-1446-I 0.154 0.000 0.000 -

Sequence-1447-E 0.075 0.000 0.000 -

Sequence-1448-A 0.102 0.000 0.000 -

Sequence-1449-E 0.090 0.000 0.000 -

Sequence-1450-K 0.081 0.000 0.000 -

Sequence-1451-N 0.081 0.000 0.000 -

Sequence-1452-M 0.080 0.000 0.000 -

Sequence-1453-S 0.083 0.000 0.000 -

Sequence-1454-S 0.084 0.000 0.000 -

Sequence-1455-G 0.078 0.000 0.000 -

Sequence-1456-G 0.080 0.000 0.000 -

Sequence-1457-L 0.193 0.001 0.000 -

Sequence-1458-M 0.083 0.002 0.000 -

Sequence-1459-R 0.090 0.002 0.000 -

Sequence-1460-Q 0.081 0.002 0.000 -

Sequence-1461-C 0.113 0.002 0.000 -

Sequence-1462-L 0.161 0.003 0.000 -

Sequence-1463-R 0.070 0.003 0.000 -

Sequence-1464-L 0.467 0.003 0.200 -

Sequence-1465-V 0.080 0.002 0.000 -

Sequence-1466-C 0.131 0.001 0.000 -

Sequence-1467-C 0.148 0.001 0.000 -

Sequence-1468-V 0.088 0.001 0.000 -

Sequence-1469-A 0.091 0.001 0.000 -

Sequence-1470-V 0.131 0.001 0.000 -

Sequence-1471-R 0.095 0.000 0.000 -

Sequence-1472-N 0.091 0.000 0.000 -

Sequence-1473-C 0.090 0.000 0.000 -

Sequence-1474-L 0.086 0.000 0.000 -

Sequence-1475-E 0.074 0.000 0.000 -

Sequence-1476-C 0.088 0.000 0.000 -

Sequence-1477-R 0.085 0.000 0.000 -

Sequence-1478-Q 0.071 0.000 0.000 -

Sequence-1479-R 0.118 0.000 0.000 -

Sequence-1480-Q 0.076 0.000 0.000 -

Sequence-1481-R 0.091 0.000 0.000 -

Sequence-1482-D 0.084 0.000 0.000 -

Sequence-1483-R 0.098 0.000 0.000 -

Sequence-1484-G 0.088 0.000 0.000 -

Sequence-1485-S 0.073 0.000 0.000 -

Sequence-1486-K 0.078 0.000 0.000 -

Sequence-1487-S 0.075 0.000 0.000 -

Sequence-1488-S 0.071 0.000 0.000 -

Sequence-1489-H 0.090 0.000 0.000 -

Sequence-1490-G 0.080 0.000 0.000 -

Sequence-1491-S 0.071 0.000 0.000 -

Sequence-1492-S 0.078 0.000 0.000 -

Sequence-1493-K 0.068 0.000 0.000 -

Sequence-1494-P 0.073 0.000 0.000 -

Sequence-1495-Q 0.067 0.000 0.000 -

Sequence-1496-E 0.084 0.000 0.000 -

Sequence-1497-A 0.067 0.000 0.000 -

Sequence-1498-P 0.074 0.000 0.000 -

Sequence-1499-H 0.079 0.000 0.000 -

Sequence-1500-S 0.075 0.000 0.000 -

Sequence-1501-V 0.083 0.000 0.000 -

Sequence-1502-T 0.069 0.000 0.000 -

Sequence-1503-A 0.119 0.000 0.000 -

Sequence-1504-A 0.071 0.000 0.000 -

Sequence-1505-S 0.074 0.000 0.000 -

Sequence-1506-A 0.072 0.000 0.000 -

Sequence-1507-S 0.089 0.000 0.000 -

Sequence-1508-K 0.069 0.000 0.000 -

Sequence-1509-T 0.079 0.000 0.000 -

Sequence-1510-P 0.077 0.000 0.000 -

Sequence-1511-L 0.092 0.000 0.000 -

Sequence-1512-E 0.076 0.000 0.000 -

Sequence-1513-N 0.078 0.000 0.000 -

Sequence-1514-V 0.099 0.000 0.000 -

Sequence-1515-P 0.071 0.000 0.000 -

Sequence-1516-G 0.103 0.000 0.000 -

Sequence-1517-N 0.076 0.000 0.000 -

Sequence-1518-L 0.089 0.002 0.000 -

Sequence-1519-S 0.086 0.002 0.000 -

Sequence-1520-P 0.123 0.002 0.000 -

Sequence-1521-I 0.122 0.006 0.000 -

Sequence-1522-K 0.073 0.007 0.000 -

Sequence-1523-D 0.074 0.007 0.000 -

Sequence-1524-P 0.078 0.007 0.000 -

Sequence-1525-D 0.068 0.007 0.000 -

Sequence-1526-R 0.084 0.007 0.000 -

Sequence-1527-L 0.099 0.008 0.000 -

Sequence-1528-L 0.173 0.009 0.000 -

Sequence-1529-Q 0.155 0.009 0.000 -

Sequence-1530-D 0.121 0.009 0.000 -

Sequence-1531-V 0.093 0.009 0.000 -

Sequence-1532-D 0.080 0.009 0.000 -

Sequence-1533-I 0.758 0.009 0.300 -

Sequence-1534-N 0.073 0.002 0.000 -

Sequence-1535-R 0.092 0.002 0.000 -

Sequence-1536-L 0.377 0.002 0.000 -

Sequence-1537-R 0.087 0.001 0.000 -

Sequence-1538-A 0.201 0.001 0.000 -

Sequence-1539-V 0.084 0.001 0.000 -

Sequence-1540-V 0.083 0.000 0.000 -

Sequence-1541-F 0.086 0.000 0.000 -

Sequence-1542-R 0.106 0.000 0.000 -

Sequence-1543-D 0.086 0.000 0.000 -

Sequence-1544-V 0.098 0.002 0.000 -

Sequence-1545-D 0.070 0.002 0.000 -

Sequence-1546-D 0.150 0.002 0.000 -

Sequence-1547-S 0.068 0.002 0.000 -

Sequence-1548-K 0.075 0.003 0.000 -

Sequence-1549-Q 0.069 0.003 0.000 -

Sequence-1550-A 0.074 0.003 0.000 -

Sequence-1551-Q 0.087 0.003 0.000 -

Sequence-1552-F 0.078 0.003 0.000 -

Sequence-1553-L 0.179 0.004 0.000 -

Sequence-1554-A 0.072 0.004 0.000 -

Sequence-1555-L 0.521 0.004 0.216 -

Sequence-1556-A 0.069 0.003 0.000 -

Sequence-1557-V 0.277 0.003 0.000 -

Sequence-1558-V 0.098 0.000 0.000 -

Sequence-1559-Y 0.075 0.000 0.000 -

Sequence-1560-F 0.172 0.000 0.000 -

Sequence-1561-I 0.134 0.000 0.000 -

Sequence-1562-S 0.094 0.000 0.000 -

Sequence-1563-V 0.113 0.000 0.000 -

Sequence-1564-L 0.185 0.000 0.000 -

Sequence-1565-M 0.091 0.000 0.000 -

Sequence-1566-V 0.113 0.000 0.000 -

Sequence-1567-S 0.066 0.000 0.000 -

Sequence-1568-K 0.077 0.000 0.000 -

Sequence-1569-Y 0.103 0.000 0.000 -

Sequence-1570-R 0.077 0.000 0.000 -

Sequence-1571-D 0.092 0.000 0.000 -

Sequence-1572-I 0.151 0.000 0.000 -

Sequence-1573-L 0.100 0.000 0.000 -

Sequence-1574-E 0.130 0.000 0.000 -

Sequence-1575-P 0.094 0.000 0.000 -

Sequence-1576-Q 0.073 0.000 0.000 -

Sequence-1577-R 0.094 0.000 0.000 -

Sequence-1578-E 0.078 0.000 0.000 -

Sequence-1579-T 0.084 0.000 0.000 -

Sequence-1580-A 0.084 0.000 0.000 -

Sequence-1581-R 0.086 0.000 0.000 -

Sequence-1582-T 0.082 0.000 0.000 -

Sequence-1583-G 0.091 0.000 0.000 -

Sequence-1584-S 0.077 0.000 0.000 -

Sequence-1585-Q 0.075 0.000 0.000 -

Sequence-1586-P 0.088 0.000 0.000 -

Sequence-1587-G 0.071 0.000 0.000 -

Sequence-1588-R 0.065 0.000 0.000 -

Sequence-1589-N 0.073 0.000 0.000 -

Sequence-1590-I 0.118 0.000 0.000 -

Sequence-1591-R 0.077 0.000 0.000 -

Sequence-1592-Q 0.094 0.000 0.000 -

Sequence-1593-E 0.073 0.000 0.000 -

Sequence-1594-I 0.125 0.000 0.000 -

Sequence-1595-N 0.075 0.000 0.000 -

Sequence-1596-S 0.076 0.000 0.000 -

Sequence-1597-P 0.072 0.000 0.000 -

Sequence-1598-T 0.073 0.000 0.000 -

Sequence-1599-S 0.069 0.000 0.000 -

Sequence-1600-T 0.070 0.000 0.000 -

Sequence-1601-V 0.071 0.000 0.000 -

Sequence-1602-V 0.066 0.000 0.000 -

Sequence-1603-V 0.083 0.000 0.000 -

Sequence-1604-I 0.077 0.000 0.000 -

Sequence-1605-P 0.080 0.000 0.000 -

Sequence-1606-S 0.072 0.000 0.000 -

Sequence-1607-I 0.075 0.000 0.000 -

Sequence-1608-P 0.073 0.000 0.000 -

Sequence-1609-H 0.092 0.000 0.000 -

Sequence-1610-P 0.089 0.000 0.000 -

Sequence-1611-S 0.067 0.000 0.000 -

Sequence-1612-L 0.117 0.000 0.000 -

Sequence-1613-N 0.078 0.000 0.000 -

Sequence-1614-H 0.086 0.000 0.000 -

Sequence-1615-G 0.074 0.000 0.000 -

Sequence-1616-L 0.115 0.000 0.000 -

Sequence-1617-L 0.131 0.000 0.000 -

Sequence-1618-A 0.082 0.000 0.000 -

Sequence-1619-K 0.097 0.000 0.000 -

Sequence-1620-L 0.120 0.000 0.000 -

Sequence-1621-M 0.124 0.000 0.000 -

Sequence-1622-P 0.363 0.000 0.000 -

Sequence-1623-E 0.076 0.000 0.000 -

Sequence-1624-Q 0.086 0.000 0.000 -

Sequence-1625-S 0.105 0.000 0.000 -

Sequence-1626-F 0.113 0.000 0.000 -

Sequence-1627-A 0.076 0.000 0.000 -

Sequence-1628-H 0.080 0.000 0.000 -

Sequence-1629-S 0.088 0.000 0.000 -

Sequence-1630-F 0.078 0.000 0.000 -

Sequence-1631-Y 0.096 0.000 0.000 -

Sequence-1632-K 0.071 0.000 0.000 -

Sequence-1633-E 0.078 0.000 0.000 -

Sequence-1634-T 0.068 0.000 0.000 -

Sequence-1635-P 0.121 0.000 0.000 -

Sequence-1636-A 0.085 0.000 0.000 -

Sequence-1637-T 0.073 0.000 0.000 -

Sequence-1638-F 0.079 0.000 0.000 -

Sequence-1639-P 0.088 0.000 0.000 -

Sequence-1640-D 0.068 0.000 0.000 -

Sequence-1641-T 0.079 0.000 0.000 -

Sequence-1642-V 0.082 0.000 0.000 -

Sequence-1643-K 0.089 0.000 0.000 -

Sequence-1644-E 0.117 0.000 0.000 -

Sequence-1645-K 0.093 0.000 0.000 -

Sequence-1646-E 0.070 0.000 0.000 -

Sequence-1647-T 0.080 0.000 0.000 -

Sequence-1648-P 0.087 0.000 0.000 -

Sequence-1649-T 0.084 0.000 0.000 -

Sequence-1650-P 0.104 0.000 0.000 -

Sequence-1651-G 0.073 0.000 0.000 -

Sequence-1652-E 0.074 0.000 0.000 -

Sequence-1653-D 0.081 0.000 0.000 -

Sequence-1654-I 0.241 0.000 0.000 -

Sequence-1655-Q 0.081 0.000 0.000 -

Sequence-1656-L 0.253 0.000 0.000 -

Sequence-1657-E 0.094 0.000 0.000 -

Sequence-1658-S 0.087 0.000 0.000 -

Sequence-1659-S 0.098 0.000 0.000 -

Sequence-1660-V 0.092 0.000 0.000 -

Sequence-1661-P 0.090 0.000 0.000 -

Sequence-1662-H 0.085 0.000 0.000 -

Sequence-1663-T 0.084 0.000 0.000 -

Sequence-1664-D 0.074 0.000 0.000 -

Sequence-1665-S 0.108 0.000 0.000 -

Sequence-1666-G 0.076 0.000 0.000 -

Sequence-1667-M 0.096 0.000 0.000 -

Sequence-1668-G 0.075 0.000 0.000 -

Sequence-1669-E 0.081 0.000 0.000 -

Sequence-1670-E 0.079 0.000 0.000 -

Sequence-1671-Q 0.074 0.000 0.000 -

Sequence-1672-V 0.106 0.000 0.000 -

Sequence-1673-A 0.076 0.000 0.000 -

Sequence-1674-S 0.073 0.000 0.000 -

Sequence-1675-I 0.114 0.000 0.000 -

Sequence-1676-L 0.096 0.000 0.000 -

Sequence-1677-D 0.080 0.000 0.000 -

Sequence-1678-G 0.108 0.000 0.000 -

Sequence-1679-A 0.070 0.000 0.000 -

Sequence-1680-E 0.102 0.000 0.000 -

Sequence-1681-L 0.699 0.000 0.245 -

Sequence-1682-E 0.076 0.000 0.000 -

Sequence-1683-P 0.148 0.000 0.000 -

Sequence-1684-A 0.105 0.000 0.000 -

Sequence-1685-A 0.117 0.000 0.000 -

Sequence-1686-G 0.093 0.000 0.000 -

Sequence-1687-P 0.069 0.000 0.000 -

Sequence-1688-D 0.069 0.000 0.000 -

Sequence-1689-A 0.072 0.000 0.000 -

Sequence-1690-M 0.096 0.000 0.000 -

Sequence-1691-S 0.088 0.000 0.000 -

Sequence-1692-E 0.087 0.000 0.000 -

Sequence-1693-L 0.181 0.000 0.000 -

Sequence-1694-L 0.090 0.001 0.000 -

Sequence-1695-S 0.088 0.000 0.000 -

Sequence-1696-T 0.108 0.000 0.000 -

Sequence-1697-L 0.151 0.000 0.000 -

Sequence-1698-S 0.087 0.000 0.000 -

Sequence-1699-S 0.572 0.000 0.231 -

Sequence-1700-E 0.078 0.000 0.000 -

Sequence-1701-V 0.136 0.000 0.000 -

Sequence-1702-K 0.114 0.000 0.000 -

Sequence-1703-K 0.139 0.000 0.000 -

Sequence-1704-S 0.076 0.000 0.000 -

Sequence-1705-Q 0.083 0.000 0.000 -

Sequence-1706-E 0.110 0.000 0.000 -

Sequence-1707-S 0.074 0.000 0.000 -

Sequence-1708-L 0.127 0.000 0.000 -

Sequence-1709-T 0.079 0.000 0.000 -

Sequence-1710-E 0.098 0.000 0.000 -

Sequence-1711-H 0.081 0.000 0.000 -

Sequence-1712-P 0.085 0.000 0.000 -

Sequence-1713-S 0.117 0.000 0.000 -

Sequence-1714-E 0.089 0.000 0.000 -

Sequence-1715-M 0.094 0.000 0.000 -

Sequence-1716-L 0.204 0.000 0.000 -

Sequence-1717-K 0.157 0.000 0.000 -

Sequence-1718-P 0.093 0.000 0.000 -

Sequence-1719-A 0.072 0.000 0.000 -

Sequence-1720-P 0.087 0.000 0.000 -

Sequence-1721-S 0.088 0.000 0.000 -

Sequence-1722-I 0.115 0.000 0.000 -

Sequence-1723-S 0.071 0.000 0.000 -

Sequence-1724-S 0.108 0.000 0.000 -

Sequence-1725-I 0.195 0.000 0.000 -

Sequence-1726-S 0.070 0.000 0.000 -

Sequence-1727-Q 0.080 0.000 0.000 -

Sequence-1728-T 0.085 0.000 0.000 -

Sequence-1729-K 0.074 0.000 0.000 -

Sequence-1730-G 0.071 0.000 0.000 -

Sequence-1731-I 0.079 0.000 0.000 -

Sequence-1732-N 0.073 0.000 0.000 -

Sequence-1733-V 0.091 0.000 0.000 -

Sequence-1734-K 0.072 0.000 0.000 -

Sequence-1735-E 0.076 0.000 0.000 -

Sequence-1736-I 0.100 0.000 0.000 -

Sequence-1737-L 0.093 0.000 0.000 -

Sequence-1738-K 0.077 0.000 0.000 -

Sequence-1739-S 0.092 0.000 0.000 -

Sequence-1740-L 0.121 0.000 0.000 -

Sequence-1741-V 0.133 0.000 0.000 -

Sequence-1742-A 0.127 0.000 0.000 -

Sequence-1743-A 0.075 0.000 0.000 -

Sequence-1744-P 0.080 0.000 0.000 -

Sequence-1745-V 0.155 0.000 0.000 -

Sequence-1746-E 0.088 0.000 0.000 -

Sequence-1747-I 0.141 0.000 0.000 -

Sequence-1748-A 0.079 0.000 0.000 -

Sequence-1749-E 0.086 0.000 0.000 -

Sequence-1750-C 0.081 0.000 0.000 -

Sequence-1751-G 0.067 0.000 0.000 -

Sequence-1752-P 0.069 0.000 0.000 -

Sequence-1753-E 0.077 0.000 0.000 -

Sequence-1754-P 0.068 0.000 0.000 -

Sequence-1755-I 0.082 0.000 0.000 -

Sequence-1756-P 0.073 0.000 0.000 -

Sequence-1757-Y 0.073 0.000 0.000 -

Sequence-1758-P 0.080 0.000 0.000 -

Sequence-1759-D 0.069 0.000 0.000 -

Sequence-1760-P 0.086 0.000 0.000 -

Sequence-1761-A 0.068 0.000 0.000 -

Sequence-1762-L 0.111 0.000 0.000 -

Sequence-1763-K 0.075 0.000 0.000 -

Sequence-1764-R 0.115 0.000 0.000 -

Sequence-1765-E 0.075 0.000 0.000 -

Sequence-1766-A 0.084 0.000 0.000 -

Sequence-1767-H 0.138 0.000 0.000 -

Sequence-1768-A 0.069 0.000 0.000 -

Sequence-1769-I 0.168 0.000 0.000 -

Sequence-1770-L 0.133 0.000 0.000 -

Sequence-1771-P 0.087 0.000 0.000 -

Sequence-1772-M 0.356 0.000 0.000 -

Sequence-1773-Q 0.069 0.000 0.000 -

Sequence-1774-F 0.150 0.000 0.000 -

Sequence-1775-H 0.084 0.000 0.000 -

Sequence-1776-S 0.080 0.000 0.000 -

Sequence-1777-F 0.092 0.000 0.000 -

Sequence-1778-D 0.077 0.000 0.000 -

Sequence-1779-R 0.098 0.000 0.000 -

Sequence-1780-S 0.084 0.000 0.000 -

Sequence-1781-V 0.074 0.000 0.000 -

Sequence-1782-V 0.080 0.000 0.000 -

Sequence-1783-V 0.079 0.000 0.000 -

Sequence-1784-P 0.070 0.000 0.000 -

Sequence-1785-V 0.079 0.000 0.000 -

Sequence-1786-K 0.085 0.000 0.000 -

Sequence-1787-K 0.069 0.000 0.000 -

Sequence-1788-P 0.075 0.000 0.000 -

Sequence-1789-P 0.073 0.000 0.000 -

Sequence-1790-P 0.076 0.000 0.000 -

Sequence-1791-G 0.068 0.000 0.000 -

Sequence-1792-S 0.064 0.000 0.000 -

Sequence-1793-L 0.095 0.000 0.000 -

Sequence-1794-A 0.074 0.000 0.000 -

Sequence-1795-V 0.100 0.000 0.000 -

Sequence-1796-T 0.068 0.000 0.000 -

Sequence-1797-T 0.095 0.000 0.000 -

Sequence-1798-V 0.124 0.000 0.000 -

Sequence-1799-G 0.071 0.000 0.000 -

Sequence-1800-A 0.076 0.000 0.000 -

Sequence-1801-T 0.070 0.000 0.000 -

Sequence-1802-A 0.074 0.000 0.000 -

Sequence-1803-A 0.080 0.000 0.000 -

Sequence-1804-G 0.082 0.000 0.000 -

Sequence-1805-S 0.066 0.000 0.000 -

Sequence-1806-G 0.081 0.000 0.000 -

Sequence-1807-L 0.110 0.000 0.000 -

Sequence-1808-P 0.076 0.000 0.000 -

Sequence-1809-T 0.148 0.000 0.000 -

Sequence-1810-G 0.072 0.000 0.000 -

Sequence-1811-S 0.069 0.000 0.000 -

Sequence-1812-T 0.084 0.000 0.000 -

Sequence-1813-S 0.070 0.000 0.000 -

Sequence-1814-S 0.077 0.000 0.000 -

Sequence-1815-I 0.082 0.000 0.000 -

Sequence-1816-F 0.087 0.000 0.000 -

Sequence-1817-A 0.103 0.000 0.000 -

Sequence-1818-A 0.075 0.000 0.000 -

Sequence-1819-P 0.070 0.000 0.000 -

Sequence-1820-G 0.069 0.000 0.000 -

Sequence-1821-A 0.071 0.000 0.000 -

Sequence-1822-T 0.075 0.000 0.000 -

Sequence-1823-P 0.088 0.000 0.000 -

Sequence-1824-K 0.089 0.000 0.000 -

Sequence-1825-S 0.071 0.000 0.000 -

Sequence-1826-M 0.069 0.000 0.000 -

Sequence-1827-I 0.095 0.000 0.000 -

Sequence-1828-N 0.074 0.000 0.000 -

Sequence-1829-T 0.069 0.000 0.000 -

Sequence-1830-T 0.075 0.000 0.000 -

Sequence-1831-G 0.079 0.000 0.000 -

Sequence-1832-A 0.074 0.000 0.000 -

Sequence-1833-V 0.078 0.000 0.000 -

Sequence-1834-D 0.074 0.000 0.000 -

Sequence-1835-S 0.092 0.000 0.000 -

Sequence-1836-G 0.080 0.000 0.000 -

Sequence-1837-S 0.076 0.000 0.000 -

Sequence-1838-S 0.078 0.000 0.000 -

Sequence-1839-S 0.070 0.000 0.000 -

Sequence-1840-S 0.070 0.000 0.000 -

Sequence-1841-S 0.070 0.000 0.000 -

Sequence-1842-S 0.076 0.000 0.000 -

Sequence-1843-S 0.070 0.000 0.000 -

Sequence-1844-S 0.078 0.000 0.000 -

Sequence-1845-S 0.070 0.000 0.000 -

Sequence-1846-F 0.078 0.000 0.000 -

Sequence-1847-V 0.081 0.000 0.000 -

Sequence-1848-N 0.077 0.000 0.000 -

Sequence-1849-G 0.088 0.000 0.000 -

Sequence-1850-A 0.069 0.000 0.000 -

Sequence-1851-T 0.086 0.000 0.000 -

Sequence-1852-S 0.082 0.000 0.000 -

Sequence-1853-K 0.081 0.000 0.000 -

Sequence-1854-N 0.073 0.000 0.000 -

Sequence-1855-L 0.086 0.000 0.000 -

Sequence-1856-P 0.078 0.000 0.000 -

Sequence-1857-A 0.070 0.000 0.000 -

Sequence-1858-V 0.082 0.000 0.000 -

Sequence-1859-Q 0.070 0.000 0.000 -

Sequence-1860-T 0.080 0.000 0.000 -

Sequence-1861-V 0.084 0.000 0.000 -

Sequence-1862-A 0.070 0.000 0.000 -

Sequence-1863-P 0.137 0.000 0.000 -

Sequence-1864-M 0.082 0.000 0.000 -

Sequence-1865-P 0.077 0.000 0.000 -

Sequence-1866-E 0.072 0.000 0.000 -

Sequence-1867-D 0.071 0.000 0.000 -

Sequence-1868-S 0.072 0.000 0.000 -

Sequence-1869-A 0.068 0.000 0.000 -

Sequence-1870-E 0.104 0.000 0.000 -

Sequence-1871-N 0.069 0.000 0.000 -

Sequence-1872-M 0.099 0.000 0.000 -

Sequence-1873-S 0.078 0.000 0.000 -

Sequence-1874-I 0.143 0.000 0.000 -

Sequence-1875-T 0.074 0.000 0.000 -

Sequence-1876-A 0.082 0.000 0.000 -

Sequence-1877-K 0.119 0.000 0.000 -

Sequence-1878-L 0.079 0.002 0.000 -

Sequence-1879-E 0.071 0.002 0.000 -

Sequence-1880-R 0.152 0.002 0.000 -

Sequence-1881-A 0.094 0.002 0.000 -

Sequence-1882-L 0.433 0.004 0.193 -

Sequence-1883-E 0.114 0.004 0.000 -

Sequence-1884-K 0.148 0.004 0.000 -

Sequence-1885-V 0.087 0.004 0.000 -

Sequence-1886-A 0.089 0.004 0.000 -

Sequence-1887-P 0.273 0.004 0.000 -

Sequence-1888-L 0.098 0.004 0.000 -

Sequence-1889-L 0.100 0.004 0.000 -

Sequence-1890-R 0.097 0.004 0.000 -

Sequence-1891-E 0.171 0.004 0.000 -

Sequence-1892-I 0.131 0.004 0.000 -

Sequence-1893-F 0.119 0.004 0.000 -

Sequence-1894-V 0.144 0.003 0.000 -

Sequence-1895-D 0.074 0.001 0.000 -

Sequence-1896-F 0.106 0.001 0.000 -

Sequence-1897-A 0.094 0.000 0.000 -

Sequence-1898-P 0.105 0.000 0.000 -

Sequence-1899-F 0.073 0.000 0.000 -

Sequence-1900-L 0.088 0.001 0.000 -

Sequence-1901-S 0.090 0.001 0.000 -

Sequence-1902-R 0.090 0.001 0.000 -

Sequence-1903-T 0.074 0.001 0.000 -

Sequence-1904-L 0.123 0.001 0.000 -

Sequence-1905-L 0.400 0.001 0.000 -

Sequence-1906-G 0.073 0.001 0.000 -

Sequence-1907-S 0.093 0.001 0.000 -

Sequence-1908-H 0.117 0.001 0.000 -

Sequence-1909-G 0.098 0.001 0.000 -

Sequence-1910-Q 0.159 0.001 0.000 -

Sequence-1911-E 0.112 0.001 0.000 -

Sequence-1912-L 0.090 0.003 0.000 -

Sequence-1913-L 0.133 0.003 0.000 -

Sequence-1914-I 0.500 0.003 0.231 -

Sequence-1915-E 0.110 0.002 0.000 -

Sequence-1916-G 0.076 0.002 0.000 -

Sequence-1917-L 0.177 0.002 0.000 -

Sequence-1918-V 0.102 0.002 0.000 -

Sequence-1919-C 0.160 0.002 0.000 -

Sequence-1920-M 0.105 0.002 0.000 -

Sequence-1921-K 0.159 0.000 0.000 -

Sequence-1922-S 0.328 0.000 0.000 -

Sequence-1923-S 0.082 0.000 0.000 -

Sequence-1924-T 0.074 0.000 0.000 -

Sequence-1925-S 0.081 0.000 0.000 -

Sequence-1926-V 0.081 0.001 0.000 -

Sequence-1927-V 0.078 0.004 0.000 -

Sequence-1928-E 0.068 0.004 0.000 -

Sequence-1929-L 0.191 0.004 0.000 -

Sequence-1930-V 0.071 0.004 0.000 -

Sequence-1931-M 0.104 0.004 0.000 -

Sequence-1932-L 0.305 0.004 0.000 -

Sequence-1933-L 0.150 0.004 0.000 -

Sequence-1934-C 0.162 0.000 0.000 -

Sequence-1935-S 0.172 0.000 0.000 -

Sequence-1936-Q 0.112 0.000 0.000 -

Sequence-1937-E 0.254 0.000 0.000 -

Sequence-1938-W 0.151 0.000 0.000 -

Sequence-1939-Q 0.113 0.000 0.000 -

Sequence-1940-N 0.082 0.000 0.000 -

Sequence-1941-S 0.089 0.000 0.000 -

Sequence-1942-I 0.113 0.000 0.000 -

Sequence-1943-Q 0.072 0.000 0.000 -

Sequence-1944-K 0.068 0.000 0.000 -

Sequence-1945-N 0.076 0.000 0.000 -

Sequence-1946-A 0.066 0.000 0.000 -

Sequence-1947-G 0.071 0.000 0.000 -

Sequence-1948-L 0.102 0.000 0.000 -

Sequence-1949-A 0.068 0.000 0.000 -

Sequence-1950-F 0.185 0.000 0.000 -

Sequence-1951-I 0.126 0.000 0.000 -

Sequence-1952-E 0.082 0.000 0.000 -

Sequence-1953-L 0.359 0.000 0.000 -

Sequence-1954-I 0.082 0.000 0.000 -

Sequence-1955-N 0.139 0.000 0.000 -

Sequence-1956-E 0.082 0.000 0.000 -

Sequence-1957-G 0.081 0.000 0.000 -

Sequence-1958-R 0.095 0.000 0.000 -

Sequence-1959-L 0.164 0.000 0.000 -

Sequence-1960-L 0.215 0.000 0.000 -

Sequence-1961-C 0.113 0.000 0.000 -

Sequence-1962-H 0.128 0.000 0.000 -

Sequence-1963-A 0.097 0.000 0.000 -

Sequence-1964-M 0.174 0.000 0.000 -

Sequence-1965-K 0.074 0.000 0.000 -

Sequence-1966-D 0.073 0.000 0.000 -

Sequence-1967-H 0.077 0.000 0.000 -

Sequence-1968-I 0.176 0.000 0.000 -

Sequence-1969-V 0.136 0.000 0.000 -

Sequence-1970-R 0.075 0.000 0.000 -

Sequence-1971-V 0.091 0.000 0.000 -

Sequence-1972-A 0.080 0.000 0.000 -

Sequence-1973-N 0.077 0.000 0.000 -

Sequence-1974-E 0.075 0.000 0.000 -

Sequence-1975-A 0.078 0.000 0.000 -

Sequence-1976-E 0.079 0.000 0.000 -

Sequence-1977-F 0.074 0.000 0.000 -

Sequence-1978-I 0.141 0.000 0.000 -

Sequence-1979-L 0.100 0.000 0.000 -

Sequence-1980-N 0.078 0.000 0.000 -

Sequence-1981-R 0.080 0.000 0.000 -

Sequence-1982-Q 0.077 0.000 0.000 -

Sequence-1983-R 0.123 0.000 0.000 -

Sequence-1984-A 0.083 0.000 0.000 -

Sequence-1985-E 0.078 0.000 0.000 -

Sequence-1986-D 0.092 0.000 0.000 -

Sequence-1987-V 0.123 0.000 0.000 -

Sequence-1988-H 0.088 0.000 0.000 -

Sequence-1989-K 0.146 0.000 0.000 -

Sequence-1990-H 0.070 0.000 0.000 -

Sequence-1991-A 0.074 0.000 0.000 -

Sequence-1992-E 0.077 0.000 0.000 -

Sequence-1993-F 0.079 0.000 0.000 -

Sequence-1994-E 0.077 0.000 0.000 -

Sequence-1995-S 0.078 0.000 0.000 -

Sequence-1996-Q 0.090 0.000 0.000 -

Sequence-1997-C 0.074 0.000 0.000 -

Sequence-1998-A 0.086 0.000 0.000 -

Sequence-1999-Q 0.074 0.000 0.000 -

Sequence-2000-Y 0.085 0.000 0.000 -

Sequence-2001-A 0.074 0.000 0.000 -

Sequence-2002-A 0.077 0.000 0.000 -

Sequence-2003-D 0.080 0.000 0.000 -

Sequence-2004-R 0.074 0.000 0.000 -

Sequence-2005-R 0.099 0.000 0.000 -

Sequence-2006-E 0.091 0.000 0.000 -

Sequence-2007-E 0.082 0.000 0.000 -

Sequence-2008-E 0.086 0.000 0.000 -

Sequence-2009-K 0.081 0.000 0.000 -

Sequence-2010-M 0.101 0.000 0.000 -

Sequence-2011-C 0.090 0.000 0.000 -

Sequence-2012-D 0.092 0.000 0.000 -

Sequence-2013-H 0.085 0.000 0.000 -

Sequence-2014-L 0.195 0.000 0.000 -

Sequence-2015-I 0.439 0.000 0.100 -

Sequence-2016-S 0.086 0.000 0.000 -

Sequence-2017-A 0.105 0.000 0.000 -

Sequence-2018-A 0.113 0.000 0.000 -

Sequence-2019-K 0.103 0.000 0.000 -

Sequence-2020-H 0.098 0.000 0.000 -

Sequence-2021-R 0.075 0.000 0.000 -

Sequence-2022-D 0.082 0.000 0.000 -

Sequence-2023-H 0.118 0.000 0.000 -

Sequence-2024-V 0.090 0.000 0.000 -

Sequence-2025-T 0.090 0.000 0.000 -

Sequence-2026-A 0.096 0.000 0.000 -

Sequence-2027-N 0.070 0.000 0.000 -

Sequence-2028-Q 0.065 0.000 0.000 -

Sequence-2029-L 0.091 0.000 0.000 -

Sequence-2030-K 0.087 0.000 0.000 -

Sequence-2031-Q 0.071 0.000 0.000 -

Sequence-2032-K 0.082 0.000 0.000 -

Sequence-2033-I 0.134 0.000 0.000 -

Sequence-2034-L 0.107 0.000 0.000 -

Sequence-2035-N 0.085 0.000 0.000 -

Sequence-2036-I 0.386 0.000 0.000 -

Sequence-2037-L 0.107 0.000 0.000 -

Sequence-2038-T 0.147 0.000 0.000 -

Sequence-2039-N 0.213 0.000 0.000 -

Sequence-2040-K 0.088 0.000 0.000 -

Sequence-2041-H 0.089 0.000 0.000 -

Sequence-2042-G 0.105 0.000 0.000 -

Sequence-2043-A 0.084 0.000 0.000 -

Sequence-2044-W 0.084 0.000 0.000 -

Sequence-2045-G 0.075 0.000 0.000 -

Sequence-2046-A 0.076 0.000 0.000 -

Sequence-2047-V 0.081 0.000 0.000 -

Sequence-2048-S 0.067 0.000 0.000 -

Sequence-2049-H 0.082 0.000 0.000 -

Sequence-2050-S 0.078 0.000 0.000 -

Sequence-2051-Q 0.069 0.000 0.000 -

Sequence-2052-L 0.117 0.000 0.000 -

Sequence-2053-H 0.068 0.000 0.000 -

Sequence-2054-D 0.090 0.000 0.000 -

Sequence-2055-F 0.071 0.000 0.000 -

Sequence-2056-W 0.070 0.000 0.000 -

Sequence-2057-R 0.110 0.000 0.000 -

Sequence-2058-L 0.152 0.000 0.000 -

Sequence-2059-D 0.076 0.000 0.000 -

Sequence-2060-Y 0.357 0.000 0.000 -

Sequence-2061-W 0.100 0.000 0.000 -

Sequence-2062-E 0.142 0.000 0.000 -

Sequence-2063-D 0.081 0.000 0.000 -

Sequence-2064-D 0.077 0.000 0.000 -

Sequence-2065-L 0.122 0.000 0.000 -

Sequence-2066-R 0.080 0.000 0.000 -

Sequence-2067-R 0.191 0.000 0.000 -

Sequence-2068-R 0.079 0.000 0.000 -

Sequence-2069-R 0.115 0.000 0.000 -

Sequence-2070-R 0.109 0.000 0.000 -

Sequence-2071-F 0.102 0.000 0.000 -

Sequence-2072-V 0.158 0.000 0.000 -

Sequence-2073-R 0.093 0.000 0.000 -

Sequence-2074-N 0.102 0.000 0.000 -

Sequence-2075-A 0.084 0.000 0.000 -

Sequence-2076-F 0.078 0.000 0.000 -

Sequence-2077-G 0.081 0.000 0.000 -

Sequence-2078-S 0.069 0.000 0.000 -

Sequence-2079-T 0.068 0.000 0.000 -

Sequence-2080-H 0.081 0.000 0.000 -

Sequence-2081-A 0.076 0.000 0.000 -

Sequence-2082-E 0.070 0.000 0.000 -

Sequence-2083-A 0.075 0.000 0.000 -

Sequence-2084-L 0.080 0.000 0.000 -

Sequence-2085-L 0.098 0.000 0.000 -

Sequence-2086-K 0.084 0.000 0.000 -

Sequence-2087-S 0.103 0.000 0.000 -

Sequence-2088-A 0.076 0.000 0.000 -

Sequence-2089-V 0.358 0.000 0.000 -

Sequence-2090-E 0.086 0.000 0.000 -

Sequence-2091-Y 0.111 0.000 0.000 -

Sequence-2092-G 0.096 0.000 0.000 -

Sequence-2093-T 0.095 0.000 0.000 -

Sequence-2094-E 0.150 0.000 0.000 -

Sequence-2095-E 0.076 0.000 0.000 -

Sequence-2096-D 0.078 0.000 0.000 -

Sequence-2097-V 0.076 0.000 0.000 -

Sequence-2098-V 0.085 0.000 0.000 -

Sequence-2099-K 0.080 0.000 0.000 -

Sequence-2100-S 0.080 0.000 0.000 -

Sequence-2101-K 0.094 0.000 0.000 -

Sequence-2102-K 0.076 0.000 0.000 -

Sequence-2103-A 0.075 0.000 0.000 -

Sequence-2104-F 0.091 0.000 0.000 -

Sequence-2105-R 0.088 0.000 0.000 -

Sequence-2106-S 0.094 0.000 0.000 -

Sequence-2107-Q 0.076 0.000 0.000 -

Sequence-2108-A 0.080 0.000 0.000 -

Sequence-2109-I 0.328 0.000 0.000 -

Sequence-2110-V 0.086 0.000 0.000 -

Sequence-2111-N 0.072 0.000 0.000 -

Sequence-2112-Q 0.076 0.000 0.000 -

Sequence-2113-N 0.082 0.000 0.000 -

Sequence-2114-S 0.080 0.000 0.000 -

Sequence-2115-E 0.069 0.000 0.000 -

Sequence-2116-T 0.087 0.000 0.000 -

Sequence-2117-E 0.076 0.000 0.000 -

Sequence-2118-L 0.134 0.013 0.000 -

Sequence-2119-M 0.081 0.014 0.000 -

Sequence-2120-L 0.109 0.014 0.000 -

Sequence-2121-E 0.104 0.014 0.000 -

Sequence-2122-G 0.114 0.014 0.000 -

Sequence-2123-D 0.090 0.014 0.000 -

Sequence-2124-D 0.126 0.014 0.000 -

Sequence-2125-D 0.096 0.014 0.000 -

Sequence-2126-A 0.127 0.014 0.000 -

Sequence-2127-V 0.203 0.014 0.000 -

Sequence-2128-S 0.146 0.014 0.000 -

Sequence-2129-L 0.378 0.015 0.000 -

Sequence-2130-L 0.111 0.003 0.000 -

Sequence-2131-Q 0.102 0.002 0.000 -

Sequence-2132-E 0.158 0.002 0.000 -

Sequence-2133-K 0.082 0.002 0.000 -

Sequence-2134-E 0.102 0.002 0.000 -

Sequence-2135-I 0.441 0.002 0.201 -

Sequence-2136-D 0.085 0.002 0.000 -

Sequence-2137-N 0.090 0.002 0.000 -

Sequence-2138-L 0.313 0.002 0.000 -

Sequence-2139-A 0.092 0.000 0.000 -

Sequence-2140-G 0.152 0.000 0.000 -

Sequence-2141-P 0.067 0.000 0.000 -

Sequence-2142-V 0.089 0.000 0.000 -

Sequence-2143-V 0.115 0.000 0.000 -

Sequence-2144-L 0.185 0.000 0.000 -

Sequence-2145-S 0.078 0.000 0.000 -

Sequence-2146-T 0.089 0.000 0.000 -

Sequence-2147-P 0.086 0.000 0.000 -

Sequence-2148-A 0.073 0.000 0.000 -

Sequence-2149-Q 0.071 0.000 0.000 -

Sequence-2150-L 0.087 0.000 0.000 -

Sequence-2151-I 0.080 0.001 0.000 -

Sequence-2152-A 0.079 0.001 0.000 -

Sequence-2153-P 0.103 0.001 0.000 -

Sequence-2154-V 0.125 0.002 0.000 -

Sequence-2155-V 0.072 0.002 0.000 -

Sequence-2156-V 0.092 0.002 0.000 -

Sequence-2157-A 0.073 0.002 0.000 -

Sequence-2158-K 0.088 0.002 0.000 -

Sequence-2159-G 0.071 0.002 0.000 -

Sequence-2160-T 0.086 0.002 0.000 -

Sequence-2161-L 0.131 0.002 0.000 -

Sequence-2162-S 0.074 0.002 0.000 -

Sequence-2163-I 0.160 0.002 0.000 -

Sequence-2164-T 0.068 0.000 0.000 -

Sequence-2165-T 0.087 0.000 0.000 -

Sequence-2166-T 0.084 0.000 0.000 -

Sequence-2167-E 0.081 0.000 0.000 -

Sequence-2168-I 0.093 0.000 0.000 -

Sequence-2169-Y 0.074 0.000 0.000 -

Sequence-2170-F 0.089 0.000 0.000 -

Sequence-2171-E 0.120 0.000 0.000 -

Sequence-2172-V 0.156 0.000 0.000 -

Sequence-2173-D 0.082 0.000 0.000 -

Sequence-2174-E 0.097 0.000 0.000 -

Sequence-2175-D 0.078 0.000 0.000 -

Sequence-2176-D 0.096 0.000 0.000 -

Sequence-2177-A 0.095 0.000 0.000 -

Sequence-2178-A 0.111 0.000 0.000 -

Sequence-2179-F 0.094 0.000 0.000 -

Sequence-2180-K 0.074 0.000 0.000 -

Sequence-2181-K 0.092 0.000 0.000 -

Sequence-2182-I 0.100 0.000 0.000 -

Sequence-2183-D 0.076 0.000 0.000 -

Sequence-2184-T 0.331 0.000 0.000 -

Sequence-2185-K 0.078 0.000 0.000 -

Sequence-2186-V 0.102 0.000 0.000 -

Sequence-2187-L 0.127 0.000 0.000 -

Sequence-2188-A 0.082 0.000 0.000 -

Sequence-2189-Y 0.116 0.000 0.000 -

Sequence-2190-T 0.073 0.000 0.000 -

Sequence-2191-E 0.235 0.000 0.000 -

Sequence-2192-G 0.113 0.000 0.000 -

Sequence-2193-L 0.133 0.000 0.000 -

Sequence-2194-H 0.091 0.000 0.000 -

Sequence-2195-G 0.167 0.000 0.000 -

Sequence-2196-K 0.083 0.000 0.000 -

Sequence-2197-W 0.095 0.000 0.000 -

Sequence-2198-M 0.115 0.000 0.000 -

Sequence-2199-F 0.085 0.000 0.000 -

Sequence-2200-S 0.081 0.000 0.000 -

Sequence-2201-E 0.080 0.000 0.000 -

Sequence-2202-I 0.227 0.000 0.000 -

Sequence-2203-R 0.081 0.000 0.000 -

Sequence-2204-A 0.084 0.000 0.000 -

Sequence-2205-V 0.083 0.000 0.000 -

Sequence-2206-F 0.068 0.000 0.000 -

Sequence-2207-S 0.080 0.000 0.000 -

Sequence-2208-R 0.079 0.000 0.000 -

Sequence-2209-R 0.074 0.000 0.000 -

Sequence-2210-Y 0.081 0.000 0.000 -

Sequence-2211-L 0.540 0.000 0.196 -

Sequence-2212-L 0.129 0.000 0.000 -

Sequence-2213-Q 0.092 0.000 0.000 -

Sequence-2214-N 0.088 0.000 0.000 -

Sequence-2215-T 0.069 0.002 0.000 -

Sequence-2216-A 0.083 0.002 0.000 -

Sequence-2217-L 0.260 0.002 0.000 -

Sequence-2218-E 0.077 0.002 0.000 -

Sequence-2219-V 0.177 0.002 0.000 -

Sequence-2220-F 0.091 0.002 0.000 -

Sequence-2221-M 0.126 0.002 0.000 -

Sequence-2222-A 0.130 0.000 0.000 -

Sequence-2223-N 0.072 0.000 0.000 -

Sequence-2224-R 0.079 0.000 0.000 -

Sequence-2225-T 0.079 0.000 0.000 -

Sequence-2226-S 0.083 0.000 0.000 -

Sequence-2227-V 0.088 0.000 0.000 -

Sequence-2228-M 0.076 0.000 0.000 -

Sequence-2229-F 0.091 0.000 0.000 -

Sequence-2230-N 0.109 0.000 0.000 -

Sequence-2231-F 0.071 0.000 0.000 -

Sequence-2232-P 0.079 0.000 0.000 -

Sequence-2233-D 0.075 0.000 0.000 -

Sequence-2234-Q 0.068 0.000 0.000 -

Sequence-2235-A 0.071 0.000 0.000 -

Sequence-2236-T 0.090 0.000 0.000 -

Sequence-2237-V 0.079 0.000 0.000 -

Sequence-2238-K 0.077 0.000 0.000 -

Sequence-2239-K 0.069 0.000 0.000 -

Sequence-2240-V 0.076 0.000 0.000 -

Sequence-2241-V 0.085 0.000 0.000 -

Sequence-2242-Y 0.074 0.000 0.000 -

Sequence-2243-S 0.089 0.000 0.000 -

Sequence-2244-L 0.091 0.000 0.000 -

Sequence-2245-P 0.077 0.000 0.000 -

Sequence-2246-R 0.129 0.000 0.000 -

Sequence-2247-V 0.078 0.000 0.000 -

Sequence-2248-G 0.080 0.000 0.000 -

Sequence-2249-V 0.199 0.000 0.000 -

Sequence-2250-G 0.069 0.000 0.000 -

Sequence-2251-T 0.074 0.000 0.000 -

Sequence-2252-S 0.099 0.000 0.000 -

Sequence-2253-Y 0.078 0.000 0.000 -

Sequence-2254-G 0.098 0.000 0.000 -

Sequence-2255-L 0.079 0.002 0.000 -

Sequence-2256-P 0.070 0.002 0.000 -

Sequence-2257-Q 0.069 0.002 0.000 -

Sequence-2258-A 0.071 0.002 0.000 -

Sequence-2259-R 0.075 0.002 0.000 -

Sequence-2260-R 0.074 0.002 0.000 -

Sequence-2261-I 0.106 0.002 0.000 -

Sequence-2262-S 0.076 0.002 0.000 -

Sequence-2263-L 0.207 0.002 0.000 -

Sequence-2264-A 0.077 0.000 0.000 -

Sequence-2265-T 0.123 0.000 0.000 -

Sequence-2266-P 0.080 0.000 0.000 -

Sequence-2267-R 0.065 0.000 0.000 -

Sequence-2268-Q 0.100 0.000 0.000 -

Sequence-2269-L 0.090 0.000 0.000 -

Sequence-2270-Y 0.085 0.000 0.000 -

Sequence-2271-K 0.104 0.000 0.000 -

Sequence-2272-S 0.089 0.000 0.000 -

Sequence-2273-S 0.076 0.000 0.000 -

Sequence-2274-N 0.076 0.000 0.000 -

Sequence-2275-M 0.078 0.000 0.000 -

Sequence-2276-T 0.069 0.000 0.000 -

Sequence-2277-Q 0.083 0.000 0.000 -

Sequence-2278-R 0.084 0.000 0.000 -

Sequence-2279-W 0.076 0.000 0.000 -

Sequence-2280-Q 0.068 0.000 0.000 -

Sequence-2281-R 0.086 0.000 0.000 -

Sequence-2282-R 0.087 0.000 0.000 -

Sequence-2283-E 0.078 0.000 0.000 -

Sequence-2284-I 0.273 0.000 0.000 -

Sequence-2285-S 0.068 0.000 0.000 -

Sequence-2286-N 0.078 0.000 0.000 -

Sequence-2287-F 0.105 0.001 0.000 -

Sequence-2288-E 0.068 0.001 0.000 -

Sequence-2289-Y 0.109 0.001 0.000 -

Sequence-2290-L 0.088 0.001 0.000 -

Sequence-2291-M 0.076 0.001 0.000 -

Sequence-2292-F 0.141 0.001 0.000 -

Sequence-2293-L 0.173 0.001 0.000 -

Sequence-2294-N 0.084 0.000 0.000 -

Sequence-2295-T 0.121 0.000 0.000 -

Sequence-2296-I 0.119 0.000 0.000 -

Sequence-2297-A 0.086 0.000 0.000 -

Sequence-2298-G 0.098 0.000 0.000 -

Sequence-2299-R 0.120 0.000 0.000 -

Sequence-2300-T 0.081 0.000 0.000 -

Sequence-2301-Y 0.110 0.000 0.000 -

Sequence-2302-N 0.082 0.000 0.000 -

Sequence-2303-D 0.080 0.000 0.000 -

Sequence-2304-L 0.101 0.000 0.000 -

Sequence-2305-N 0.075 0.000 0.000 -

Sequence-2306-Q 0.075 0.000 0.000 -

Sequence-2307-Y 0.071 0.000 0.000 -

Sequence-2308-P 0.068 0.000 0.000 -

Sequence-2309-V 0.070 0.000 0.000 -

Sequence-2310-F 0.070 0.000 0.000 -

Sequence-2311-P 0.086 0.000 0.000 -

Sequence-2312-W 0.082 0.000 0.000 -

Sequence-2313-V 0.094 0.002 0.000 -

Sequence-2314-L 0.083 0.016 0.000 -

Sequence-2315-T 0.099 0.017 0.000 -

Sequence-2316-N 0.082 0.017 0.000 -

Sequence-2317-Y 0.081 0.017 0.000 -

Sequence-2318-E 0.070 0.017 0.000 -

Sequence-2319-S 0.083 0.017 0.000 -

Sequence-2320-E 0.090 0.017 0.000 -

Sequence-2321-E 0.085 0.017 0.000 -

Sequence-2322-L 0.306 0.018 0.000 -

Sequence-2323-D 0.071 0.018 0.000 -

Sequence-2324-L 0.211 0.019 0.000 -

Sequence-2325-T 0.070 0.018 0.000 -

Sequence-2326-L 0.774 0.018 0.337 -

Sequence-2327-P 0.091 0.001 0.000 -

Sequence-2328-G 0.139 0.001 0.000 -

Sequence-2329-N 0.173 0.001 0.000 -

Sequence-2330-F 0.096 0.001 0.000 -

Sequence-2331-R 0.301 0.001 0.000 -

Sequence-2332-D 0.117 0.001 0.000 -

Sequence-2333-L 0.352 0.001 0.000 -

Sequence-2334-S 0.073 0.000 0.000 -

Sequence-2335-K 0.150 0.000 0.000 -

Sequence-2336-P 0.078 0.000 0.000 -

Sequence-2337-I 0.108 0.000 0.000 -

Sequence-2338-G 0.073 0.000 0.000 -

Sequence-2339-A 0.074 0.000 0.000 -

Sequence-2340-L 0.103 0.000 0.000 -

Sequence-2341-N 0.069 0.000 0.000 -

Sequence-2342-P 0.200 0.000 0.000 -

Sequence-2343-K 0.068 0.000 0.000 -

Sequence-2344-R 0.076 0.000 0.000 -

Sequence-2345-A 0.083 0.000 0.000 -

Sequence-2346-V 0.104 0.000 0.000 -

Sequence-2347-F 0.077 0.000 0.000 -

Sequence-2348-Y 0.081 0.000 0.000 -

Sequence-2349-A 0.085 0.000 0.000 -

Sequence-2350-E 0.074 0.000 0.000 -

Sequence-2351-R 0.076 0.000 0.000 -

Sequence-2352-Y 0.081 0.000 0.000 -

Sequence-2353-E 0.081 0.000 0.000 -

Sequence-2354-T 0.078 0.000 0.000 -

Sequence-2355-W 0.076 0.000 0.000 -

Sequence-2356-E 0.072 0.000 0.000 -

Sequence-2357-E 0.084 0.000 0.000 -

Sequence-2358-D 0.074 0.000 0.000 -

Sequence-2359-Q 0.083 0.000 0.000 -

Sequence-2360-S 0.071 0.000 0.000 -

Sequence-2361-P 0.147 0.000 0.000 -

Sequence-2362-P 0.072 0.000 0.000 -

Sequence-2363-F 0.082 0.000 0.000 -

Sequence-2364-H 0.081 0.000 0.000 -

Sequence-2365-Y 0.075 0.000 0.000 -

Sequence-2366-N 0.076 0.000 0.000 -

Sequence-2367-T 0.082 0.000 0.000 -

Sequence-2368-H 0.091 0.000 0.000 -

Sequence-2369-Y 0.095 0.000 0.000 -

Sequence-2370-S 0.083 0.000 0.000 -

Sequence-2371-T 0.081 0.000 0.000 -

Sequence-2372-A 0.088 0.000 0.000 -

Sequence-2373-T 0.066 0.000 0.000 -

Sequence-2374-S 0.078 0.000 0.000 -

Sequence-2375-A 0.077 0.000 0.000 -

Sequence-2376-L 0.111 0.002 0.000 -

Sequence-2377-S 0.067 0.002 0.000 -

Sequence-2378-W 0.130 0.002 0.000 -

Sequence-2379-L 0.179 0.002 0.000 -

Sequence-2380-V 0.079 0.002 0.000 -

Sequence-2381-R 0.265 0.002 0.000 -

Sequence-2382-I 0.084 0.002 0.000 -

Sequence-2383-E 0.101 0.000 0.000 -

Sequence-2384-P 0.128 0.000 0.000 -

Sequence-2385-F 0.090 0.000 0.000 -

Sequence-2386-T 0.089 0.000 0.000 -

Sequence-2387-T 0.121 0.000 0.000 -

Sequence-2388-F 0.088 0.000 0.000 -

Sequence-2389-F 0.088 0.000 0.000 -

Sequence-2390-L 0.168 0.000 0.000 -

Sequence-2391-N 0.079 0.000 0.000 -

Sequence-2392-A 0.081 0.000 0.000 -

Sequence-2393-N 0.073 0.000 0.000 -

Sequence-2394-D 0.118 0.000 0.000 -

Sequence-2395-G 0.081 0.000 0.000 -

Sequence-2396-K 0.070 0.000 0.000 -

Sequence-2397-F 0.084 0.000 0.000 -

Sequence-2398-D 0.083 0.000 0.000 -

Sequence-2399-H 0.130 0.000 0.000 -

Sequence-2400-P 0.084 0.000 0.000 -

Sequence-2401-D 0.073 0.000 0.000 -

Sequence-2402-R 0.083 0.000 0.000 -

Sequence-2403-T 0.079 0.000 0.000 -

Sequence-2404-F 0.084 0.000 0.000 -

Sequence-2405-S 0.078 0.000 0.000 -

Sequence-2406-S 0.123 0.000 0.000 -

Sequence-2407-I 0.087 0.000 0.000 -

Sequence-2408-A 0.076 0.000 0.000 -

Sequence-2409-R 0.101 0.000 0.000 -

Sequence-2410-S 0.074 0.000 0.000 -

Sequence-2411-W 0.076 0.000 0.000 -

Sequence-2412-R 0.086 0.000 0.000 -

Sequence-2413-T 0.072 0.000 0.000 -

Sequence-2414-S 0.090 0.000 0.000 -

Sequence-2415-Q 0.082 0.000 0.000 -

Sequence-2416-R 0.119 0.000 0.000 -

Sequence-2417-D 0.068 0.000 0.000 -

Sequence-2418-T 0.081 0.000 0.000 -

Sequence-2419-S 0.074 0.000 0.000 -

Sequence-2420-D 0.076 0.000 0.000 -

Sequence-2421-V 0.083 0.000 0.000 -

Sequence-2422-K 0.067 0.000 0.000 -

Sequence-2423-E 0.085 0.000 0.000 -

Sequence-2424-L 0.082 0.000 0.000 -

Sequence-2425-I 0.470 0.000 0.173 -

Sequence-2426-P 0.104 0.000 0.000 -

Sequence-2427-E 0.074 0.000 0.000 -

Sequence-2428-F 0.094 0.000 0.000 -

Sequence-2429-Y 0.124 0.000 0.000 -

Sequence-2430-Y 0.101 0.000 0.000 -

Sequence-2431-L 0.134 0.000 0.000 -

Sequence-2432-P 0.075 0.000 0.000 -

Sequence-2433-E 0.191 0.000 0.000 -

Sequence-2434-M 0.155 0.000 0.000 -

Sequence-2435-F 0.077 0.000 0.000 -

Sequence-2436-V 0.133 0.000 0.000 -

Sequence-2437-N 0.070 0.000 0.000 -

Sequence-2438-S 0.069 0.000 0.000 -

Sequence-2439-N 0.078 0.000 0.000 -

Sequence-2440-G 0.100 0.000 0.000 -

Sequence-2441-Y 0.074 0.000 0.000 -

Sequence-2442-H 0.097 0.000 0.000 -

Sequence-2443-L 0.094 0.000 0.000 -

Sequence-2444-G 0.081 0.000 0.000 -

Sequence-2445-V 0.091 0.001 0.000 -

Sequence-2446-R 0.075 0.001 0.000 -

Sequence-2447-E 0.090 0.001 0.000 -

Sequence-2448-D 0.089 0.001 0.000 -

Sequence-2449-E 0.076 0.001 0.000 -

Sequence-2450-V 0.084 0.001 0.000 -

Sequence-2451-V 0.076 0.001 0.000 -

Sequence-2452-V 0.097 0.001 0.000 -

Sequence-2453-N 0.095 0.001 0.000 -

Sequence-2454-D 0.071 0.001 0.000 -

Sequence-2455-V 0.078 0.001 0.000 -

Sequence-2456-D 0.075 0.001 0.000 -

Sequence-2457-L 0.219 0.002 0.000 -

Sequence-2458-P 0.073 0.001 0.000 -

Sequence-2459-P 0.113 0.001 0.000 -

Sequence-2460-W 0.077 0.001 0.000 -

Sequence-2461-A 0.074 0.001 0.000 -

Sequence-2462-K 0.108 0.002 0.000 -

Sequence-2463-K 0.081 0.002 0.000 -

Sequence-2464-P 0.075 0.002 0.000 -

Sequence-2465-E 0.068 0.002 0.000 -

Sequence-2466-D 0.079 0.002 0.000 -

Sequence-2467-F 0.086 0.004 0.000 -

Sequence-2468-V 0.079 0.016 0.000 -

Sequence-2469-R 0.084 0.016 0.000 -

Sequence-2470-I 0.103 0.016 0.000 -

Sequence-2471-N 0.074 0.015 0.000 -

Sequence-2472-R 0.088 0.015 0.000 -

Sequence-2473-M 0.144 0.024 0.000 -

Sequence-2474-A 0.092 0.024 0.000 -

Sequence-2475-L 0.166 0.024 0.000 -

Sequence-2476-E 0.071 0.009 0.000 -

Sequence-2477-S 0.104 0.009 0.000 -

Sequence-2478-E 0.080 0.009 0.000 -

Sequence-2479-F 0.098 0.009 0.000 -

Sequence-2480-V 0.161 0.008 0.000 -

Sequence-2481-S 0.079 0.004 0.000 -

Sequence-2482-C 0.087 0.004 0.000 -

Sequence-2483-Q 0.068 0.004 0.000 -

Sequence-2484-L 0.113 0.005 0.000 -

Sequence-2485-H 0.090 0.000 0.000 -

Sequence-2486-Q 0.089 0.000 0.000 -

Sequence-2487-W 0.100 0.000 0.000 -

Sequence-2488-I 0.108 0.000 0.000 -

Sequence-2489-D 0.073 0.000 0.000 -

Sequence-2490-L 0.382 0.000 0.000 -

Sequence-2491-I 0.124 0.000 0.000 -

Sequence-2492-F 0.138 0.000 0.000 -

Sequence-2493-G 0.138 0.000 0.000 -

Sequence-2494-Y 0.087 0.000 0.000 -

Sequence-2495-K 0.079 0.000 0.000 -

Sequence-2496-Q 0.080 0.000 0.000 -

Sequence-2497-R 0.146 0.000 0.000 -

Sequence-2498-G 0.074 0.000 0.000 -

Sequence-2499-P 0.081 0.000 0.000 -

Sequence-2500-E 0.075 0.000 0.000 -

Sequence-2501-A 0.068 0.000 0.000 -

Sequence-2502-V 0.073 0.000 0.000 -

Sequence-2503-R 0.067 0.000 0.000 -

Sequence-2504-A 0.078 0.000 0.000 -

Sequence-2505-L 0.073 0.000 0.000 -

Sequence-2506-N 0.078 0.000 0.000 -

Sequence-2507-V 0.199 0.000 0.000 -

Sequence-2508-F 0.074 0.000 0.000 -

Sequence-2509-H 0.087 0.000 0.000 -

Sequence-2510-Y 0.104 0.000 0.000 -

Sequence-2511-L 0.106 0.000 0.000 -

Sequence-2512-T 0.073 0.000 0.000 -

Sequence-2513-Y 0.248 0.000 0.000 -

Sequence-2514-E 0.078 0.000 0.000 -

Sequence-2515-G 0.098 0.000 0.000 -

Sequence-2516-S 0.076 0.000 0.000 -

Sequence-2517-V 0.074 0.000 0.000 -

Sequence-2518-N 0.073 0.000 0.000 -

Sequence-2519-L 0.141 0.002 0.000 -

Sequence-2520-D 0.082 0.002 0.000 -

Sequence-2521-S 0.076 0.002 0.000 -

Sequence-2522-I 0.096 0.006 0.000 -

Sequence-2523-T 0.071 0.007 0.000 -

Sequence-2524-D 0.116 0.007 0.000 -

Sequence-2525-P 0.094 0.007 0.000 -

Sequence-2526-V 0.083 0.007 0.000 -

Sequence-2527-L 0.144 0.007 0.000 -

Sequence-2528-R 0.132 0.007 0.000 -

Sequence-2529-E 0.108 0.007 0.000 -

Sequence-2530-A 0.088 0.007 0.000 -

Sequence-2531-M 0.190 0.007 0.000 -

Sequence-2532-E 0.085 0.005 0.000 -

Sequence-2533-A 0.085 0.005 0.000 -

Sequence-2534-Q 0.083 0.005 0.000 -

Sequence-2535-I 0.130 0.005 0.000 -

Sequence-2536-Q 0.081 0.000 0.000 -

Sequence-2537-N 0.085 0.000 0.000 -

Sequence-2538-F 0.081 0.000 0.000 -

Sequence-2539-G 0.078 0.000 0.000 -

Sequence-2540-Q 0.072 0.000 0.000 -

Sequence-2541-T 0.072 0.000 0.000 -

Sequence-2542-P 0.071 0.000 0.000 -

Sequence-2543-S 0.074 0.000 0.000 -

Sequence-2544-Q 0.076 0.000 0.000 -

Sequence-2545-L 0.097 0.000 0.000 -

Sequence-2546-L 0.074 0.001 0.000 -

Sequence-2547-I 0.137 0.002 0.000 -

Sequence-2548-E 0.078 0.001 0.000 -

Sequence-2549-P 0.069 0.001 0.000 -

Sequence-2550-H 0.195 0.001 0.000 -

Sequence-2551-P 0.109 0.001 0.000 -

Sequence-2552-P 0.106 0.001 0.000 -

Sequence-2553-R 0.084 0.001 0.000 -

Sequence-2554-S 0.078 0.001 0.000 -

Sequence-2555-S 0.098 0.001 0.000 -

Sequence-2556-A 0.098 0.001 0.000 -

Sequence-2557-M 0.070 0.002 0.000 -

Sequence-2558-H 0.079 0.002 0.000 -

Sequence-2559-L 0.103 0.002 0.000 -

Sequence-2560-C 0.070 0.000 0.000 -

Sequence-2561-F 0.101 0.000 0.000 -

Sequence-2562-L 0.100 0.000 0.000 -

Sequence-2563-P 0.084 0.000 0.000 -

Sequence-2564-Q 0.220 0.000 0.000 -

Sequence-2565-S 0.072 0.000 0.000 -

Sequence-2566-P 0.100 0.000 0.000 -

Sequence-2567-L 0.138 0.000 0.000 -

Sequence-2568-M 0.090 0.000 0.000 -

Sequence-2569-F 0.134 0.000 0.000 -

Sequence-2570-K 0.080 0.000 0.000 -

Sequence-2571-D 0.073 0.000 0.000 -

Sequence-2572-Q 0.098 0.000 0.000 -

Sequence-2573-M 0.082 0.005 0.000 -

Sequence-2574-Q 0.104 0.005 0.000 -

Sequence-2575-Q 0.106 0.005 0.000 -

Sequence-2576-D 0.073 0.005 0.000 -

Sequence-2577-V 0.098 0.005 0.000 -

Sequence-2578-I 0.094 0.004 0.000 -

Sequence-2579-M 0.082 0.004 0.000 -

Sequence-2580-V 0.080 0.004 0.000 -

Sequence-2581-L 0.194 0.004 0.000 -

Sequence-2582-K 0.085 0.000 0.000 -

Sequence-2583-F 0.140 0.000 0.000 -

Sequence-2584-P 0.076 0.000 0.000 -

Sequence-2585-S 0.088 0.000 0.000 -

Sequence-2586-N 0.096 0.000 0.000 -

Sequence-2587-S 0.069 0.000 0.000 -

Sequence-2588-P 0.108 0.000 0.000 -

Sequence-2589-V 0.093 0.000 0.000 -

Sequence-2590-T 0.067 0.000 0.000 -

Sequence-2591-H 0.087 0.000 0.000 -

Sequence-2592-V 0.070 0.001 0.000 -

Sequence-2593-A 0.076 0.001 0.000 -

Sequence-2594-A 0.083 0.001 0.000 -

Sequence-2595-N 0.069 0.001 0.000 -

Sequence-2596-T 0.071 0.004 0.000 -

Sequence-2597-L 0.321 0.004 0.000 -

Sequence-2598-P 0.070 0.004 0.000 -

Sequence-2599-H 0.099 0.004 0.000 -

Sequence-2600-L 0.088 0.005 0.000 -

Sequence-2601-T 0.079 0.005 0.000 -

Sequence-2602-I 0.406 0.005 0.218 -

Sequence-2603-P 0.074 0.001 0.000 -

Sequence-2604-A 0.088 0.001 0.000 -

Sequence-2605-V 0.115 0.001 0.000 -

Sequence-2606-V 0.077 0.001 0.000 -

Sequence-2607-T 0.085 0.001 0.000 -

Sequence-2608-V 0.101 0.001 0.000 -

Sequence-2609-T 0.075 0.000 0.000 -

Sequence-2610-C 0.081 0.000 0.000 -

Sequence-2611-S 0.069 0.000 0.000 -

Sequence-2612-R 0.091 0.000 0.000 -

Sequence-2613-L 0.111 0.000 0.000 -

Sequence-2614-F 0.085 0.000 0.000 -

Sequence-2615-A 0.090 0.000 0.000 -

Sequence-2616-V 0.072 0.000 0.000 -

Sequence-2617-N 0.077 0.000 0.000 -

Sequence-2618-R 0.101 0.000 0.000 -

Sequence-2619-W 0.094 0.000 0.000 -

Sequence-2620-H 0.081 0.000 0.000 -

Sequence-2621-N 0.086 0.000 0.000 -

Sequence-2622-T 0.093 0.000 0.000 -

Sequence-2623-V 0.082 0.000 0.000 -

Sequence-2624-G 0.066 0.000 0.000 -

Sequence-2625-L 0.087 0.000 0.000 -

Sequence-2626-R 0.068 0.000 0.000 -

Sequence-2627-G 0.079 0.000 0.000 -

Sequence-2628-A 0.079 0.000 0.000 -

Sequence-2629-P 0.074 0.000 0.000 -

Sequence-2630-G 0.081 0.000 0.000 -

Sequence-2631-Y 0.078 0.000 0.000 -

Sequence-2632-S 0.074 0.000 0.000 -

Sequence-2633-L 0.108 0.010 0.000 -

Sequence-2634-D 0.085 0.010 0.000 -

Sequence-2635-Q 0.099 0.010 0.000 -

Sequence-2636-A 0.069 0.010 0.000 -

Sequence-2637-H 0.067 0.010 0.000 -

Sequence-2638-H 0.092 0.010 0.000 -

Sequence-2639-L 0.095 0.103 0.000 -

Sequence-2640-P 0.069 0.103 0.000 -

Sequence-2641-I 0.291 0.103 0.000 -

Sequence-2642-E 0.076 0.095 0.000 -

Sequence-2643-M 0.144 0.096 0.000 -

Sequence-2644-D 0.103 0.095 0.000 -

Sequence-2645-P 0.094 0.095 0.000 -

Sequence-2646-L 0.138 0.095 0.000 -

Sequence-2647-I 0.083 0.003 0.000 -

Sequence-2648-A 0.116 0.000 0.000 -

Sequence-2649-N 0.173 0.000 0.000 -

Sequence-2650-N 0.097 0.000 0.000 -

Sequence-2651-S 0.087 0.000 0.000 -

Sequence-2652-G 0.082 0.000 0.000 -

Sequence-2653-V 0.069 0.000 0.000 -

Sequence-2654-N 0.078 0.000 0.000 -

Sequence-2655-K 0.078 0.002 0.000 -

Sequence-2656-R 0.074 0.002 0.000 -

Sequence-2657-Q 0.074 0.002 0.000 -

Sequence-2658-I 0.097 0.052 0.000 -

Sequence-2659-T 0.068 0.063 0.000 -

Sequence-2660-D 0.069 0.063 0.000 -

Sequence-2661-L 0.087 0.065 0.000 -

Sequence-2662-V 0.071 0.065 0.000 -

Sequence-2663-D 0.100 0.065 0.000 -

Sequence-2664-Q 0.099 0.065 0.000 -

Sequence-2665-S 0.085 0.065 0.000 -

Sequence-2666-I 0.207 0.065 0.000 -

Sequence-2667-Q 0.090 0.065 0.000 -

Sequence-2668-I 0.119 0.065 0.000 -

Sequence-2669-N 0.074 0.000 0.000 -

Sequence-2670-A 0.075 0.000 0.000 -

Sequence-2671-H 0.135 0.000 0.000 -

Sequence-2672-C 0.067 0.000 0.000 -

Sequence-2673-F 0.076 0.000 0.000 -

Sequence-2674-V 0.107 0.000 0.000 -

Sequence-2675-V 0.085 0.000 0.000 -

Sequence-2676-T 0.088 0.000 0.000 -

Sequence-2677-A 0.085 0.000 0.000 -

Sequence-2678-D 0.078 0.000 0.000 -

Sequence-2679-N 0.072 0.000 0.000 -

Sequence-2680-R 0.069 0.000 0.000 -

Sequence-2681-Y 0.071 0.000 0.000 -

Sequence-2682-I 0.098 0.000 0.000 -

Sequence-2683-L 0.123 0.000 0.000 -

Sequence-2684-I 0.209 0.000 0.000 -

Sequence-2685-C 0.100 0.000 0.000 -

Sequence-2686-G 0.081 0.000 0.000 -

Sequence-2687-F 0.100 0.000 0.000 -

Sequence-2688-W 0.081 0.000 0.000 -

Sequence-2689-D 0.104 0.000 0.000 -

Sequence-2690-K 0.077 0.000 0.000 -

Sequence-2691-S 0.074 0.000 0.000 -

Sequence-2692-F 0.102 0.000 0.000 -

Sequence-2693-R 0.103 0.000 0.000 -

Sequence-2694-V 0.081 0.000 0.000 -

Sequence-2695-Y 0.088 0.000 0.000 -

Sequence-2696-S 0.069 0.000 0.000 -

Sequence-2697-T 0.081 0.000 0.000 -

Sequence-2698-E 0.073 0.000 0.000 -

Sequence-2699-T 0.084 0.000 0.000 -

Sequence-2700-G 0.085 0.000 0.000 -

Sequence-2701-K 0.084 0.000 0.000 -

Sequence-2702-L 0.123 0.000 0.000 -

Sequence-2703-T 0.067 0.000 0.000 -

Sequence-2704-Q 0.259 0.000 0.000 -

Sequence-2705-I 0.115 0.000 0.000 -

Sequence-2706-V 0.082 0.000 0.000 -

Sequence-2707-F 0.100 0.000 0.000 -

Sequence-2708-G 0.096 0.000 0.000 -

Sequence-2709-H 0.085 0.000 0.000 -

Sequence-2710-W 0.087 0.000 0.000 -

Sequence-2711-D 0.085 0.000 0.000 -

Sequence-2712-V 0.125 0.000 0.000 -

Sequence-2713-V 0.075 0.000 0.000 -

Sequence-2714-T 0.083 0.000 0.000 -

Sequence-2715-C 0.090 0.000 0.000 -

Sequence-2716-L 0.081 0.000 0.000 -

Sequence-2717-A 0.077 0.000 0.000 -

Sequence-2718-R 0.101 0.000 0.000 -

Sequence-2719-S 0.084 0.000 0.000 -

Sequence-2720-E 0.078 0.000 0.000 -

Sequence-2721-S 0.145 0.000 0.000 -

Sequence-2722-Y 0.093 0.000 0.000 -

Sequence-2723-I 0.145 0.000 0.000 -

Sequence-2724-G 0.084 0.000 0.000 -

Sequence-2725-G 0.091 0.000 0.000 -

Sequence-2726-D 0.076 0.000 0.000 -

Sequence-2727-C 0.076 0.000 0.000 -

Sequence-2728-Y 0.069 0.000 0.000 -

Sequence-2729-I 0.103 0.000 0.000 -

Sequence-2730-V 0.082 0.000 0.000 -

Sequence-2731-S 0.100 0.000 0.000 -

Sequence-2732-G 0.084 0.000 0.000 -

Sequence-2733-S 0.074 0.000 0.000 -

Sequence-2734-R 0.071 0.000 0.000 -

Sequence-2735-D 0.073 0.000 0.000 -

Sequence-2736-A 0.073 0.000 0.000 -

Sequence-2737-T 0.070 0.000 0.000 -

Sequence-2738-L 0.158 0.000 0.000 -

Sequence-2739-L 0.091 0.000 0.000 -

Sequence-2740-L 0.495 0.000 0.092 -

Sequence-2741-W 0.107 0.000 0.000 -

Sequence-2742-Y 0.257 0.000 0.000 -

Sequence-2743-W 0.105 0.000 0.000 -

Sequence-2744-S 0.101 0.000 0.000 -

Sequence-2745-G 0.154 0.000 0.000 -

Sequence-2746-R 0.078 0.000 0.000 -

Sequence-2747-H 0.148 0.000 0.000 -

Sequence-2748-H 0.122 0.000 0.000 -

Sequence-2749-I 0.249 0.000 0.000 -

Sequence-2750-I 0.107 0.000 0.000 -

Sequence-2751-G 0.068 0.000 0.000 -

Sequence-2752-D 0.066 0.000 0.000 -

Sequence-2753-N 0.073 0.000 0.000 -

Sequence-2754-P 0.069 0.000 0.000 -

Sequence-2755-N 0.086 0.000 0.000 -

Sequence-2756-S 0.067 0.000 0.000 -

Sequence-2757-S 0.066 0.000 0.000 -

Sequence-2758-D 0.075 0.000 0.000 -

Sequence-2759-Y 0.078 0.000 0.000 -

Sequence-2760-P 0.069 0.000 0.000 -

Sequence-2761-A 0.067 0.000 0.000 -

Sequence-2762-P 0.064 0.000 0.000 -

Sequence-2763-R 0.070 0.000 0.000 -

Sequence-2764-A 0.076 0.000 0.000 -

Sequence-2765-V 0.076 0.000 0.000 -

Sequence-2766-L 0.311 0.000 0.000 -

Sequence-2767-T 0.071 0.000 0.000 -

Sequence-2768-G 0.100 0.000 0.000 -

Sequence-2769-H 0.070 0.000 0.000 -

Sequence-2770-D 0.079 0.000 0.000 -

Sequence-2771-H 0.101 0.000 0.000 -

Sequence-2772-E 0.073 0.000 0.000 -

Sequence-2773-V 0.085 0.000 0.000 -

Sequence-2774-V 0.082 0.000 0.000 -

Sequence-2775-C 0.097 0.000 0.000 -

Sequence-2776-V 0.105 0.005 0.000 -

Sequence-2777-S 0.072 0.005 0.000 -

Sequence-2778-V 0.089 0.010 0.000 -

Sequence-2779-C 0.083 0.010 0.000 -

Sequence-2780-A 0.072 0.010 0.000 -

Sequence-2781-E 0.084 0.010 0.000 -

Sequence-2782-L 0.091 0.010 0.000 -

Sequence-2783-G 0.089 0.010 0.000 -

Sequence-2784-L 0.291 0.010 0.000 -

Sequence-2785-V 0.080 0.007 0.000 -

Sequence-2786-I 0.267 0.007 0.000 -

Sequence-2787-S 0.088 0.000 0.000 -

Sequence-2788-G 0.097 0.000 0.000 -

Sequence-2789-A 0.097 0.000 0.000 -

Sequence-2790-K 0.081 0.000 0.000 -

Sequence-2791-E 0.097 0.000 0.000 -

Sequence-2792-G 0.097 0.000 0.000 -

Sequence-2793-P 0.074 0.000 0.000 -

Sequence-2794-C 0.077 0.000 0.000 -

Sequence-2795-L 0.083 0.000 0.000 -

Sequence-2796-V 0.078 0.000 0.000 -

Sequence-2797-H 0.089 0.000 0.000 -

Sequence-2798-T 0.077 0.000 0.000 -

Sequence-2799-I 0.088 0.000 0.000 -

Sequence-2800-T 0.080 0.000 0.000 -

Sequence-2801-G 0.109 0.000 0.000 -

Sequence-2802-D 0.078 0.000 0.000 -

Sequence-2803-L 0.194 0.000 0.000 -

Sequence-2804-L 0.109 0.000 0.000 -

Sequence-2805-R 0.203 0.000 0.000 -

Sequence-2806-A 0.085 0.000 0.000 -

Sequence-2807-L 0.217 0.000 0.000 -

Sequence-2808-E 0.117 0.000 0.000 -

Sequence-2809-G 0.143 0.000 0.000 -

Sequence-2810-P 0.107 0.000 0.000 -

Sequence-2811-E 0.086 0.000 0.000 -

Sequence-2812-N 0.121 0.000 0.000 -

Sequence-2813-C 0.086 0.000 0.000 -

Sequence-2814-L 0.115 0.000 0.000 -

Sequence-2815-F 0.069 0.000 0.000 -

Sequence-2816-P 0.095 0.000 0.000 -

Sequence-2817-R 0.076 0.000 0.000 -

Sequence-2818-L 0.109 0.000 0.000 -

Sequence-2819-I 0.120 0.000 0.000 -

Sequence-2820-S 0.097 0.000 0.000 -

Sequence-2821-V 0.087 0.000 0.000 -

Sequence-2822-S 0.091 0.000 0.000 -

Sequence-2823-S 0.103 0.000 0.000 -

Sequence-2824-E 0.087 0.000 0.000 -

Sequence-2825-G 0.070 0.000 0.000 -

Sequence-2826-H 0.078 0.000 0.000 -

Sequence-2827-C 0.084 0.000 0.000 -

Sequence-2828-I 0.084 0.000 0.000 -

Sequence-2829-I 0.084 0.000 0.000 -

Sequence-2830-Y 0.084 0.000 0.000 -

Sequence-2831-Y 0.076 0.000 0.000 -

Sequence-2832-E 0.070 0.000 0.000 -

Sequence-2833-R 0.092 0.000 0.000 -

Sequence-2834-G 0.092 0.000 0.000 -

Sequence-2835-R 0.072 0.000 0.000 -

Sequence-2836-F 0.090 0.000 0.000 -

Sequence-2837-S 0.083 0.000 0.000 -

Sequence-2838-N 0.093 0.000 0.000 -

Sequence-2839-F 0.081 0.000 0.000 -

Sequence-2840-S 0.065 0.000 0.000 -

Sequence-2841-I 0.110 0.000 0.000 -

Sequence-2842-N 0.069 0.000 0.000 -

Sequence-2843-G 0.072 0.000 0.000 -

Sequence-2844-K 0.074 0.001 0.000 -

Sequence-2845-L 0.080 0.001 0.000 -

Sequence-2846-L 0.077 0.001 0.000 -

Sequence-2847-A 0.108 0.001 0.000 -

Sequence-2848-Q 0.122 0.001 0.000 -

Sequence-2849-M 0.089 0.002 0.000 -

Sequence-2850-E 0.089 0.002 0.000 -

Sequence-2851-I 0.548 0.002 0.219 -

Sequence-2852-N 0.087 0.000 0.000 -

Sequence-2853-D 0.081 0.000 0.000 -

Sequence-2854-S 0.089 0.000 0.000 -

Sequence-2855-T 0.080 0.000 0.000 -

Sequence-2856-R 0.098 0.000 0.000 -

Sequence-2857-A 0.074 0.000 0.000 -

Sequence-2858-I 0.120 0.000 0.000 -

Sequence-2859-L 0.088 0.000 0.000 -

Sequence-2860-L 0.111 0.000 0.000 -

Sequence-2861-S 0.115 0.000 0.000 -

Sequence-2862-S 0.107 0.000 0.000 -

Sequence-2863-D 0.144 0.000 0.000 -

Sequence-2864-G 0.106 0.000 0.000 -

Sequence-2865-Q 0.088 0.000 0.000 -

Sequence-2866-N 0.088 0.000 0.000 -

Sequence-2867-L 0.135 0.000 0.000 -

Sequence-2868-V 0.107 0.000 0.000 -

Sequence-2869-T 0.159 0.000 0.000 -

Sequence-2870-G 0.079 0.000 0.000 -

Sequence-2871-G 0.076 0.000 0.000 -

Sequence-2872-D 0.068 0.000 0.000 -

Sequence-2873-N 0.076 0.000 0.000 -

Sequence-2874-G 0.071 0.000 0.000 -

Sequence-2875-V 0.084 0.000 0.000 -

Sequence-2876-V 0.099 0.000 0.000 -

Sequence-2877-E 0.088 0.000 0.000 -

Sequence-2878-V 0.072 0.000 0.000 -

Sequence-2879-W 0.083 0.000 0.000 -

Sequence-2880-Q 0.081 0.000 0.000 -

Sequence-2881-A 0.076 0.000 0.000 -

Sequence-2882-C 0.071 0.000 0.000 -

Sequence-2883-D 0.069 0.000 0.000 -

Sequence-2884-F 0.090 0.000 0.000 -

Sequence-2885-K 0.070 0.000 0.000 -

Sequence-2886-Q 0.082 0.000 0.000 -

Sequence-2887-L 0.145 0.000 0.000 -

Sequence-2888-Y 0.074 0.000 0.000 -

Sequence-2889-I 0.205 0.000 0.000 -

Sequence-2890-Y 0.084 0.000 0.000 -

Sequence-2891-P 0.110 0.000 0.000 -

Sequence-2892-G 0.076 0.000 0.000 -

Sequence-2893-C 0.079 0.000 0.000 -

Sequence-2894-D 0.078 0.000 0.000 -

Sequence-2895-A 0.095 0.000 0.000 -

Sequence-2896-G 0.104 0.000 0.000 -

Sequence-2897-I 0.186 0.000 0.000 -

Sequence-2898-R 0.078 0.000 0.000 -

Sequence-2899-A 0.077 0.000 0.000 -

Sequence-2900-M 0.094 0.000 0.000 -

Sequence-2901-D 0.077 0.000 0.000 -

Sequence-2902-L 0.246 0.000 0.000 -

Sequence-2903-S 0.067 0.000 0.000 -

Sequence-2904-H 0.100 0.000 0.000 -

Sequence-2905-D 0.075 0.000 0.000 -

Sequence-2906-Q 0.081 0.000 0.000 -

Sequence-2907-R 0.106 0.000 0.000 -

Sequence-2908-T 0.084 0.000 0.000 -

Sequence-2909-L 0.334 0.000 0.000 -

Sequence-2910-I 0.147 0.000 0.000 -

Sequence-2911-T 0.099 0.000 0.000 -

Sequence-2912-G 0.080 0.000 0.000 -

Sequence-2913-M 0.121 0.000 0.000 -

Sequence-2914-A 0.083 0.000 0.000 -

Sequence-2915-S 0.096 0.000 0.000 -

Sequence-2916-G 0.071 0.000 0.000 -

Sequence-2917-S 0.075 0.000 0.000 -

Sequence-2918-I 0.120 0.000 0.000 -

Sequence-2919-V 0.089 0.000 0.000 -

Sequence-2920-A 0.074 0.000 0.000 -

Sequence-2921-F 0.084 0.000 0.000 -

Sequence-2922-N 0.070 0.000 0.000 -

Sequence-2923-I 0.087 0.000 0.000 -

Sequence-2924-D 0.066 0.000 0.000 -

Sequence-2925-F 0.072 0.000 0.000 -

Sequence-2926-N 0.078 0.000 0.000 -

Sequence-2927-R 0.083 0.000 0.000 -

Sequence-2928-W 0.081 0.000 0.000 -

Sequence-2929-H 0.070 0.000 0.000 -

Sequence-2930-Y 0.088 0.000 0.000 -

Sequence-2931-E 0.073 0.000 0.000 -

Sequence-2932-H 0.078 0.000 0.000 -

Sequence-2933-Q 0.070 0.000 0.000 -

Sequence-2934-N 0.070 0.000 0.000 -

Sequence-2935-R 0.071 0.000 0.000 -

Sequence-2936-Y 0.072 0.000 0.000 -

//
