# Supplementary figures and images for: Nuclear Localization of the Autism Candidate Gene Neurobeachin and Functional Interaction with the NOTCH1 Intracellular Domain Indicate a Role in Regulating Transcription
Source: PLoS One. 2016 Mar 21;11(3):e0151954. doi: 10.1371/journal.pone.0151954 (PMC4801420; doi:10.1371/journal.pone.0151954)

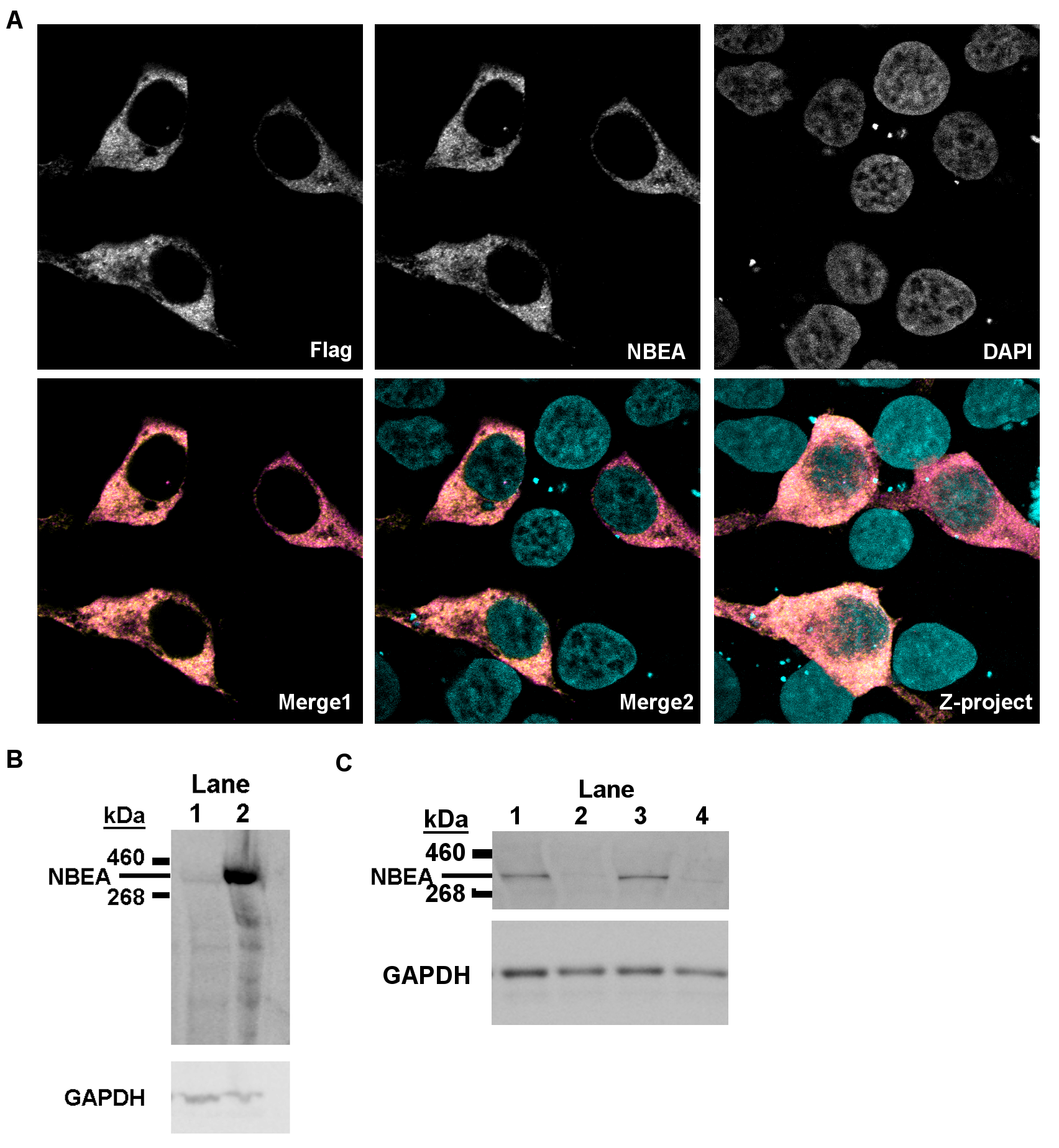

Supplement: S1 Fig — A) HEK293T cells, which do not express NBEA endogenously, were transfected with the pcDNA3.1-FLAG-Nbea construct. Confocal images show an exclusive colocalization between Flag (yellow) and NBEA (magenta) for cells that overexpress FLAG-NBEA. Untransfected HEK293T cells do not show any non-specific immunostaining for NBEA. Nuclei were stained with DAPI (cyan). Separate confocal images are shown in greyscale. A 2D Z-projection of the confocal Z-stack is shown as well (Z-project). B) The NBEA antibody detects overexpressed FLAG-NBEA at the expected molecular weight of 327 kDa in HEK293T cell lysate (lane 2) compared to untransfected HEK293T cells (lane 1). GAPDH is shown for normalization. C) The protein of 327 kDa is endogenously detected by the home-made antibody in N2a cell lysates (lane 1 and 3). This is NBEA-specific, since this fragment cannot be detected anymore in N2a cell lysates transfected with Nbea-specific shRNA (lane 1 and 3). GAPDH is shown for normalization. (TIF) [file pone.0151954.s003.tif]

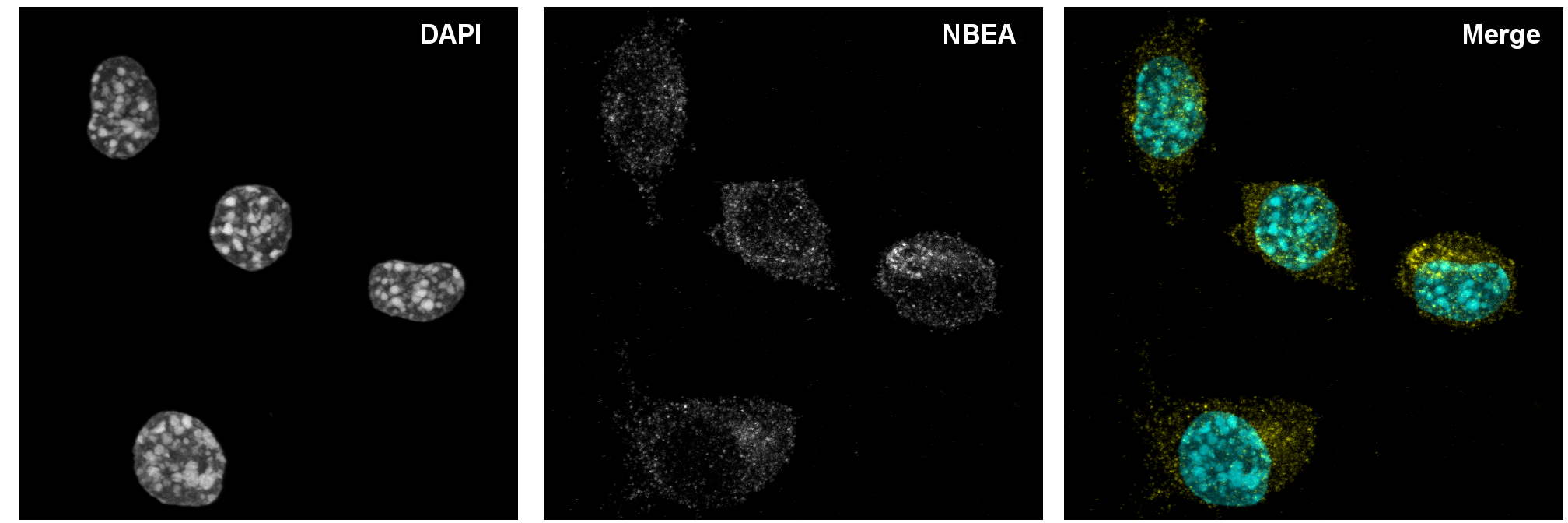

Supplement: S2 Fig — A 2D Z-projection of endogenous NBEA (yellow) in N2a cells is shown. DAPI (cyan) was used to stain the nucleus. The separate images are shown in greyscale. (TIF) [file pone.0151954.s004.tif]

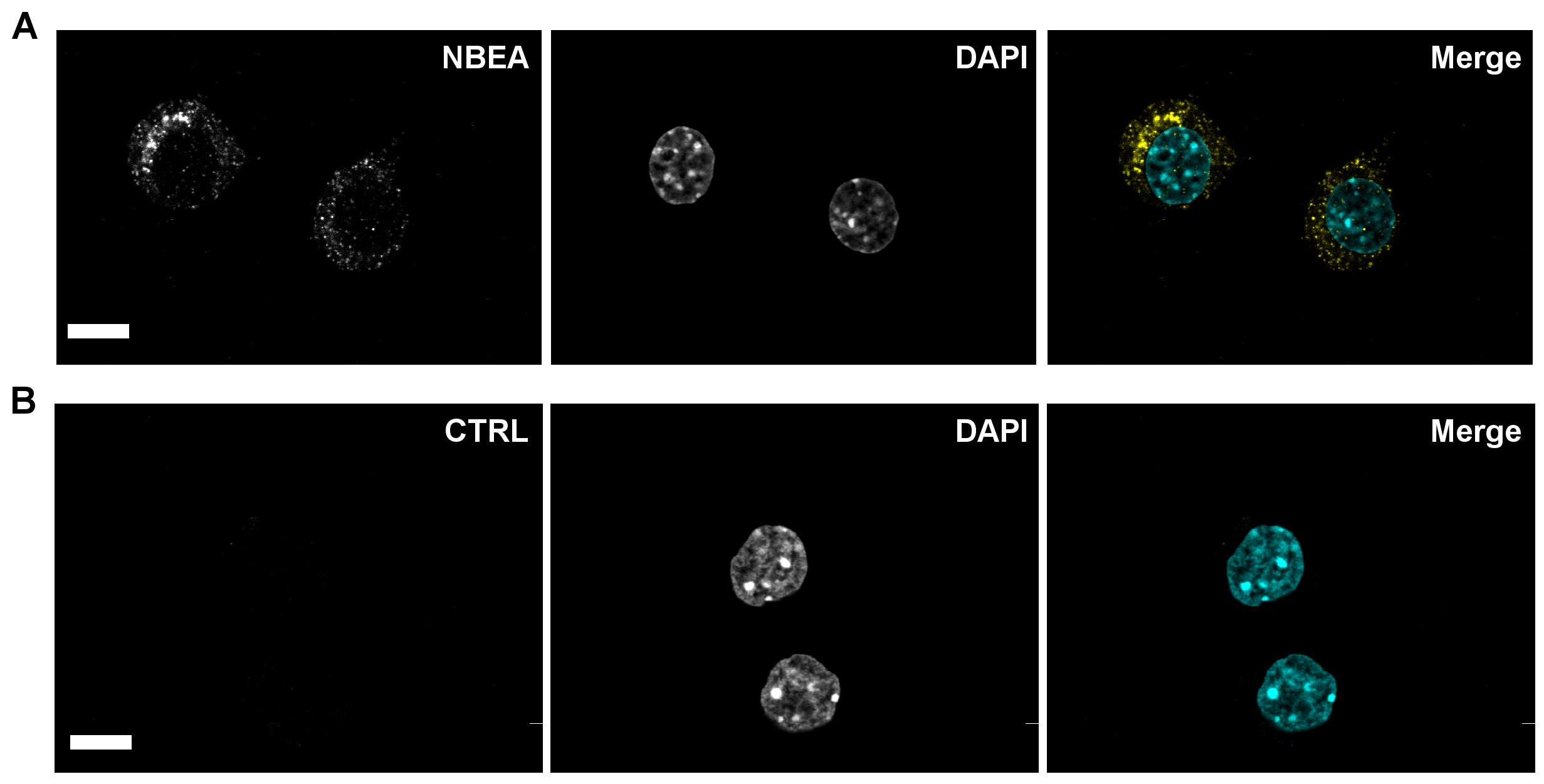

Supplement: S3 Fig — A) Confocal image of endogenous NBEA (yellow) in N2a cells. DAPI (cyan) was used to stain the nucleus. The separate images are shown in greyscale. Scale (white bar) is 10 μm. B) Confocal image as a control for background staining, where no primary antibody, but the same secondary antibody (yellow) and confocal settings were used. DAPI (Cyan) was used to stain the nucleus. The separate images are shown in greyscale. Scale (white bar) is 10 μm. (TIF) [file pone.0151954.s005.tif]

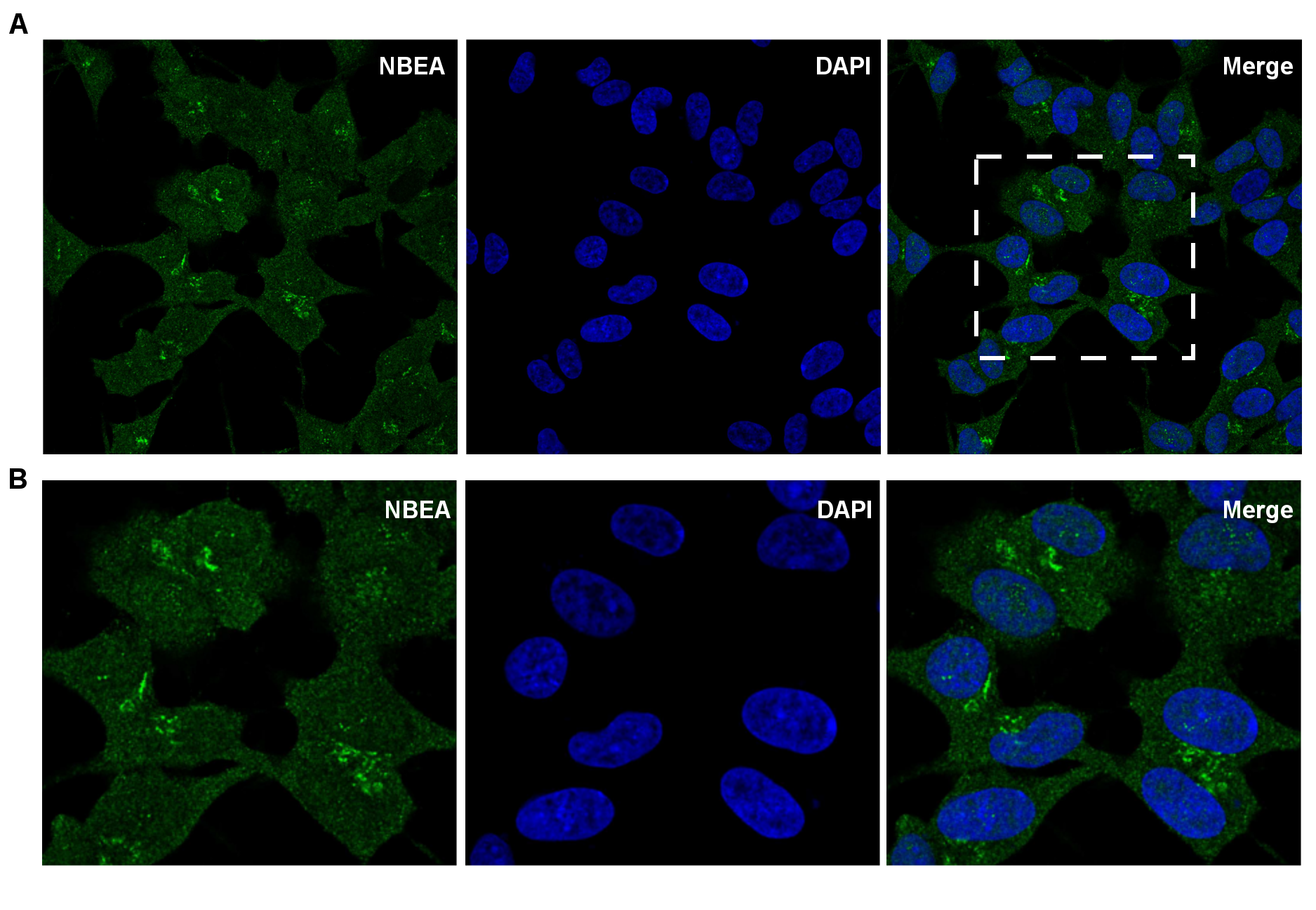

Supplement: S4 Fig — A) Confocal image obtained from the Human protein atlas database (www.proteinatlas.org) of SH-SY5Y, human neuroblast cells for endogenous NBEA (green) immunostaining. DAPI (blue) was used to stain the nucleus. Another antibody was used for NBEA immunostaining than is used in this article, namely HPA040385. B) A larger magnification is shown from the area in the merged panel A, which is marked with a dashed, white line. (TIF) [file pone.0151954.s006.tif]

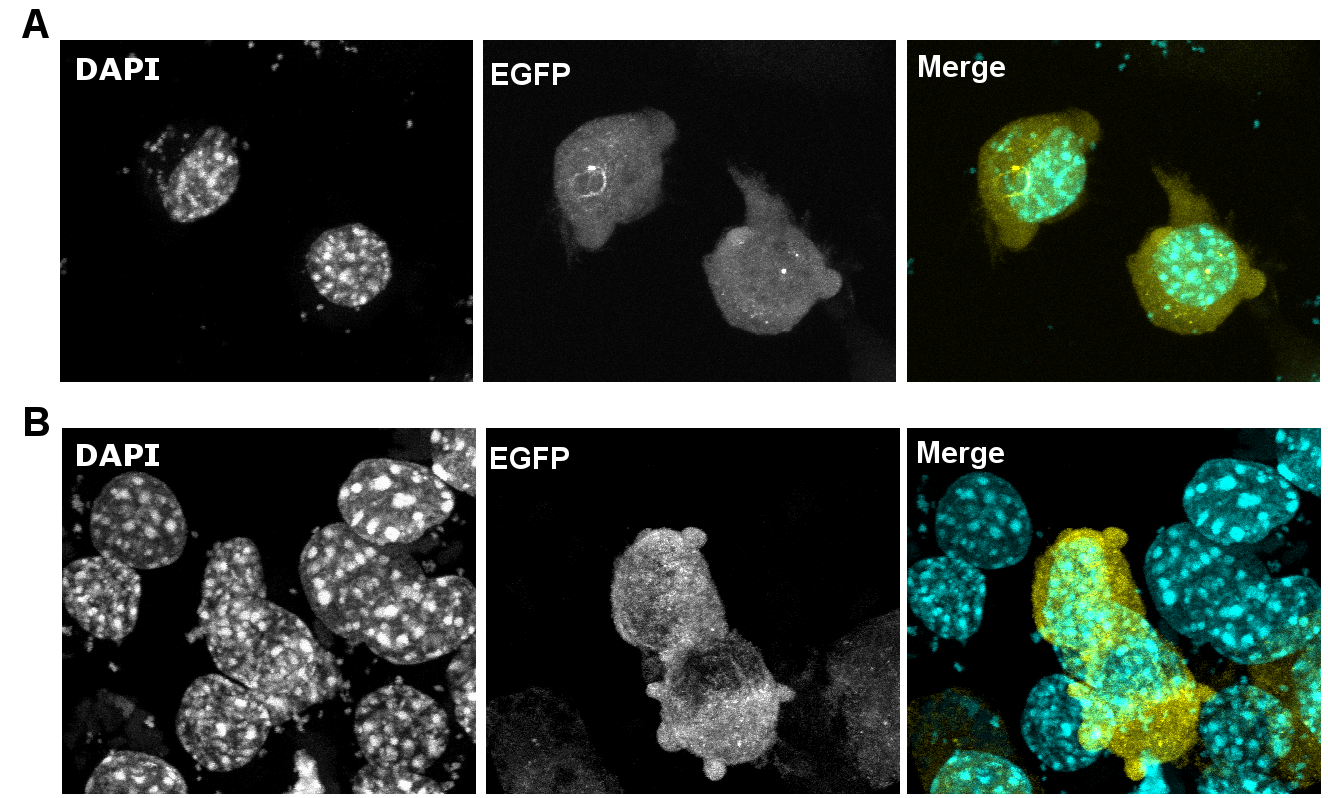

Supplement: S5 Fig — A) A 2D Z-projection of the EGFP-DPBW fusion protein (yellow) in N2a cells is shown. DAPI (cyan) was used to stain the nucleus. The separate images are shown in greyscale. B) A 2D Z-projection of the EGFP-DPBWMUT fusion protein (yellow) in N2a cells is shown. DAPI (cyan) was used to stain the nucleus. The separate images are shown in greyscale. (TIF) [file pone.0151954.s007.tif]

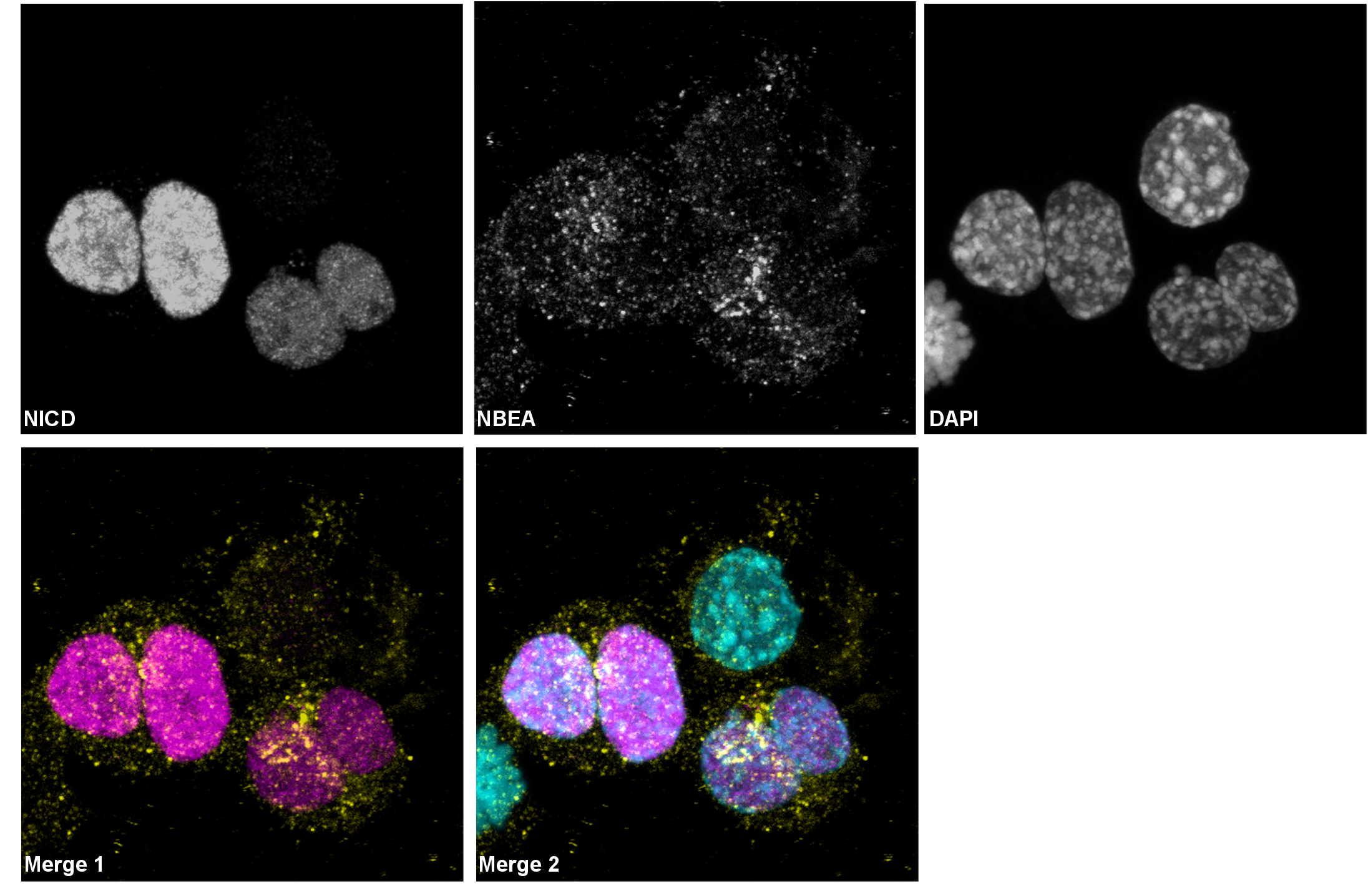

Supplement: S6 Fig — A 2D Z-projection of endogenous NBEA (yellow) and overexpressed NICD (magenta) in N2a cells is shown. DAPI (cyan) was used to stain the nucleus. The separate images are shown in greyscale. (TIF) [file pone.0151954.s008.tif]
